# Characterization of Proanthocyanidin Oligomers of *Ephedra sinica*

**DOI:** 10.3390/molecules22081308

**Published:** 2017-08-06

**Authors:** Joanna Orejola, Yosuke Matsuo, Yoshinori Saito, Takashi Tanaka

**Affiliations:** Department of Natural Product Chemistry, Graduate School of Biomedical Sciences, Nagasaki University, 1-14 Bunkyo-machi, Nagasaki 852-8521, Japan; jjorejola@up.edu.ph (J.O.); y-matsuo@nagasaki-u.ac.jp (Y.M.); saiyoshi@nagasaki-u.ac.jp (Y.S.)

**Keywords:** *Ephedra sinica*, proanthocyanidin, oligomer, thiolysis, phloroglucinolysis, TDDFT, ECD

## Abstract

*Ephedra sinica*, an important plant in Chinese traditional medicine, contains a complex mixture of proanthocyanidin oligomers as major constituents; however, only the minor components have been chemically characterized. In this study, oligomers with relatively large molecular weights, which form the main body of the proanthocyanidin fractions, were separated by adsorption and size-exclusion chromatography. Acid-catalyzed degradation in the presence of mercaptoethanol or phloroglucinol led to the isolation of 18 fragments, the structures of which were elucidated from their experimental and TDDFT-calculated ECD spectra. The results indicated that (−)-epigallocatechin was the main extension unit, while catechin, the A-type epigallocatechin–gallocatechin dimer, and the A-type epigallocatechin homodimer, were identified as the terminal units. Among the degradation products, thioethers of gallocatechin with 3,4-*cis* configurations, a B-type prodelphinidin dimer, a prodelphinidin trimer with both A- and B-type linkages, and a prodelphinidin dimer with an α-substituted A-type linkage were new compounds. In addition, a phloroglucinol adduct of an A-type prodelphinidin dimer, a doubly-linked phloroglucinol adduct of epigallocatechin, and a unique product with a flavan-3-ol skeleton generated by the rearrangement of the aromatic rings were also isolated.

## 1. Introduction

*Ephedra sinica* Stapf (Fam. *Ephedraceae*) is one of the most important plants in traditional medicine, and is used as a diuretic, antipyretic, diaphoretic, and for relieving a cough and asthma [1]. As the crude drug, it has an official monograph in both the Chinese and Japanese Pharmacopoeias, where it is standardized against the major alkaloids, ephedrine and pseudoephedrine [2]. Thus, the main emphasis is conventionally given to its alkaloidal content, despite the fact that this only constitutes about 0.7–0.8% of the whole plant [3,4]. Clearly, the motivation for this is the proven clinical effects of these alkaloids on the respiratory, central nervous, and cardiovascular systems [5]. However, many species of *Ephedra* have also been shown to contain significant amounts of proanthocyanidins [6]. Recently, many health benefits of foods and medicinal plants have been attributed to proanthocyanidins [7], and some of their biological activities, including hypotensive and vasorelaxant effects [8,9], improvement of the airway microenvironment in asthma [10], and the inhibition of inflammation and remodeling in murine models of chronic asthma [11], are responsible for the aforementioned activities of *E. sinica*, especially its respiratory and cardiovascular effects. A number of studies have shown that *Ephedra* spp. also display other biological activities that are not attributed to alkaloids, including antimicrobial [12,13], antioxidant [14], anti-inflammatory [15,16], immunosuppressive [17], antiviral [18], anti-invasive, antiangiogenic, antitumor [19], and cytotoxic [20] properties. The dimeric proanthocyanidins of *E. sinica* show cytotoxic activity against the tumor cell lines SGC-7901, HepG2, and HeLa [21]. In addition, a decrease in the uremic toxin parameters of rats was reportedly induced by the administration of proanthocyanidin oligomers of *E. sinica* [22,23].

**Figure 1:**
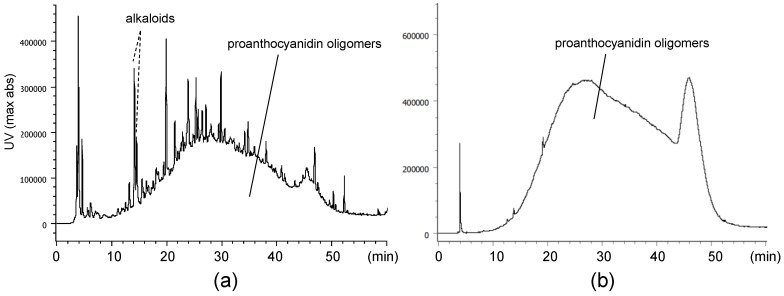
HPLC profiles of 60% EtOH extract of *E. sinica* (**a**) and proanthocyanidin oligomer fraction (**b**).

**Scheme 1:**
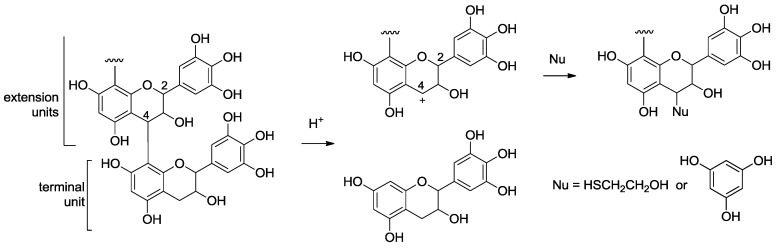
Reaction mechanism of the acid-catalyzed cleavage of the interflavan bond in the presence of nucleophiles (Nu).

As for the composition of the proanthocyanidins of *E. sinica*, monomeric flavan-3-ols [12,24] and dimeric proanthocyanidins with A-type linkages have been isolated [12,21,25,26,27,28]. The presence of prodelphinidin trimers and tetramers with A- and B-type linkages has also been shown [12]. However, these flavan-3-ols and proanthocyanidins are minor components of the total polyphenol content, and our preliminary HPLC and TLC analysis of the extract suggested that the main body of the polyphenols was a complex mixture of oligomers, detected as a broad hump on the HPLC baseline and at the origin of the TLC plate (Figure 1a). Thus, the present study aimed at characterizing these proanthocyanidin oligomers by acid-catalyzed degradation in the presence of nucleophilic agents, that is, 2-mercaptoethanol or phloroglucinol. The degradation involved the cleavage of the interflavan bonds under acidic conditions, generating flavan-3-ols from the terminal units and flavanyl-4 cations from the extension units, which were trapped by nucleophilic agents (Scheme 1) [29].

## 2. Results and Discussion

### 2.1. Composition of the Intact Proanthocyanidin Oligomer

**Figure 2:**
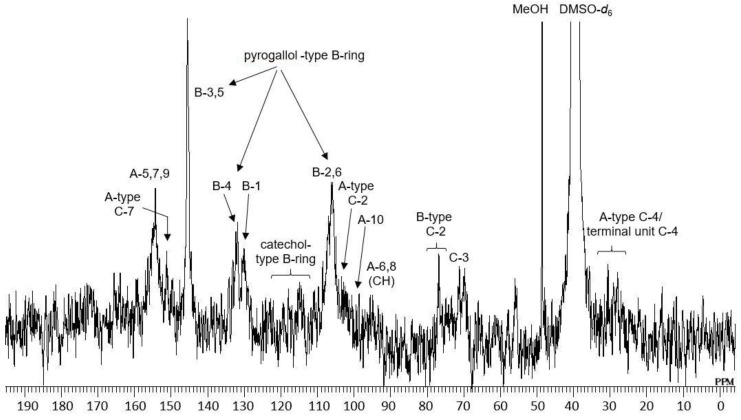
^13^C-NMR spectrum of the proanthocyanidin oligomers from *E. sinica*, measured at 100 MHz in DMSO*-d*_6_*.*

The dried aerial parts of *E. sinica* were extracted with aqueous acetone and fractionated by a series of chromatographic separation methods, including size-exclusion chromatography [30]. The fractions containing only oligomeric proanthocyanidins accounted for 2.7% of the dried plant material, and the HPLC profile showed a broad hump on the baseline (Figure 1b). The ^13^C-NMR spectrum of the oligomer fraction in DMSO-*d*_6_ (Figure 2) showed signals characteristic of proanthocyanidins [31]. Based on a comparison with the literature data [11], the signals at δ_C_ 77 and δ_C_ 70–73, which were attributable to flavan C-ring C-2 and C-3 methine carbons, respectively, suggested the occurrence of B-type linkages. The chemical shifts also indicated that the 2,3-*cis* configuration was more abundant than 2,3-*trans* [32,33]. The signals in the range of δ_C_ 27–31 were attributable to the C-4 carbons of A-type proanthocyanidin extension units [12] and of terminal units [34,35]. The prominent aromatic signals observed at δ_C_ 106, 130, 132, and 145 suggested the predominance of pyrogallol-type B-rings over catechol-type B-rings (δ_C_ 115–120).

### 2.2. Acid-Catalyzed Degradation Products

#### 2.2.1. Identification of Known Products

**Figure 3:**
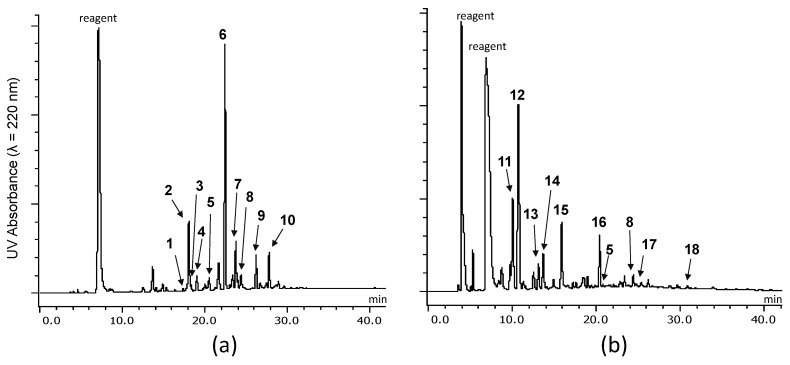
HPLC profiles of thiol degradation products (**a**) and phloroglucinolysis products (**b**)*.*

Thiol degradation was performed according to the previously described method [35] with modifications of the reaction time and temperature, and 10 compounds (**1**–**10**) were isolated and characterized (Figure 3a). Acid-catalyzed degradation with phloroglucinol [29,36] yielded a different set of 10 products (**5**, **8**, **11**–**18**), among which two products (**5** and **8**) were identical to those obtained by thiol degradation (Figure 3b).

**Figure 4:**
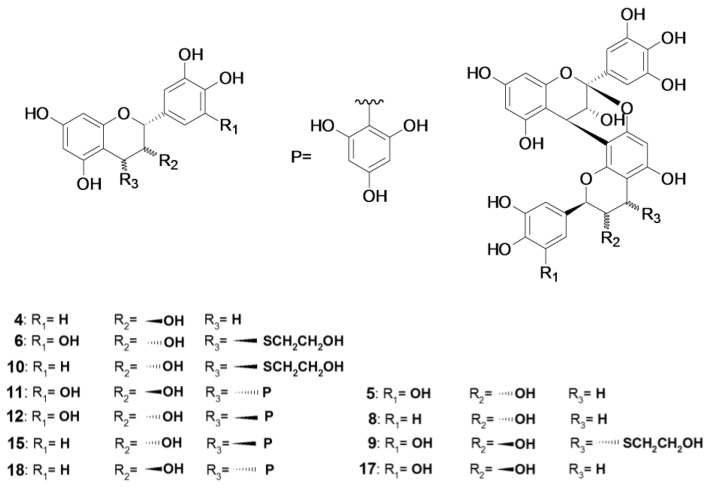
Structures of known degradation products.

Based on a comparison of the ^1^H- and ^13^C-NMR data with those published [12,33], four products were identified as (+)-catechin (**4**), (−)-epigallocatechin-(4β→8,2→*O*→7)-(+)-gallocatechin (**5**), (−)-epigallocatechin-(4β→8,2→*O*→7)-(+)-catechin (**8**), and (−)-epigallocatechin-(4→8,2→*O*→7)-(−)-epigallocatechin (**17**) (Figure 4). These products originated from the terminal units. As depicted in the HPLC profile of the reaction mixture (Figure 3), the peaks attributable to the terminal units were very small compared with those of the extension units, suggesting a high degree of polymerization. The major degradation products of thiolysis **6** and of phloroglucinolysis **12** were identified as epigallocatechin–nucleophile adducts [33,37], indicating that epigallocatechin was the major extension unit of the oligomer. The ^1^H- and ^13^C-NMR spectra of **9**, **10**, **11**, **15**, and **18** were found to be consistent with those previously reported for (−)-epigallocatechin-(4→8,2→*O*→7)-(−)-epigallocatechin-4-(2-hydroxyethyl)-thioether, (−)-epicatechin-4-(2-hydroxyethyl)-thioether, (+)-gallocatechin-4-phloroglucinol, (−)-epicatechin-4-phloroglucinol, and (+)-catechin-4-phloroglucinol, respectively [33,37,38].

#### 2.2.2. Structure Elucidation of New Degradation Products

**Figure 5:**
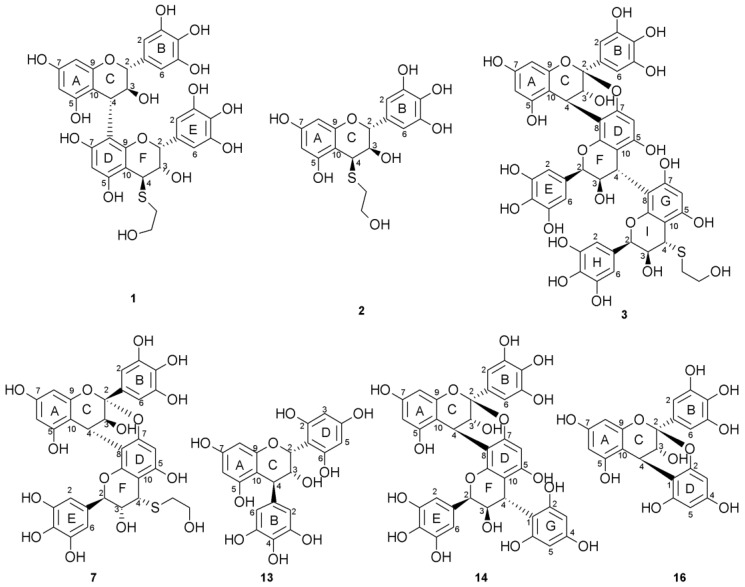
Structures of the new products obtained by the thiolysis and phloroglucinolysis of *E. sinica* proanthocyanidin oligomers.

Among the 18 isolated products, **1**, **2**, **3**, **7**, **13**, **14**, and **16** are reported here for the first time. Their structures are shown in Figure 5 and the ^1^H- and ^13^C-NMR spectroscopic data are summarized in Table 1 and Table 2.

**Figure 6:**
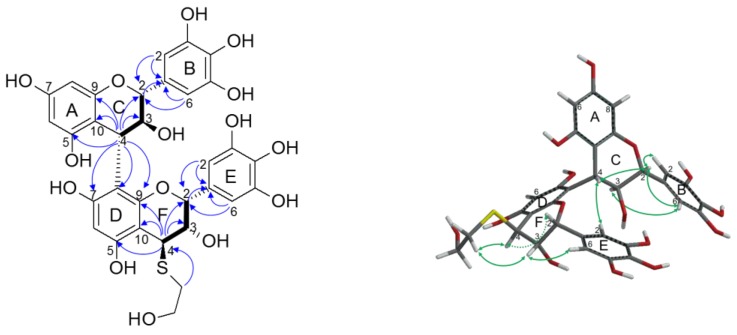

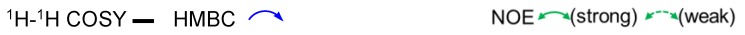
HMBC, ^1^H-^1^H COSY, and NOESY correlations of **1**.

The molecular formula of **1** was shown to be C_32_H_30_O_15_S based on the [M + H]^+^ peak at *m*/*z* 687.1382 in HRFABMS, indicating that **1** was a mercaptoethanol adduct of a prodelphinidin dimer with a B-type linkage. This was confirmed by the appearance of two intense signals at δ_H_ 6.57 and δ_H_ 6.67 (each 2H) arising from two pyrogallol-type B-rings and two methine proton signals attributable to C-ring H-2 in the ^1^H-NMR signals (Table 1). In the HSQC spectrum, the C-ring H-2 signal at δ_H_ 4.34 (*J =* 9.4 Hz) was correlated to a carbon signal at δ_C_ 83.2, while the other F-ring H-2 (Figure 6) at δ_H_ 5.31 (br s) was found to be connected to the carbon that resonated at δ_C_ 75.05. The former indicated a 2,3-*trans* configuration and the latter, a 2,3-*cis* configuration [33]; thus, the dimer was composed of gallocatechin and epigallocatechin. The ^1^H-^1^H COSY and HMBC correlations (Figure 6) allowed the determination of the connectivity of the two catechin units and hydroxyethylthiol group. The strong NOESY correlations between C-ring H-2 and H-4 (Figure 6), and between -SCH_2_- and F-ring H-3, confirmed the configuration of the C- and F-rings [39,40]. A linkage between C-ring C-4 and D-ring C-8 was deduced from the NOESY correlation between aromatic E-ring H-2, 6 and C-ring H-4 [41,42].

ECD spectroscopy allowed the determination of the absolute configuration at C-4. The ^1^H coupling constants of the C-ring indicated that the B-ring was in an equatorial position (*E*-conformer). Taking this observation into account, the negative Cotton effect at 218 nm implied an α-orientation of the terminal unit at C-4 [40]. Thus, the extension unit was concluded to be (+)-gallocatechin. The establishment of the absolute configuration of the epigallocatechin unit relied on the Cotton effect at the ^1^L_b_ band (280 nm) rather than the ^1^L_a_ band (220–240 nm) [40]. Here, the negative Cotton effect at 288 nm, in addition to the predominance of *E*-conformers, both led to the conclusion that the pyrogallol E-ring had an α-orientation relative to the F-2 carbon. The terminal unit was thereby designated as (−)-epigallocatechin. Furthermore, the ECD spectrum for **1** showed a close resemblance to those of procyanidins B-4 previously observed by Barrett and colleagues [43]. Accordingly, **1** was concluded to be (+)-gallocatechin-(4→8)-(−)-epigallocatechin-4-(2-hydroxyethyl)thioether.

**Figure 7:**
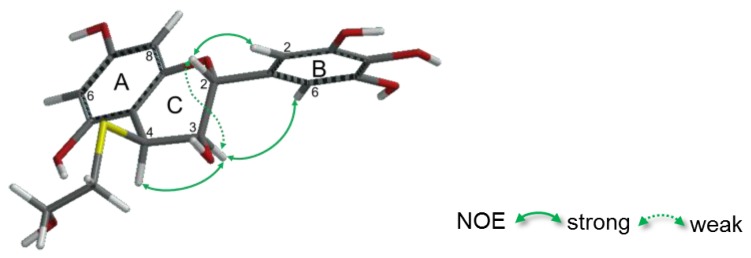
NOE correlations of **2**.

Product **2** showed the [M + H]^+^ peak at *m*/*z* 383.0801 in HRFABMS, confirming the molecular formula as C_17_H_18_O_8_S. The ^1^H-NMR spectrum (Table 1) showed a doublet signal at δ_H_ 4.77 (*J* = 9.6 Hz), indicating the 2,3-*trans* configuration characteristic of gallocatechin. The C-ring H-4 resonated as a doublet at δ_H_ 4.36 (*J =* 4.3 Hz), which indicated the 3,4-*cis* configuration [31]. This was further confirmed by the appearance of a strong NOESY correlation between H-3 and H-4, and the absence of NOE between H-2 and H-4 (Figure 7). As for the absolute configuration, a negative Cotton effect at 284 nm in the ECD spectrum, which was similar to that of (+)-catechin [43], suggested *P-*helicity for the flavan A- and B-rings. Based on these results, **2** was concluded to be (+)-gallocatechin-4-(2-hydroxyethyl)thioether.

**Figure 8:**
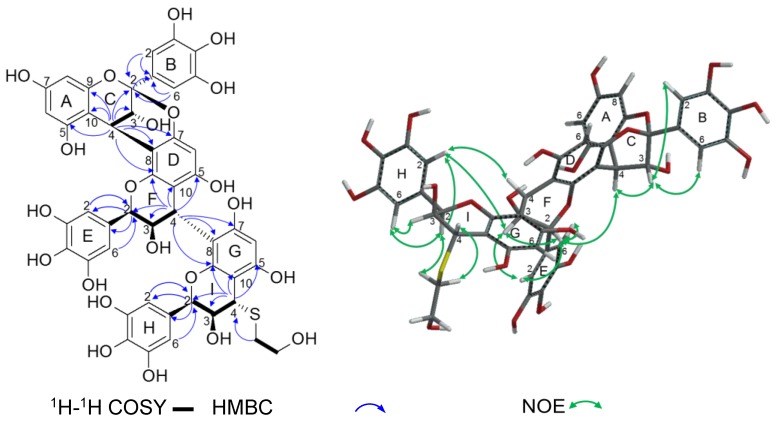
^1^H-^1^H COSY and HMBC correlations (**left**) and NOESY correlations (**right**) of **3**.

Compound **3** was found to have the molecular formula C_47_H_40_O_22_S based on the [M + Na]^+^ peak at *m*/*z* 1011.1639 in HRFABMS. This implied that **3** was a thioether of a prodelphinidin trimer involving both A-type and B-type linkages. In the ^1^H-NMR spectrum (Table 1), three intense aromatic singlets at δ_H_ 6.77, δ_H_ 6.70, and δ_H_ 6.56 (each 2H) indicated the presence of three pyrogallol-type B-rings. The presence of two B-type linkages was apparent from the two C-ring H-2 signals resonating at δ_H_ 5.31 and δ_H_ 5.29 with small *J*_2,3_ values (<2 Hz). This again indicated that the two units were epigallocatechin. These spectroscopic features suggested a close relationship between **3** and the epigallocatechin trimer isolated from *E. sinica* with A- and B-type linkages [12]. In the HMBC spectrum of **3** (Figure 8), the ketal carbon C-2 (δ_C_ 99.91) of the A-type linkage was correlated to C-ring H-4 (δ_H_ 4.38, *J =* 3.4 Hz), which was in turn correlated to a D-ring C-9 (δ_C_ 151.31) of the middle unit. Another benzylic methine H-4 of the middle unit F-ring (δ_H_ 4.79, *J =* 2.2 Hz) showed an HMBC correlation to the D-ring C-9 and terminal unit G-ring C-9 (δ_C_ 153.90). This indicated that an A-type linkage was involved between the top and middle units. The ^13^C-NMR chemical shift for F-ring C-4 at δ_C_ 36.21 was consistent with its involvement in a B-type linkage at this position [12,33,44], and the I-ring C-4 at a lower field (δ_C_ 43.83) was indicative of a thioether at this position [38]. The F-ring H-4 and I-ring H-4 were observed as doublet signals with coupling constants of *J =* 2.3 Hz and *J =* 2.4 Hz, respectively, indicating that both flavan rings adopted a 3,4-*trans* configuration [31]. This was further supported by the absence of a NOESY correlation between H-2 and H-4 in both the F-ring and the I-ring (Figure 8). The linkage between rings C and D was established as 4→8, 2→*O*→7 by the presence of a NOESY correlation between E-ring H-2,6 and C-ring H-4 [41]. The connection between the F-ring and the G-ring was also determined to be from C-4 to C-8 based on the NOESY cross peaks of H-ring H-2,6 with F-ring H-4 and H-3.

**Figure 9:**
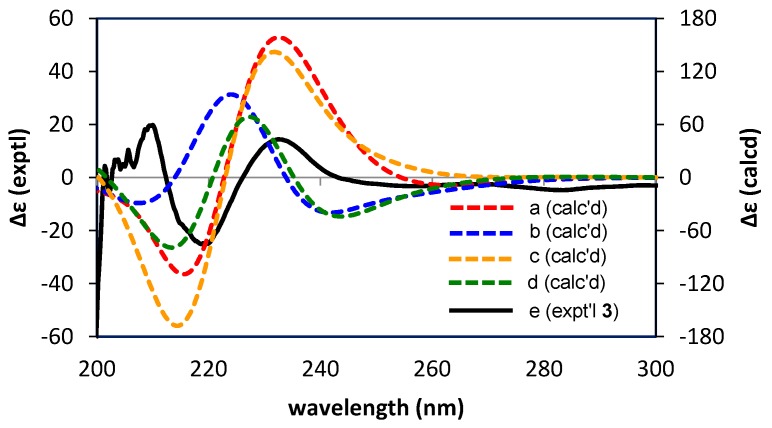
Calculated (a–d) and experimental (e) ECD spectra of prodelphinidin trimer thioether (**3**).

The ECD spectrum of **3** showed a strong positive Cotton effect at 233 nm, reflecting the configuration at C-ring C-4, thereby establishing the top extension unit as (−)-epigallocatechin; however, the configuration of the middle and bottom epigallocatechin units could not be determined from the ECD data. Prodelphinidin oligomers with (+)-epigallocatechin units were previously isolated from the same plant source [12]; therefore, the absolute configuration of **3** was established by TDDFT calculations of the ECD spectra for four stereostructures: (**a**) (−)-epigallocatechin-(−)-epigallocatechin-(−)-epigallocatechin, (**b**) (−)-epigallocatechin-(+)-epigallocatechin-(−)-epigallocatechin, (**c**) (−)-epigallocatechin-(−)-epigallocatechin-(+)-epigallocatechin, and (**d**) (−)-epigallocatechin-(+)-epigallocatechin-(+)-epigallocatechin (Figure 9). The experimental ECD spectrum of **3** (Figure 9**e**) showed a positive Cotton effect at 233 nm and a negative Cotton effect at 218 nm, similar to the Cotton effects observed in the calculated ECD spectra **a** and **c**. This comparison of calculated and experimental spectra revealed that the absolute structure of the upper and middle units in **3** was (−)-epigallocatechin. Moreover, the experimental spectrum **e** contained a weak negative Cotton effect at 250–300 nm, similar to the negative Cotton effect in the calculated ECD spectrum **a**. Therefore, **3** was established as (−)-epigallocatechin-(4β→8,2→*O*→7)-(−)-epigallocatechin-(4β→8)-(−)-epigallocatechin-4-(2-hydroxyethyl)thioether.

**Figure 10:**
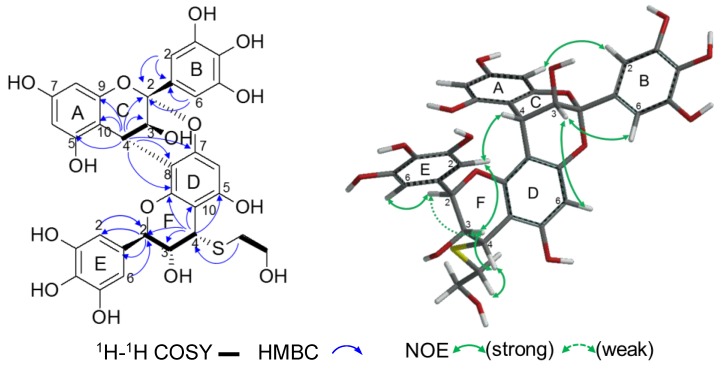
^1^H-^1^H COSY, HMBC, and NOESY correlations of **7***.*

Compound **7** was characterized as an A-type prodelphinidin dimer with a mercaptoethanol substituent, and its molecular formula was determined as C_32_H_28_O_15_S from the [M + Na]^+^ peak at *m*/*z* 707.1043 in HRFABMS. The presence of an A-type linkage was apparent from the signal at δ_C_ 100.07, attributable to the C-ring C-2 ketal carbon [12,42]. The large coupling constant (*J* = 9.8 Hz) of the H-2 at δ_H_ 4.99 indicated the 2,3-*trans* configuration of the lower unit F-ring. The coupling constants of the C-ring H-4 at δ_H_ 4.17 (*J =* 3.6 Hz) and F-ring H-4 at δ_H_ 4.39 (*J =* 4.3 Hz), which were similar to the values observed in **2**, suggested the 3,4-*cis* configuration of these rings. A comparison of the ^1^H- and ^13^C-NMR data with those in the literature suggested that the dimer was composed of epigallocatechin and gallocatechin [12,37]. This was supported by a strong NOE between F-3 and F-4, and weak NOE between F-2 and F-3 (Figure 10)*.* The linkage between the C- and D-rings was established to be 4→8 by the observation of a NOESY correlation between C-ring H-4 and E-ring H-2,6. The absolute configuration at the C-ring C-4 was established by ECD spectroscopy, where the strong negative Cotton effect at 228 nm indicated that the extension unit was (+)-epigallocatechin. The terminal unit was designated as (+)-gallocatechin based on a comparison of the ECD spectrum with that of compound **2**, which also had a negative Cotton effect, of a lesser amplitude, at 284 nm. Compound **7** was thereby established as (−)-epigallocatechin-(4α→8,2→*O*→7)-(+)-gallocatechin-4-(2-hydroxyethyl)thioether.

**Figure 11:**
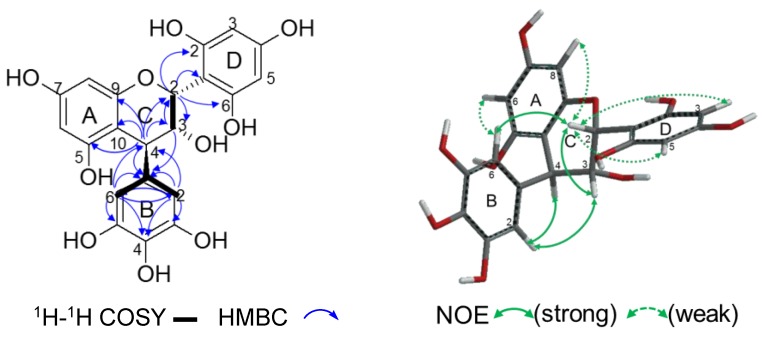
^1^H-^1^H COSY, HMBC, and NOESY correlations of **13**.

**Scheme 2:**
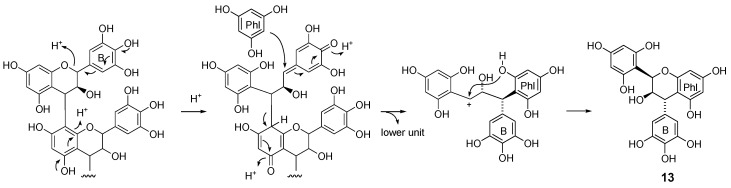
A possible production mechanism of **13**.

Compound **13** was obtained as a product of phloroglucinolysis, and the HRFABMS peak (*m*/*z* 431.0979 [M + H]^+^) confirmed the molecular formula as C_21_H_18_O_10_, the same as that of **11** and **12**. Because of overlapping C-ring proton signals in the ^1^H-NMR spectrum measured in acetone-*d*_6_, the 2D NMR spectra were measured in methanol-*d*_4_ (Table 2). The resulting ^1^H-NMR spectrum showed signals attributable to pyrogallol (δ_H_ 6.19, 2H) and phloroglucinol (δ_H_ 5.79, 2H) rings, as well as mutually *meta*-coupled A-ring H-6 and H-8 (δ_H_ 5.94 and 5.95, *J* = 2.4 Hz), which were related to those observed in the spectra of **11** and **12** [12,37,42]. In the ^1^H-^1^H COSY spectrum (Figure 11), a broad aliphatic singlet signal at δ_H_ 5.53 was correlated to a methine signal at δ_H_ 4.05 (*J* = 2.4, 1.0 Hz), and these signals were attributed to C-ring H-2 and H-3, respectively. The small coupling constant suggested the 2,3-*cis* configuration [33]. Another aliphatic methine signal at δ_H_ 4.06 was assigned to C-ring H-4 based on its HMBC correlations to A-ring C-5, 9 and 10 (Figure 11). H-4 also showed HMBC correlations with pyrogallol H-2,6 (δ_H_ 6.19), indicating that the pyrogallol ring was attached to C-4. This was further supported by the long-range ^1^H-^1^H coupling between C-ring H-4 and pyrogallol B-ring H-2,6 in the ^1^H-^1^H COSY spectrum and the HMBC cross peak between C-ring H-3 and pyrogallol C-1 [45]. The remaining moiety, i.e., the phloroglucinol ring with a symmetrical structure, was shown to be located at C-ring C-2 by the HMBC correlation of C-ring H-2 to the phloroglucinol C-1, 2, and 6. The 2,3-*cis*-3,4-*trans* configuration was inferred by a comparison of the coupling constants with those in the literature [31], and this was further supported by the absence of NOE between H-2 and H-4 and occurrence of the strong NOE between C-ring H-2 and pyrogallol B-ring H-2,6 (Figure 11). The weak correlation observed between the C-ring H-2 and A-ring H-8 protons suggested that H-2 was at the axial position, thereby implying that the C-ring adopted the *E-*conformation. From the positive Cotton effect at 227 nm, the absolute configuration at C-4 was determined to be *S* [31]. Accordingly, **13** was concluded to be 2-(2,4,6-trihydroxyphenyl)-4-(3,4,5-trihydroxyphenyl)-3,4-dihydro-2*H*-1-benzopyran-3,5,7-triol (2*R*,3*R*,4*S*). This compound was a byproduct of phloroglucinolysis, and a plausible production mechanism is proposed in Scheme 2.

**Figure 12:**
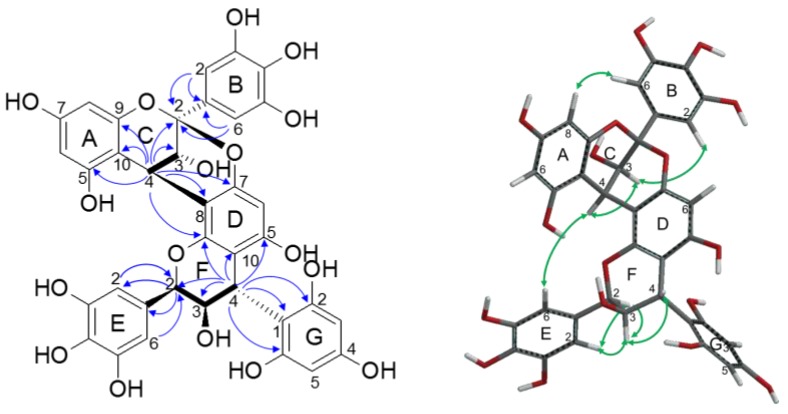

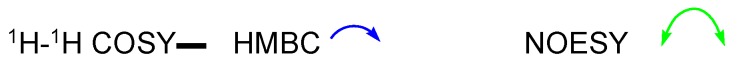
^1^H-^1^H COSY, HMBC, and NOESY correlations of **14**.

Compound **14** was determined to have the molecular formula C_36_H_28_O_17_ (*m*/*z* 733.1406, [M + H]^+^), identifying it as a phloroglucinol adduct of a prodelphinidin dimer involving an A-type linkage. The signals in the ^1^H- and ^13^C-NMR spectra were related to those of the epigallocatechin–epigallocatechin dimer [12] and the procyanidin A2–phloroglucinol adduct [46], and their assignments (Table 2) were based on a comparison with the reported data. The signal of F-ring H-2 (δ_H_ 5.32) was observed as a singlet, indicating the 2,3-*cis* configuration of the F-ring [33]. In addition, the coupling constant of the F-ring H-4 (δ_H_ 4.63, *J* = 2.3 Hz) was consistent with the 3,4-*trans* configuration [31]. This was confirmed by the NOESY spectrum, which displayed a strong correlation between F-ring H-3 and H-4, but no NOE between H-2 and H-4 (Figure 12). The 4→8 linkage between the C- and D-rings was established by the NOE between E-ring H-2,6 and C-ring H-4. Furthermore, the strong positive Cotton effect at 232 nm established that the upper unit was (−)-epigallocatechin [12]. On the basis of previous studies of compound **7**, by Nam et al. [42] and Barrett et al. [43], and considering the weak positive Cotton effect at 220–240 nm of **12**, the lower unit was deduced to be (−)-epigallocatechin. It was therefore concluded that compound **14** was (−)-epigallocatechin-(4β→8,2→*O*→7)-(−)-epigallocatechin-4-phloroglucinol.

**Figure 13:**
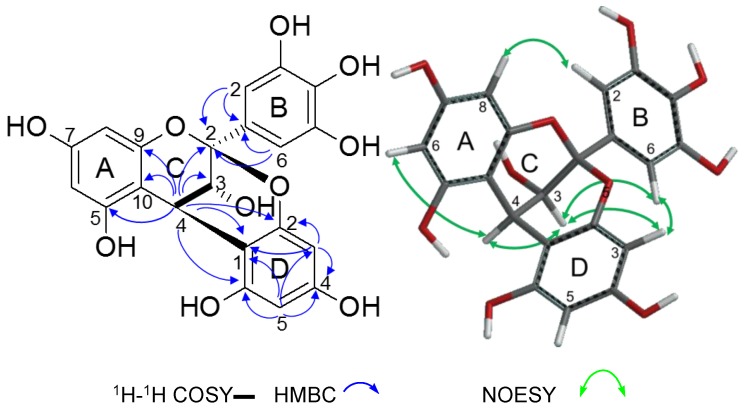
^1^H-^1^H COSY, HMBC, and NOESY correlations of **16**.

**Scheme 3:**
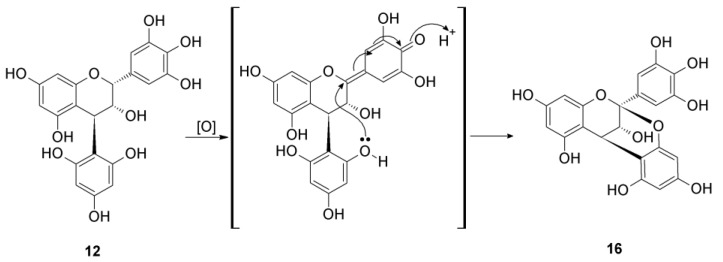
Formation of **16** by oxidative hydride abstraction.

Product **16** showed the [M + H]^+^ peak at *m*/*z* 429.0818 in HRFABMS, indicating the molecular formula C_21_H_16_O_10_. An unambiguous assignment of the ^1^H- and ^13^C-NMR signals was achieved by ^1^H-^1^H COSY, HSQC, HMBC, and NOESY spectroscopy. The absence of a C-2 proton signal and appearance of a C-2 carbon signal at δ_C_ 100.21 confirmed the presence of an A-type linkage [12,44,46]. The HMBC cross peaks (Figure 13) between C-ring H-4 and D-ring C-1,2,6 indicated the linkage of the phloroglucinol moiety to C-ring C-4. The NOE cross peaks between H-3 and B-ring H-2,6 indicated the 3,4-*trans* configuration [47]. Furthermore, the ECD spectrum showed a positive Cotton effect at 220–240 nm, indicating a β- configuration at C-4. Accordingly, **16** was established to be (−)-epigallocatechin-(4β→1,2→*O*→2)-phloroglucinol. Compound **16** was regarded as a byproduct of phloroglucinolysis involving the oxidation of the pyrogallol-type B-ring (Scheme 3) [45].

## 3. Materials and Methods

### 3.1. General

NMR spectra were recorded in acetone-*d*_6_ (Wako Pure Chem. Ind. Ltd., Osaka, Japan), methanol-*d*_4_ (Kanto Chem. Co., Inc., Tokyo, Japan), and DMSO-*d*_6_ (Kanto Chemical Co., Inc., Tokyo, Japan) with a Varian Unity Plus 500 spectrometer (Palo Alto, CA, USA) operating at 500 MHz for ^1^H and 125 MHz for ^13^C, and with a JEOL JNM-AL 400 spectrometer (JEOL Ltd, Tokyo, Japan) at 400 MHz for ^1^H and 100 MHz for ^13^C. HRFABMS spectra were recorded on a JMS 700N spectrometer (JEOL Ltd., Tokyo, Japan) in positive ion mode, with glycerol or *m-*nitrobenzyl alcohol, with or without NaCl, as the matrix. UV spectra were recorded in MeOH with a Jasco V-560 UV/Vis spectrometer (Jasco Co. Ltd., Tokyo, Japan). The same solvent was used for the ECD spectroscopic analysis using a Jasco-725N spectrometer (Jasco Co. Ltd., Tokyo, Japan), and optical rotation measurement using a Jasco P-1020 (Jasco Co. Ltd., Tokyo, Japan). IR spectra were recorded using a Jasco FT/IR-410K (Jasco Co. Ltd., Tokyo, Japan). Column chromatography was performed using a Sephadex LH-20 (25–100 mm, GE Healthcare UK Ltd., Buckinghamshire HP7 9NA, UK), a Diaion HP20SS (Mitsubishi Chemical Co., Tokyo, Japan), and a Chromatorex ODS (Fuji Silysia Chemical Ltd., Kasugai, Japan). TLC was performed on 0.25-mm thick, precoated silica gel 60 F_254_ (Merck, Darmstadt, Germany) with toluene–ethyl formate–formic acid (1:7:1, *v*/*v*) as the solvent system. Spots were detected by illumination under a short wavelength UV (254 nm) followed by spraying with 2% ethanolic FeCl_3_. Analytical HPLC was performed with gradient elution from 4–30% (39 min), 30–75% (15 min), 75–95% (6 min) acetonitrile (Kanto Chemical Co., Inc., Tokyo, Japan) in 50 mM phosphoric acid (Kishida Chemical Co., Osaka, Japan) on a Cosmosil 5C_18_-ARII 4.6 × 250 mm column (Nacalai Tesque, Inc., Kyoto, Japan) at a flow rate of 0.8 mL/min, using an HPLC system composed of a Jasco DG-2080-53 Plus degasser, Jasco PU-2080 Plus pump, Jasco AS-2055 Plus autosampler, Jasco CO-2065 Plus column oven (maintained at 35 °C), and Jasco MD-2018 Plus PDA detector (Jasco Co. Ltd., Tokyo, Japan).

### 3.2. Plant Material

Dried aerial parts of *Ephedra sinica* were purchased from Uchida Wakanyaku Ltd., Tokyo, Japan.

### 3.3. Extraction and Isolation

The dried aerial parts (500 g) of *E. sinica* were extracted with 70% acetone (3 L) at room temperature overnight, three times. The extracts were combined and concentrated by rotary evaporation under reduced pressure at 40 °C. The resulting aqueous solution was charged into a Sephadex LH-20 (5 cm × 19 cm) and eluted with H_2_O-MeOH (0–100%, 20% stepwise gradient) and 60% acetone to give two fractions: Fr. 1 and 2 (Figure S1). The first fraction eluted with H_2_O was acidified with trifluoroacetic acid and loaded into a Diaion HP20SS column (5 cm × 30 cm). After washing out sugars and inorganic substances with H_2_O, the column was eluted with 0–100% MeOH (10% stepwise gradient) and then 60% acetone to give Fr. 1-1 (9.16 g) containing proanthocyanidin oligomers and Fr. 1-2 (14.57 g) containing oligomers and low-molecular weight proanthocyanidins. A portion (5 g) of Fr. 1-2 was separated by size-exclusion column chromatography using a Sephadex LH-20 (4 cm × 45 cm) with a mixture of acetone and 7 M urea (3:2, *v*/*v*, containing conc. HCl 5 mL/L) to afford Fr. 1-2-1 containing oligomers and Fr. 1-2-2 containing low-molecular weight polyphenols. After the removal of acetone by evaporation, the resulting aqueous solution of Fr. 1-2-1 was subjected to Diaion HP20SS (3 cm × 19 cm) column chromatography, and urea and HCl were washed out by elution with H_2_O. Subsequent elution of the column with 0–100% MeOH (10% stepwise gradient) yielded Fr. 1-2-1-1 (1.11 g) containing oligomeric proanthocyanidins. Separately, Fr. 2 was subjected to size-exclusion chromatography in a manner similar to that described for Fr. 1-2 to give three fractions. The resulting Fr. 2-2 was loaded into a Diaion HP20SS column to remove urea and HCl, yielding oligomeric proanthocyanidins (Fr. 2-2-2, 3.36 g).

### 3.4. Thiolysis

Thiol degradation was performed according to the method of Kusano et al. [35] with modifications. Proanthocyanidin oligomers (Fr. 1-1, 1.0 g) were dissolved in 60% EtOH (200 mL) containing mercaptoethanol (10 mL) (Kanto Chemical Co. Inc., Tokyo, Japan) and concentrated HCl (0.5 mL) (Kishida Chemical Co., Osaka, Japan). The reaction mixture was then heated at 70 °C for 22 h. The reaction mixture was then analyzed by HPLC and was further fractionated. Fr. 2-2-2 (1.0 g) was also subjected to thiolysis in the same manner. The reaction mixture of Fr. 1-1 was first concentrated to remove EtOH. The resulting aqueous solution was subjected to Sephadex LH-20 chromatography (3 cm × 24 cm), and mercaptoethanol was washed out with H_2_O. Further elution of the column with increasing proportions of MeOH in H_2_O (0–50%, 5% stepwise gradient; 50–100%, 10% stepwise gradient) gave eight fractions. Fr 1-1-5 (0.35 g) was loaded into a Diaion HP20SS (3 cm × 22 cm) with H_2_O-MeOH to furnish five subfractions. Purification of Fr. 1-1-5-4 (41.9 mg) by Sephadex LH-20 chromatography (3 cm × 25 cm) with a systematic stepwise gradient of EtOH-H_2_O-acetone (1:0:0, 9:1:0, 8:2:0, 6:4:0, 54:36:10, 48:32:20, 36:24:40, 0:50:50, *v*/*v*) enabled the isolation of **10** (19.5 mg). Separation of Fr. 1-1-5-3 (178.7 mg) with the same chromatographic procedure afforded compounds **4** (4.9 mg) and **6** (60.8 mg). Fr. 1-1-5-2 (72.9 mg) was separated by Chromatorex ODS chromatography (3 cm × 17 cm) with H_2_O-MeOH and a Sephadex LH-20 (2.5 cm × 13 cm) with H_2_O-MeOH to afford **6** (21.0 mg), **1** (5.2 mg), and **2** (21.3 mg). Separation of Fr. 1-1-7 by Diaion HP20SS chromatography (2 × 17 cm) with H_2_O-MeOH afforded six subfractions, and Fr. 1-1-7-3 (104.3 mg) was further separated by Chromatorex ODS chromatography (2 cm × 16 cm) with H**_2_**O-MeOH to give **3** (21.2 mg), **7** (19.3 mg), and **9** (9.0 mg). The thiol degradation products of the other oligomers, Fr. 2-2-2, were also fractionated in the same manner as described for Fr. 1-1 to give seven subfractions. Fr. 2-2-2-6 (241.5 mg) was separated by Diaion HP20SS column chromatography (3 cm × 16 cm) with H_2_O-MeOH (0–100%, 20% stepwise gradient). Among the six subfractions obtained, the separation of Fr. 2-2-2-6-3 (74.5 mg) by a Chromatorex ODS (2.5 cm × 15 cm) with H_2_O-MeOH yielded **5** (0.6 mg), **7** (36.2 mg), and **9** (10.0 mg). The same chromatographic procedure using a Chromatorex ODS was applied to the separation of Fr. 2-2-2-6-4 (49.3 mg), which yielded **9** (21.0 mg) and a crude crop of **8**. The latter was purified by Sephadex LH-20 (2 cm × 16 cm) and a system of increasing MeOH concentration in H_2_O (0–40%, 20% stepwise gradient; 40–100%, 5% stepwise gradient), which led to the isolation of **8** (8.8 mg).

*(+)-Gallocatechin-(4α→8)-(−)-epigallocatechin-4-(2-hydroxyethyl)thioether* (**1**): yellowish brown amorphous powder; ${\left[\alpha \right]}_{D}^{16}$ −105.9 (*c* = 0.10, MeOH); UV (MeOH) λ_max_ (log ε) 270 (3.61), 239 (4.41), 214 (5.00) nm; CD (MeOH) Δε_218_ −33.1, Δε_288_ −2.3; IR ν_max_ 3311, 1609, 1449, 1541, 1449 cm^−1^; HRFABMS *m*/*z* 687.1382 [M + H]^+^ (calcd for C_32_H_31_O_15_S, 687.1378); ^1^H- and ^13^C-NMR data, see Table 1.

*(+)-Gallocatechin-4-(2-hydroxyethyl)thioether* (**2**): pale brown amorphous powder; ${\left[\alpha \right]}_{D}^{16}$ +29.9 (*c* = 0.13, MeOH); UV (MeOH) λ_max_ (log ε) 273 (3.17), 235 (4.42), 209 (4.72) nm; CD (MeOH) Δε_220_ −2.8, Δε_249_ +2.2, Δε_284_ −0.4; IR ν_max_ 3344, 1621, 1537, 1515, 1455, 1345 cm^−1^; HRFABMS *m*/*z* 383.0801 [M + H]^+^ (calcd for C_17_H_19_O_8_S, 383.0795); ^1^H- and ^13^C-NMR data, see Table 1.

*(−)-Epigallocatechin-(4β→8,2→O→7)-(−)-epigallocatechin-(4α→8)-(−)-epigallocatechin-4-(2-hydroxyethyl) thioether* (**3**): reddish brown amorphous powder; ${\left[\alpha \right]}_{D}^{20}$ −17.2 (*c* = 0.11, MeOH); UV (MeOH) λ_max_ (log ε) 270 (3.83), 241 (4.65), 205 (5.31) nm; CD (MeOH) Δε_218_ −25.0, Δε_233_ +14.2, Δε_284_ −4.3; IR ν_max_ 3276, 1615, 1541, 1445, 1348 cm^−1^; HRFABMS *m*/*z* 1011.1639 [M + Na]^+^ (calcd for C_47_H_40_O_22_SNa, 1011.1624); ^1^H- and ^13^C-NMR data, see Table 1.

*(+)-Epigallocatechin-(4α→8,2α→O→7)-(+)-gallocatechin-4-(2-hydroxyethyl)thioether* (**7**): pale brown amorphous powder; ${\left[\alpha \right]}_{D}^{17}$ −50.1 (*c* = 0.15, MeOH); UV (MeOH) λ_max_ (log ε) 270 (3.55), 247 (4.32), 208 (5.00) nm; CD (MeOH) Δε_228_ −25.6, Δε_252_ +3.7, Δε_284_ −3.0; IR ν_max_ 3389, 1612, 1537, 1502, 1447, 1333 cm^−1^; HRFABMS *m*/*z* 707.1043 [M + Na]^+^ (calcd for C_32_H_28_O_15_SNa, 707.1041); ^1^H- and ^13^C-NMR data, see Table 1.

### 3.5. Phloroglucinolysis

Fr. 1-1 was subjected to phloroglucinolysis according to the method reported by Kennedy and Jones and Bautista-Ortin and colleagues [18,36], with a few modifications. Fr. 1-1 (2.0 g) was dissolved in MeOH (200 mL) and then mixed with phloroglucinol reagent (200 mL). This phloroglucinol reagent was a methanolic solution containing phloroglucinol (20 g) (Sigma Chemical Co., St. Louis, MO, USA), ascorbic acid (4 g) (Kishida Chemical Co., Osaka, Japan), and concentrated HCl (2.92 mL). The mixture was heated at 50 °C for 60 min, and the reaction was then terminated by the addition of 0.2 M sodium acetate (800 mL). The reaction mixture was concentrated in vacuo to remove MeOH and acidified to pH 4 prior to fractionation. The aqueous solution was loaded into a Sephadex LH-20 column (3 cm × 24 cm) and eluted with H_2_O containing increasing proportions of MeOH to give 10 fractions. Fr. 1-1-5 (810.7 mg) was separated by Diaion HP 20SS column chromatography (3 cm × 21 cm) with H_2_O-MeOH to yield **12** (84.2 mg), **16** (103.7 mg), and five subfractions. Fr. 1-1-5-3 (454.4 mg) was successively separated by a Sephadex LH-20 (H_2_O-MeOH) and a Chromatorex ODS (H_2_O-MeOH) to afford **11** (24.4 mg). From Fr. 1-1-5-5 (59.8 mg), **17** (6.8 mg) was isolated by Chromatorex ODS chromatography (H_2_O-MeOH). Purification of 1-1-6 (102.3 mg) by Diaion HP20SS column chromatography (3 cm × 26 cm) with 0–100% MeOH in H_2_O yielded **13** (10.7 mg). Fractionation of 1-1-7 (120 mg) on a Diaion HP20SS (2 cm × 17 cm) with H_2_O-MeOH gave **5** (9.3 mg) and nine subfractions. Purification of Fr. 1-1-7-2 (6.3 mg) and Fr. 1-1-7-7 (11.4 mg) by a Chromatorex ODS (H_2_O-MeOH) furnished **13** (1.2 mg) and **17** (3.2 mg), respectively. Fr. 1-1-8 (210.7 mg) was separated by Diaion HP20SS chromatography to yield **8** (16.3 mg) and five subfractions, and subfraction 1-1-8-1 (59.4 mg) was further subjected to purification using a Sephadex LH 20 (2 cm × 16 cm) with EtOH-H_2_O-acetone (1:0:0, 9:1:0, 8:2:0, 6:4:0, 54:36:10, 48:32:20, 36:24:40, 0:50:50, *v*/*v*) and then a Chromatorex ODS (H_2_O-MeOH) to give **14** (39.6 mg). Purification of Fr. 1-1-9 (204.8 mg) by Diaion HP 20SS chromatography (2 × 17 cm) with H_2_O-MeOH resulted in the isolation of **18** (11.4 mg).

*2-(2,4,6-Trihydroxyphenyl)-4-(3,4,5-trihydroxyphenyl)-3,4-dihydro-2H-1-benzopyran-3,5,7-triol (2R,3R,4S)* (**13**): pale brown amorphous powder; ${\left[\alpha \right]}_{D}^{20}$ −15.0 (*c* = 0.11, MeOH); UV (MeOH) λ_max_ (log ε) 270 (3.50), 234 (4.40), 210 (4.85) nm; CD (MeOH) Δε_227_ +0.8, Δε_267_ +1.2; IR ν_max_ 3333, 1614, 1522, 1462, 1328 cm^−1^; HRFABMS *m*/*z* 431.0979 [M + H]^+^ (calcd for C_21_H_19_O_10_, 431.0973); ^1^H- and ^13^C-NMR data, see Table 2.

*(−)-Epigallocatechin-(4β→8,2→O→7)-(−)-epigallocatechin-4-phloroglucinol* (**14**): pale brown amorphous powder; ${\left[\alpha \right]}_{D}^{19}$ +53.1 (*c* = 0.14, MeOH); UV (MeOH) λ_max_ (log ε) 270 (3.62), 235 (4.39), 214 (4.84) nm; CD (MeOH) Δε_232_ +18.9, Δε_270_ +1.5, Δε_283_ −1.2; IR ν_max_ 3297, 1625, 1476, 1347 cm^−1^; HRFABMS *m*/*z* 733.1406 [M + H]^+^ (calcd for C_36_H_29_O_17_, 733.1399); ^1^H- and ^13^C-NMR data, see Table 2.

*(−)-Epigallocatechin-(4β→1,2→O→2)-phloroglucinol* (**16**): pale yellow amorphous powder; ${\left[\alpha \right]}_{D}^{19}$ −6.6 (*c* = 0.14, MeOH); UV (MeOH) λ_max_ (log ε) 270 (3.62), 235 (4.39), 214 (4.84) nm; CD (MeOH) Δε_216_ +4.2, Δε_246_ +1.2, Δε_270_ −1.6; IR ν_max_ 3297, 1625, 1476, 1347 cm^−1^; HRFABMS *m*/*z* 429.0818 [M + H]^+^ (calcd for C_21_H_17_O_10_, 429.0816); ^1^H- and ^13^C-NMR data, see Table 2.

### 3.6. Calculations of ECD Spectra

A conformational search was performed using the Monte Carlo method with the MMFF94 force field using Spartan’14 (Wavefunction, Irvine, CA, USA). The resulting low-energy conformers within 6 kcal/mol of the global minimum were optimized at the B3LYP-SCRF/6-31G(d,p) level in MeOH (PCM). The vibrational frequencies were also calculated at the same level to confirm that they were true minima, and no imaginary frequencies were found. The energies, oscillator strengths, and rotational strengths of the low-energy conformers with Boltzmann populations greater than 1% were calculated using TDDFT at the CAM-B3LYP-SCRF/6-31G(d,p) level in MeOH (PCM). The ECD spectra were simulated by overlapping Gaussian functions with a 0.3 eV exponential half-width, and red-shifted by 25 nm. The calculated data for each conformer were averaged according to the Boltzmann distribution theory at 298 K based on their relative Gibbs free energies. All DFT calculations were performed using Gaussian 09 [48].

## 4. Conclusions

The proanthocyanidins of *E. sinica* are mainly composed of oligomers, and in this study, the oligomers were separated and chemically characterized for the first time. Acid-catalyzed degradation with mercaptoethanol and phloroglucinol afforded 18 products, among which seven were previously unreported compounds. Epigallocatechin was the major extension unit, and catechin and A-type prodelphinidin dimers were identified as terminal units. The new compounds were characterized by spectroscopic analyses, and the stereochemistry of the trimeric products was determined with the aid of TDDFT calculations of the ECD spectra. Since *E. sinica* is one of the most important crude drugs in East Asia, and proanthocyanidins are major constituents with a comparable abundance to alkaloids, our results provide important insights into the molecular basis of traditional medicine.

## Figures and Tables

**Table 1:** ^1^H- (500 MHz) and ^13^C- (125 MHz) NMR data of **1**, **2**, **3**, and **7** in acetone-*d*_6_.

		1				2				3				7		
position		δ_H_	( *J* in Hz)	δ_C_		δ_H_	( *J* in Hz)	δ_C_		δ_H_	( *J* in Hz)	δ_C_		δ_H_	( *J* in Hz)	δ_C_
C	2	4.34	d (9.5)	83.19		4.77	d(9.7)	78.77				99.91				100.07
	3	4.50	dd (7.8, 9.5)	73.26		4.07	dd(9.7, 4.4)	71.59		4.20	d (3.4)	67.31		4.15	d (3.6)	66.82
	4	4.68	d (7.8)	38.11		4.36	d(4.4)	44.90		4.38	d (3.4)	28.68		4.17	d (3.6)	28.47
A	5			158.27				157.51				156.59				156.04
	6	5.82	s	97.04		6.00	d(2.3)	96.67		5.85	d (2.4)	97.58		5.89	d (2.3)	97.62
	7			157.15				159.14				157.69				157.88
	8	5.82	d (1.7)	95.82		5.78	d(2.3)	94.96		6.05	s	96.00		6.02	d (2.3)	96.12
	9			156.93				155.88				151.65				153.76
	10			106.09				102.14				103.92				103.38
B	1			131.56				130.36				131.50				130.96
	2	6.57	s	107.94		6.50	s	108.19		6.77	s	107.23		6.74	s	107.23
	3			146.16				146.08				145.63				145.64
	4			133.28				133.49				133.74				133.81
	5			146.16				146.08				145.63				145.64
	6	6.57	s	107.94		6.50	s	108.19		6.77	s	107.23		6.74	s	107.23
F	2	5.31	br s	75.05						5.31	br s	78.02		4.99	d (9.8)	79.57
	3	4.04	d (1.1)	71.95						3.95	d (2.2)	72.84		4.21	dd (9.8, 4.3)	71.02
	4	4.05	d (1.1)	43.67						4.79	d (2.2)	36.21		4.39	d (4.3)	44.43
D	5			156.49								155.79				155.74
	6	6.04	s	97.69						6.05	s	96.00		6.13	s	96.92
	7			156.49								153.78				153.02
	8			108.15								106.13				105.98
	9			154.34								151.31				149.62
	10			98.91								104.48				104.33
E	1			130.97								130.62				129.08
	2	6.67	s	106.11						6.56	s	106.42		6.64	s	108.00
	3			145.94								146.27				146.32
	4			132.60								133.08				134.05
	5			145.94								146.27				146.32
	6	6.67	s	106.11						6.56	s	106.42		6.64	s	108.00
I	2									5.29	br s	75.06				
	3									4.14	d (2.3)	71.18				
	4									4.12	d (2.3)	43.83				
G	5											156.77				
	6									5.96	s	97.45				
	7											157.05				
	8											106.91				
	9											153.90				
	10											99.96				
H	1											130.93				
	2									6.70	s	106.42				
	3											146.14				
	4											132.74				
	5											146.14				
	6									6.70	s	106.42				
CH_2_OH-		3.73-3.89	m	62.65		3.68-3.88	m	62.68		3.73-3.94	m	62.72		3.73-3.84	m	62.47
SCH_2_-		2.75-2.96	m	34.95		2.75-3.11	m	37.22		2.78-2.99	m	35.14		2.77-3.09	m	37.54

**Table 2:** ^1^H- (500 MHz) and ^13^C- (125 MHz) NMR data of **13** in methanol-*d*_4_, **14** and **16** in acetone-*d*_6_.

		13				14				16		
position		δ _H_	(*J* in Hz)	δ_C_		δ_H_	(*J* in Hz)	δ_C_		δ_H_	(*J* in Hz)	δ_C_
C	2	5.53	br s	70.22				99.97				100.21
	3	4.05	dd (2.4,1.0)	74.27		4.22	d (3.5)	67.26		4.15	d (3.6)	66.99
	4	4.06	br s	45.70		4.38	d (3.5)	28.66		4.25	d (3.6)	28.74
A	5			158.59				156.60				155.32
	6	5.95	d (2.4)	96.59		5.84	d (2.4)	97.58		6.04	d (1.5)	97.21
	7			158.05				157.65				157.69
	8	5.94	d (2.4)	95.24		6.04	d (2.4)	96.17		6.07	br s	96.21
	9			157.76				153.79				153.96
	10			102.49				104.06				104.26
B	1			135.98				131.53				131.23
	2	6.19	s	108.50		6.78	s	107.29		6.53	s	107.32
	3			146.47				145.62				145.62
	4			132.19				133.73				133.76
	5			146.47				145.62				145.62
	6	6.19	s	108.50		6.78	s	107.29		6.53	s	107.32
F	2					5.32	br s	78.12				
	3					3.95	d (2.3)	72.61				
	4					4.63	d (2.3)	36.24				
D	1			104.59								106.96
	2			158.59								153.81
	3	5.79	s	95.95						5.96	d (1.5)	96.01
	4			159.52								158.18
	5	5.79	s	95.95				156.16		6.03	d (1.5)	97.19
	6			158.59		6.02	s	95.63				154.23
	7							151.54				
	8							105.79				
	9							157.65				
	10							104.58				
E	1							130.96				
	2					6.58	s	106.42				
	3							146.27				
	4							133.02				
	5							146.27				
	6					6.58	s	106.42				
G	1							106.90				
	2							151.31				
	3					6.05	s	95.96				
	4							155.43				
	5					6.05	s	95.96				
	6							151.31				

## Supplementary Materials

The following are available online: Figure S1: Fractionation of proanthocyanidin oligomers of *E. sinica*, Figures S2–S4, Scheme for isolation of acid-catalyzed degradation products of *E. sinica*, Figures S5–S53, IR, ^1^H-NMR, ^13^C-NMR, ^1^H-^1^H COSY, HSQC, HMBC, and NOE spectra of compounds **1**, **2**, **3**, **7**, **13**, **14**, and **16**.
