# Supplementary material for: Characterization of Proanthocyanidin Oligomers of Ephedra sinica
Source: Molecules. 2017 Aug 6;22(8):1308. doi: 10.3390/molecules22081308 (PMC6152249; doi:10.3390/molecules22081308)
Supplement: Supplementary file 1 [file molecules-22-01308-s001.pdf]

Supporting Information

Characterization of Proanthocyanidin Oligomers of *Ephedra sinica*

Joanna Orejola†; Yosuke Matsuo†; Yoshinori Saito†; Takashi Tanaka†

†Laboratory of Natural Product Chemistry, Graduate School of Biomedical Sciences, Nagasaki University, 1-14 Bunkyo-Machi Nagasaki 852-8521, Japan

S3 **Figure S1.** Fractionation of Proanthocyanidin Oligomers of *E. sinica*
S4 **Figure S2.** Isolation of Thiol Degradation Products of *E. sinica* Fr. 1-1
S5 **Figure S3.** Isolation of Thiol Degradation Products of *E. sinica* Fr. 2-2-2
S6 **Figure S4.** Isolation of Phloroglucinol Degradation Products of *E. sinica* Fr. 1-1
S7 **Figure S5.** IR Spectrum of **1**
S8 **Figure S6.** ^{1}H-NMR Spectrum of **1** in acetone-d_6-D_2O (500 MHz)
S9 **Figure S7.** ^{13}C-NMR Spectrum of **1** in acetone-d_6-D_2O (125 MHz)
S10 **Figure S8.** ^{1}H-^{1}H-COSY Spectrum of **1** in acetone-d_6-D_2O
S11 **Figure S9.** HSQC Spectrum of **1** in acetone-d_6-D_2O
S12 **Figure S10.** HMBC Spectrum of **1** in acetone-d_6-D_2O
S13 **Figure S11.** NOE Spectrum of **1** in acetone-d_6-D_2O
S14 **Figure S12.** IR Spectrum of **2**
S15 **Figure S13.** ^{1}H-NMR Spectrum of **2** in acetone-d_6-D_2O (500 MHz)
S16 **Figure S14.** ^{13}C-NMR Spectrum of **2** in acetone-d_6-D_2O (125 MHz)
S17 **Figure S15.** ^{1}H-^{1}H-COSY Spectrum of **2** in acetone-d_6-D_2O
S18 **Figure S16.** HSQC Spectrum of **2** in acetone-d_6-D_2O
S19 **Figure S17.** HMBC Spectrum of **2** in acetone-d_6-D_2O
S20 **Figure S18.** NOE Spectrum of **2** in acetone-d_6-D_2O
S21 **Figure S19.** IR Spectrum of **3**
S22 **Figure S20.** ^{1}H-NMR Spectrum of **3** in acetone-d_6-D_2O (500 MHz)
S23 **Figure S21.** ^{13}C-NMR Spectrum of **3** in acetone-d_6-D_2O (125 MHz)
S24 **Figure S22.** ^{1}H-^{1}H-COSY Spectrum of **3** in acetone-d_6-D_2O
S25 **Figure S23.** HSQC Spectrum of **3** in acetone-d_6-D_2O
S26 **Figure S24.** HMBC Spectrum of **3** in acetone-d_6-D_2O
S27 **Figure S25.** NOE Spectrum of **3** in acetone-d_6-D_2O
S28 **Figure S26.** IR Spectrum of **7**
S29 **Figure S27.** ^{1}H-NMR Spectrum of **7** in acetone-d_6-D_2O (500 MHz)

S30 **Figure S28.** ^{13}C-NMR Spectrum of **7** in acetone-d_6-D_2O (125 MHz)

S31 **Figure S29.** ^{1}H-^{1}H-COSY Spectrum of **7** in acetone-d_6-D_2O

S32 **Figure S30.** HSQC Spectrum of **7** in acetone-d_6-D_2O

S33 **Figure S31.** HMBC Spectrum of **7** in acetone-d_6-D_2O

S34 **Figure S32.** NOE Spectrum of **7** in acetone-d_6-D_2O

S35 **Figure S33.** IR Spectrum of **13**

S36 **Figure S34.** ^{1}H-NMR Spectrum of **13** in CD_3OD (500 MHz)

S37 **Figure S35.** ^{13}C-NMR Spectrum of **13** in CD_3OD (125 MHz)

S38 **Figure S36.** ^{1}H-^{1}H-COSY Spectrum of **13** in CD_3OD

S39 **Figure S37.** HSQC Spectrum of **13** in CD_3OD

S40 **Figure S38.** HMBC Spectrum of **13** in CD_3OD

S41 **Figure S39.** NOE Spectrum of **13** in CD_3OD

S42 **Figure S40.** IR Spectrum of **14**

S43 **Figure S41.** ^{1}H-NMR Spectrum of **14** in acetone-d_6-D_2O (500 MHz)

S44 **Figure S42.** ^{13}C-NMR Spectrum of **14** in acetone-d_6-D_2O (125 MHz)

S45 **Figure S43.** ^{1}H-^{1}H-COSY Spectrum of **14** in acetone-d_6-D_2O

S46 **Figure S44.** HSQC Spectrum of **14** in acetone-d_6-D_2O

S47 **Figure S45.** HMBC Spectrum of **14** in acetone-d_6-D_2O

S48 **Figure S46.** NOE Spectrum of **14** in acetone-d_6-D_2O

S49 **Figure S47.** IR Spectrum of **16**

S50 **Figure S48.** ^{1}H-NMR Spectrum of **16** in acetone-d_6-D_2O (500 MHz)

S51 **Figure S49.** ^{13}C-NMR Spectrum of **16** in acetone-d_6-D_2O (125 MHz)

S52 **Figure S50.** ^{1}H-^{1}H-COSY Spectrum of **16** in acetone-d_6-D_2O

S53 **Figure S51.** HSQC Spectrum of **16** in acetone-d_6-D_2O

S54 **Figure S52.** HMBC Spectrum of **16** in acetone-d_6-D_2O

S55 **Figure S53.** NOE Spectrum of **16** in acetone-d_6-D_2O

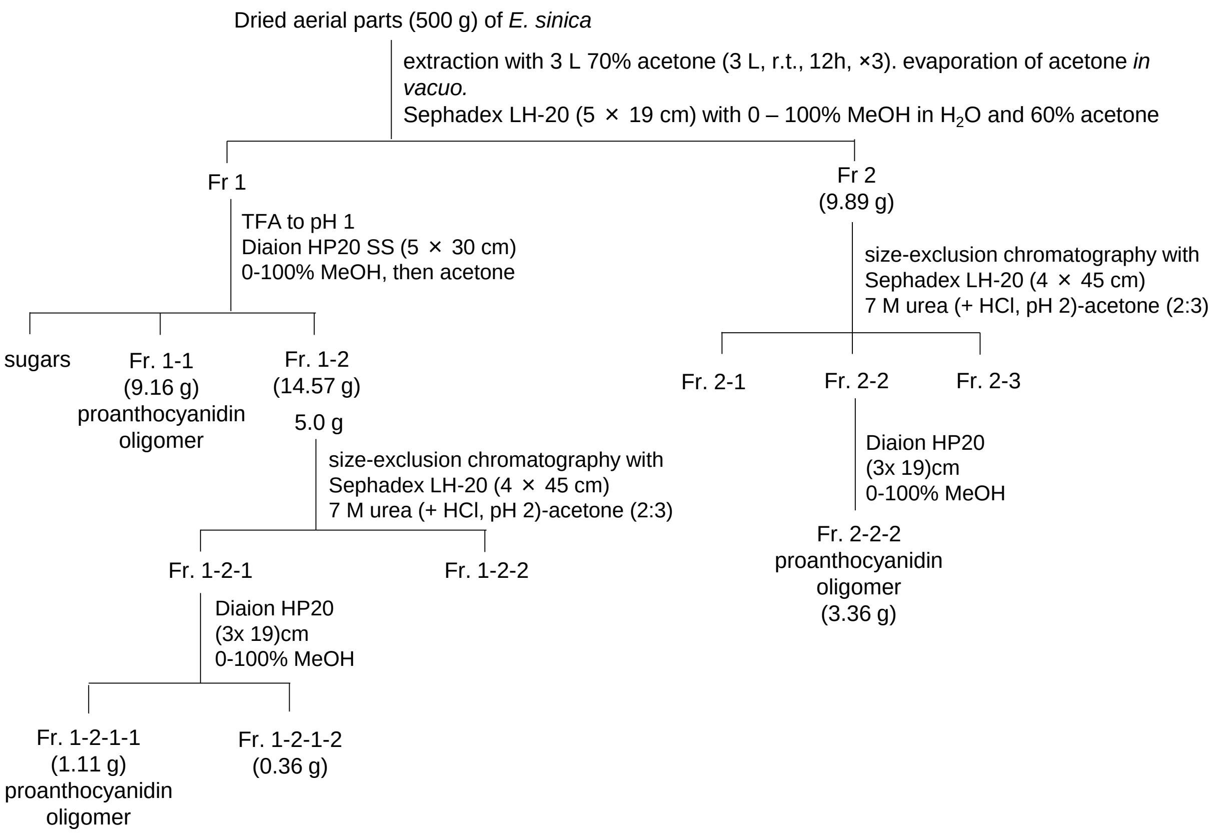

Figure S1. Fractionation of Proanthocyanidin Oligomers of *E. sinica*

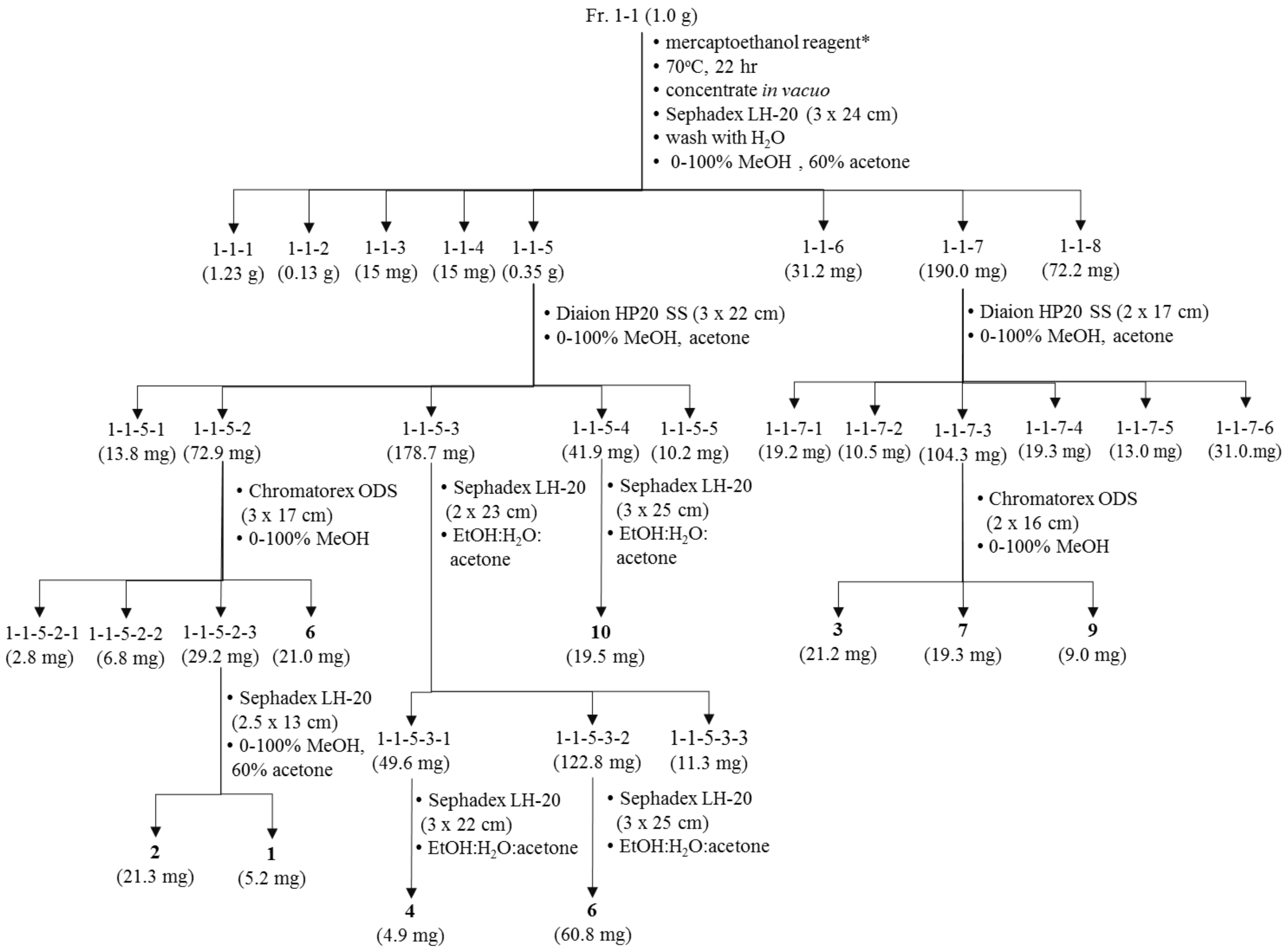

***mercaptoethanol reagent:** 60% EtOH (200 mL) + mercaptoethanol (10 mL) + conc HCl (0.5 mL)

Figure S2. Isolation of Thiol Degradation Products of *E. sinica* Fr. 1-1

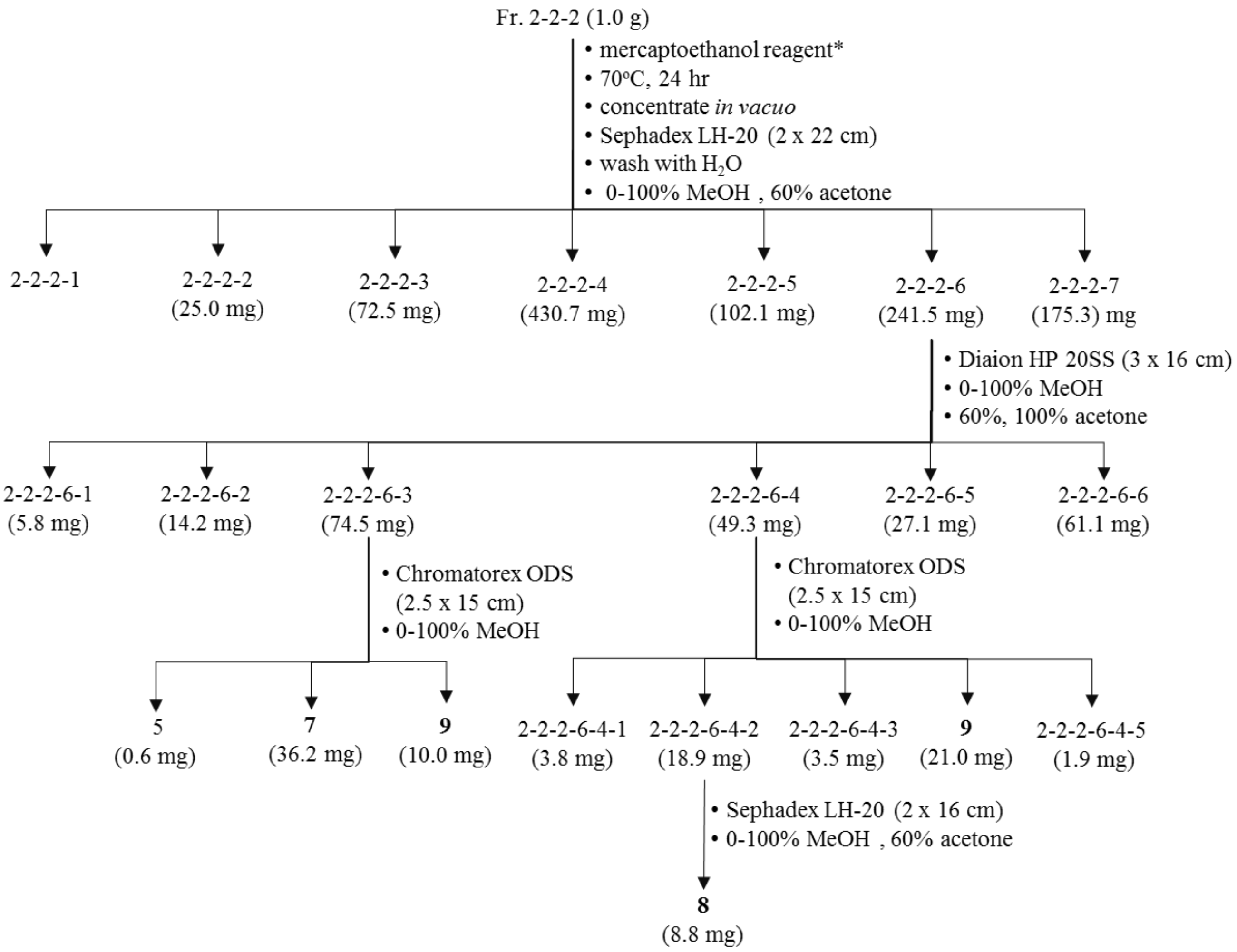

***mercaptoethanol reagent:** 60% EtOH (200 mL) + mercaptoethanol (10 mL) + conc HCl (0.5 mL)

Figure S3. Isolation of Thiol Degradation Products of *E. sinica* Fr. 2-2-2

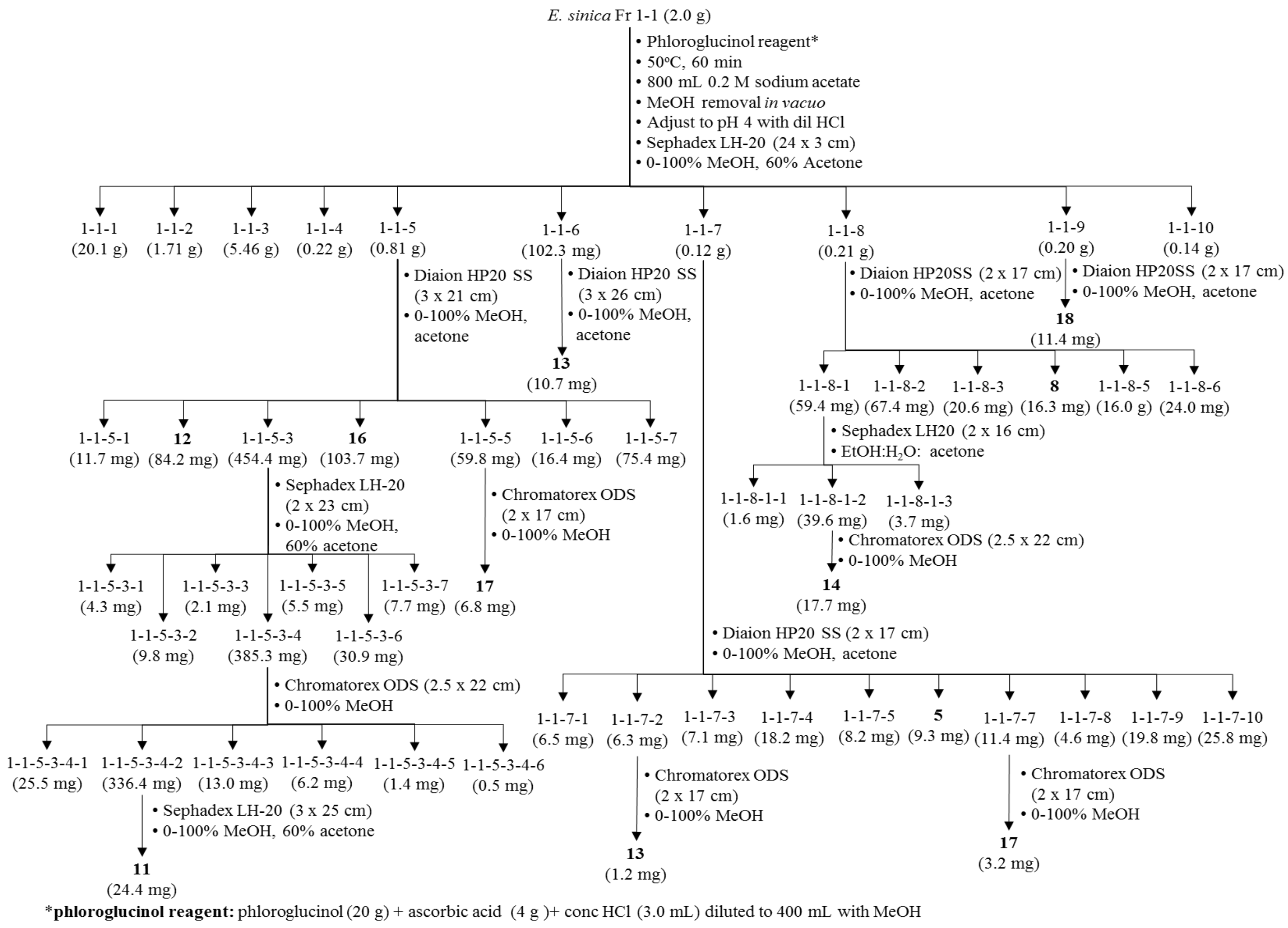

Figure S4. Isolation of Phloroglucinol Degradation Products of *E. sinica* Fr. 1-1

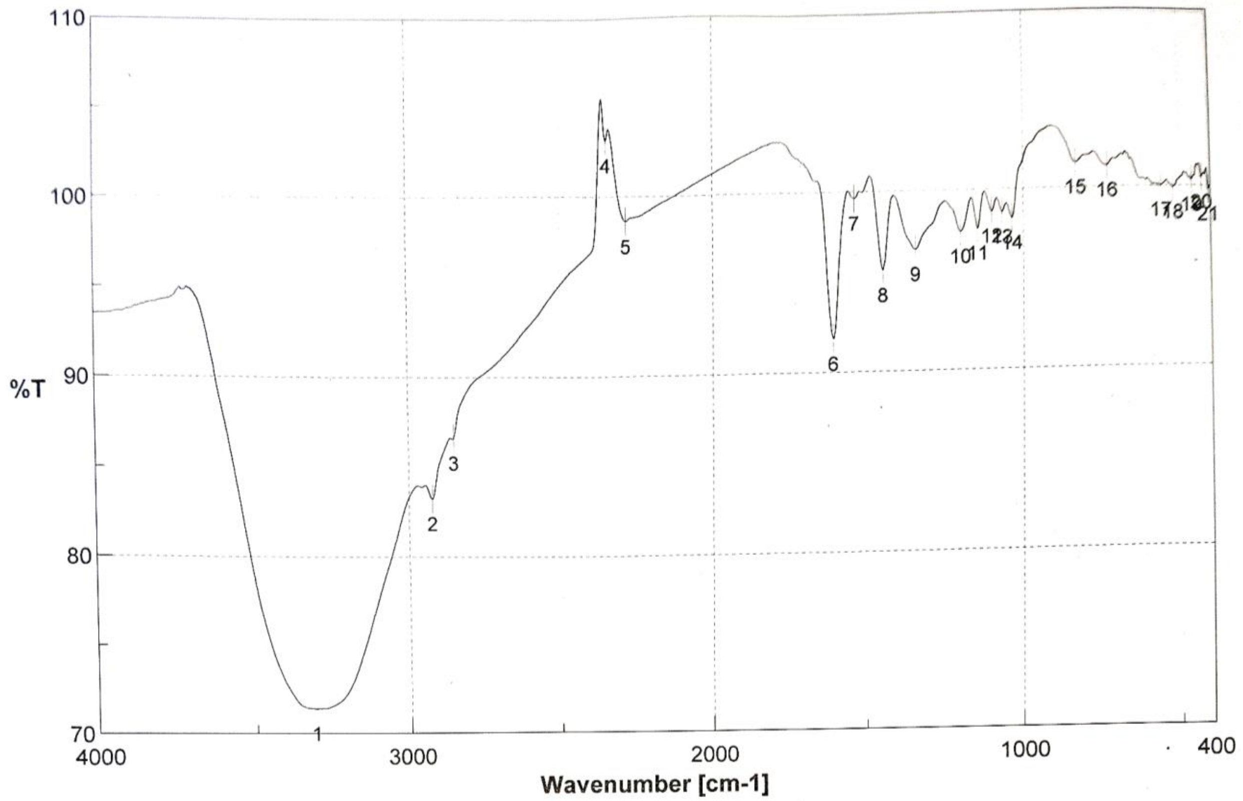

積算回数	Auto (38)
分解	4 cm-1
ゼロフィリング	ON
アポダイゼーション	Cosine
ゲイン	Auto (2)
スキャンスピード	Auto (2 mm/sec)
測定日時	2017/02/20 19:00
更新日時	2017/02/20 19:04
測定者	
ファイル名	E sinica Thiol Degradation Fr 1-1-5-2-3-2 edited
サンプル名	
コメント	

No.	cm-1	%T	No.	cm-1	%T	No.	cm-1	%T
1	3311.18	71.3939	2	2923.56	83.2425	3	2852.2	86.6283
4	2345.98	103.149	5	2282.34	98.6175	6	1609.31	91.8572
7	1541.81	99.6249	8	1449.24	95.5995	9	1345.11	96.7273
10	1200.47	97.6668	11	1145.51	97.8066	12	1100.19	98.7539
13	1067.41	98.6998	14	1035.59	98.3641	15	832.133	101.398
16	730.889	101.199	17	559.255	100.051	18	520.686	99.9092
19	460.904	100.317	20	428.12	100.447	21	406.907	99.7654

Figure S5. IR Spectrum of **1**

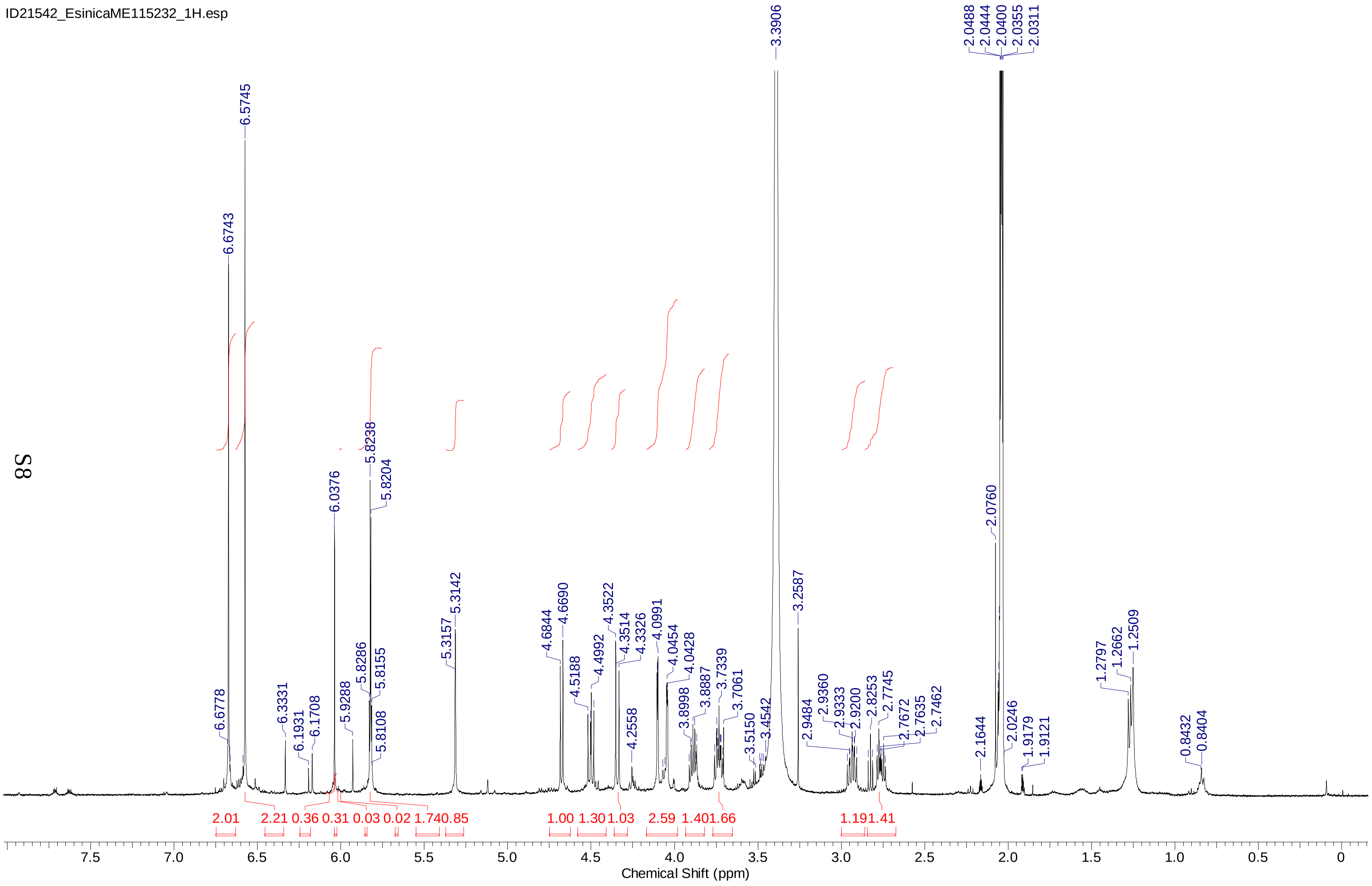

Figure S6. ^{1}H-NMR Spectrum of **1** in acetone-d_6-D_2O (500 MHz)

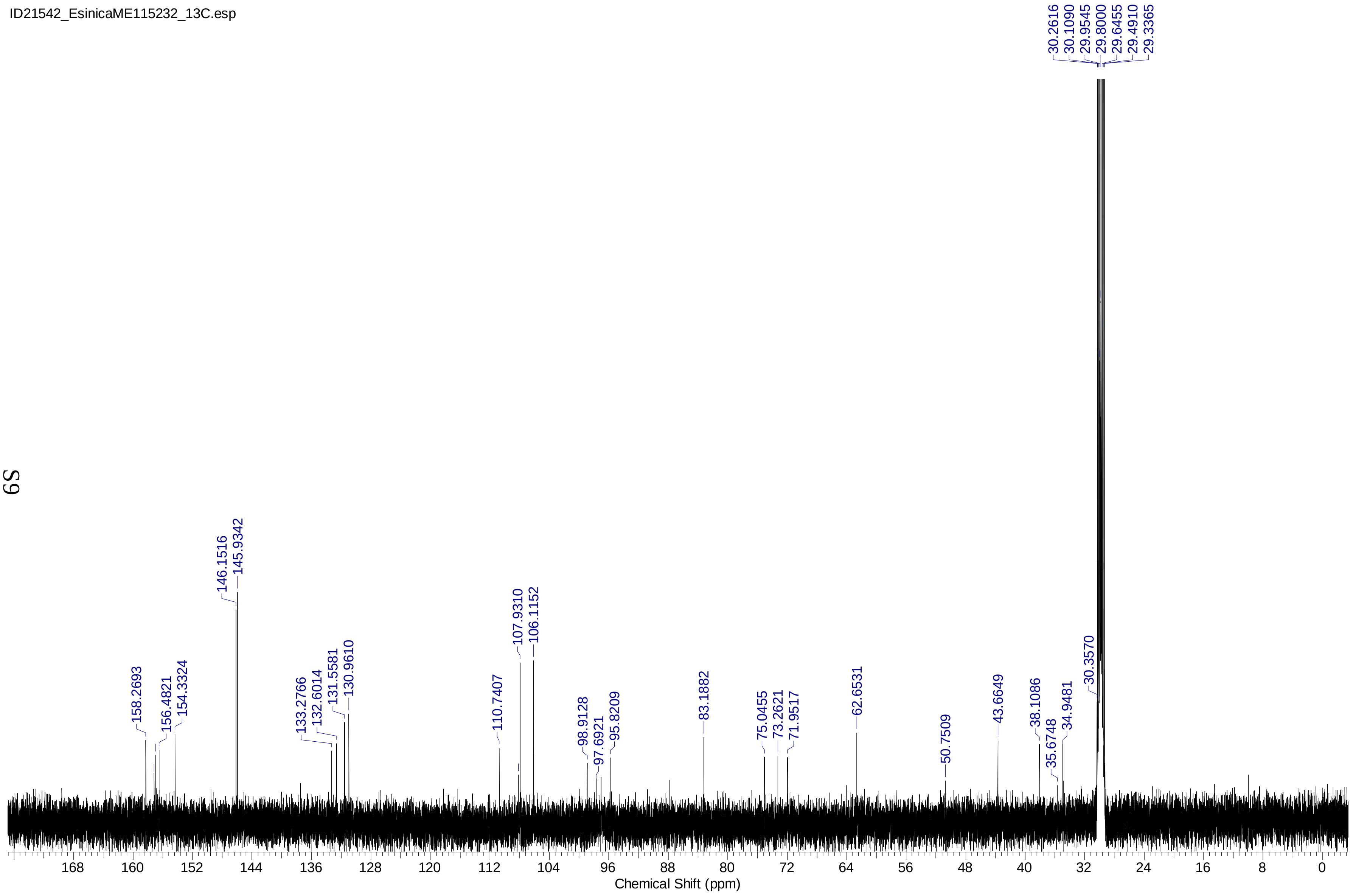

Figure S7. ^{13}C-NMR Spectrum of **1** in acetone-d_6-D_2O (125 MHz)

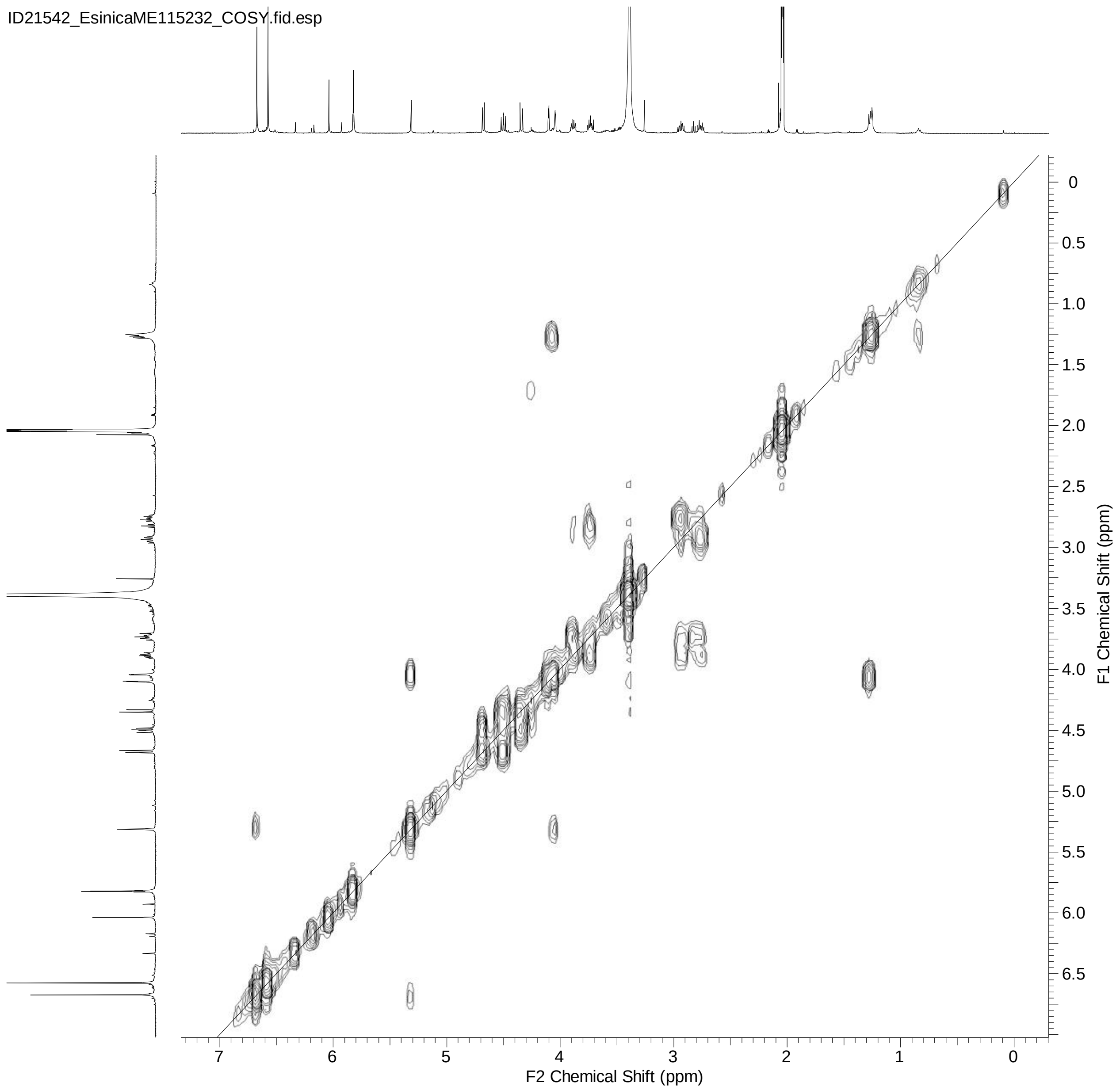

Figure S8. ^{1}H-^{1}H-COSY Spectrum of **1** in acetone-d_6-D_2O

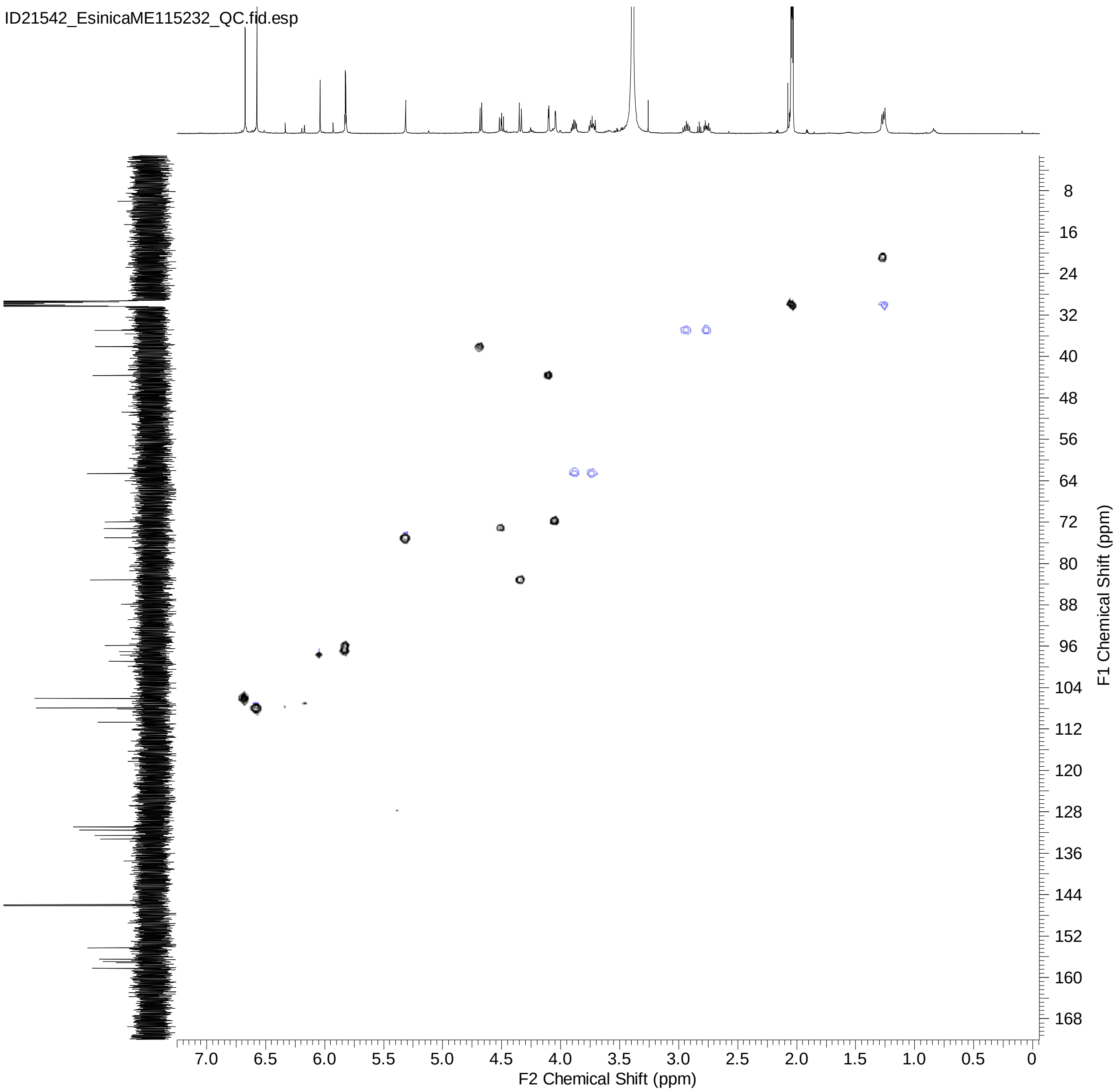

Figure S9. HSQC Spectrum of **1** in acetone-d_6-D_2O

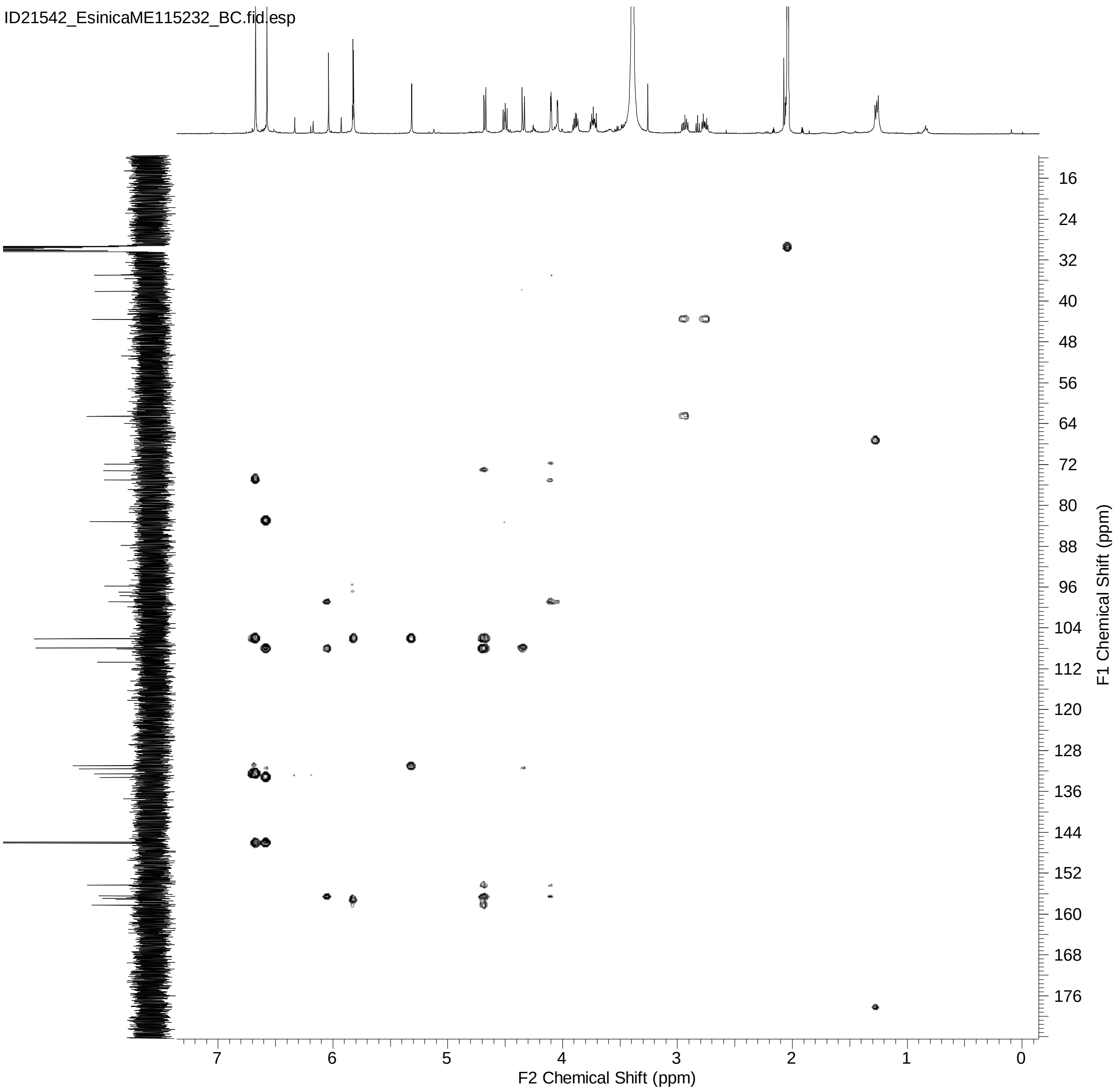

Figure S10. HMBC Spectrum of **1** in acetone-d_6-D_2O

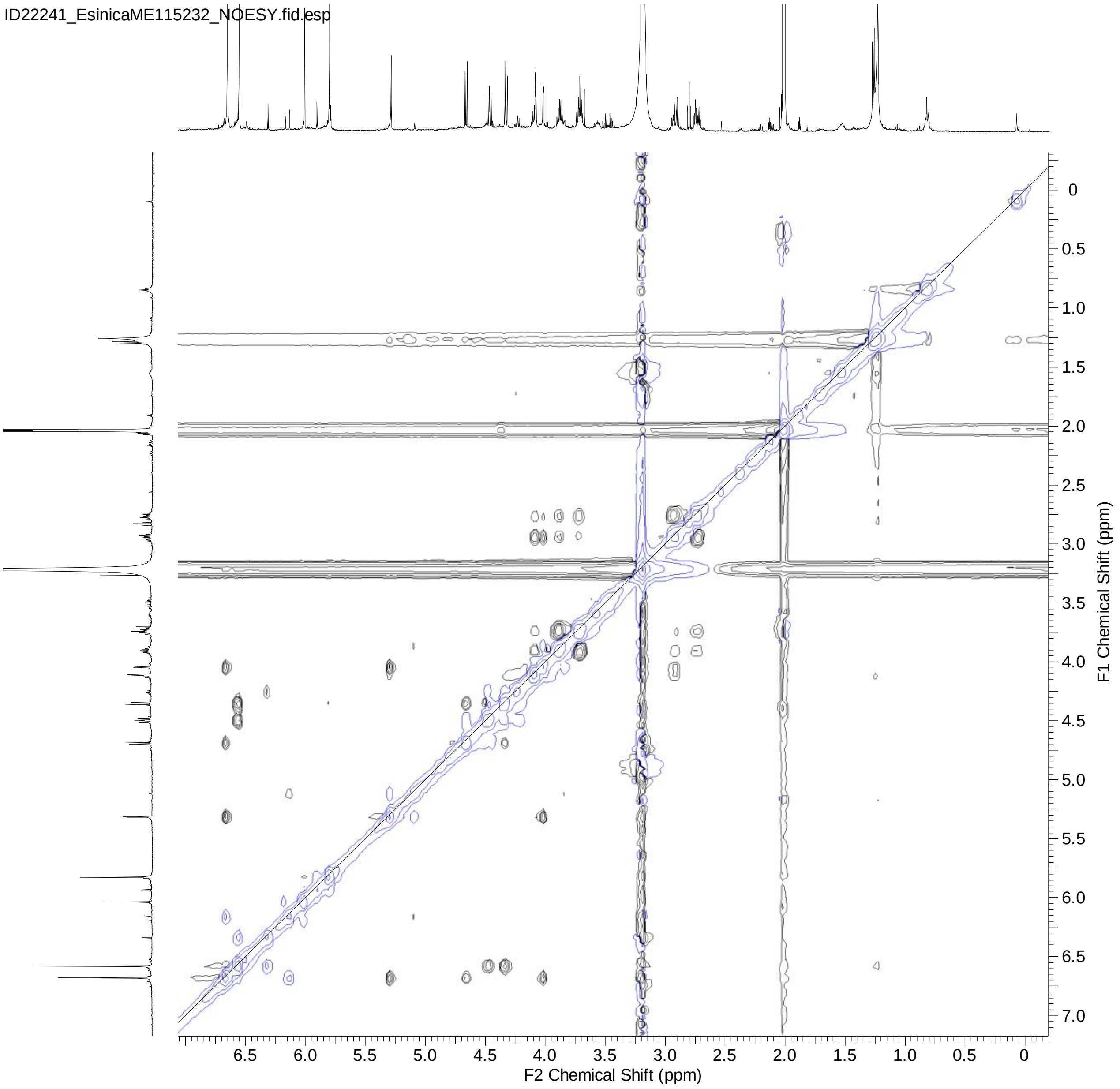

Figure S11. NOE Spectrum of **1** in acetone-d_6-D_2O

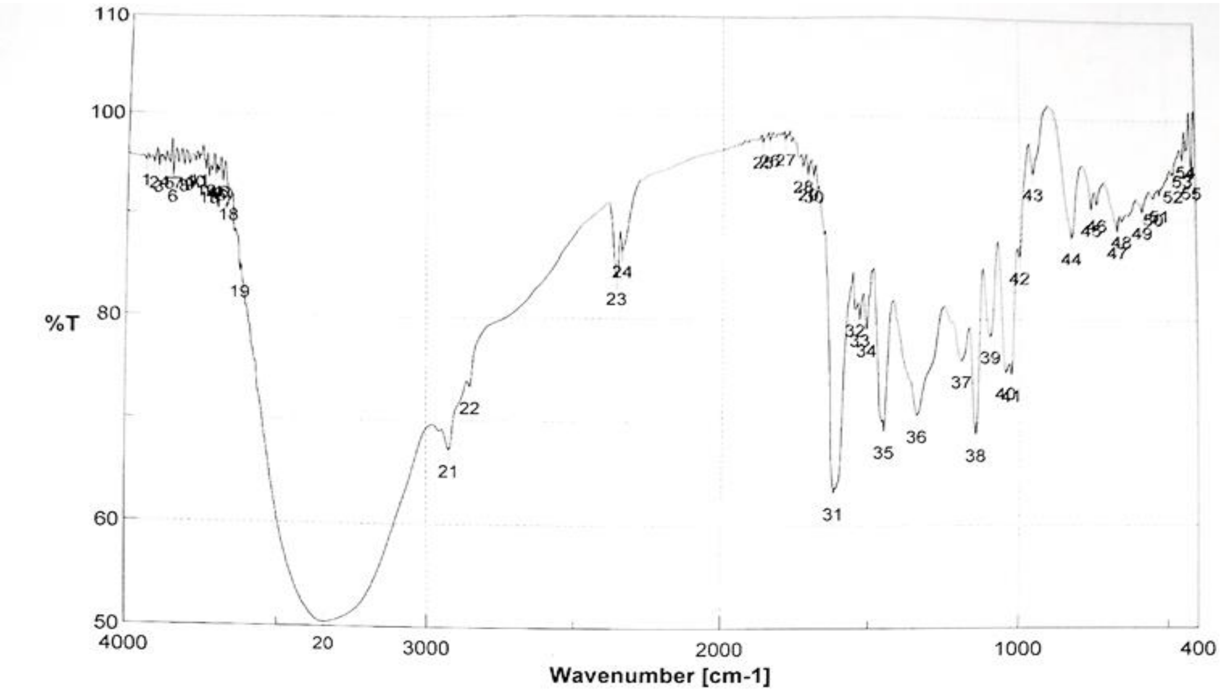

積算回数	Auto (43)
分解	4 cm-1
ゼロフィリング	ON
アポダイゼーション	Cosine
ゲイン	Auto (4)
スキャンスピード	Auto (2 mm/sec)
測定日時	2017/02/03 15:49
更新日時	2017/02/03 16:02
測定者	
ファイル名	Memory#6
サンプル名	E. sinica Thiol Degradation Fr 115231
コメント	

No.	cm-1	%T	No.	cm-1	%T	No.	cm-1	%T
1	3939.86	95.2343	2	3913.82	94.9744	3	3898.4	94.5905
4	3879.11	95.0403	5	3861.76	94.9417	6	3850.18	93.6571
7	3833.79	94.9751	8	3813.54	94.7316	9	3796.19	94.8532
10	3776.9	95.1736	11	3765.33	95.2869	12	3742.19	94.2927
13	3731.58	93.6158	14	3720.01	94.257	15	3707.48	93.8487
16	3698.8	94.1795	17	3685.3	93.2361	18	3666.02	91.9669
19	3625.52	84.4034	20	3344.93	50.4179	21	2923.56	67.2177
22	2852.2	73.3251	23	2358.52	83.9892	24	2339.23	86.6457
25	1865.79	97.4619	26	1841.69	97.6548	27	1789.62	97.7342
28	1729.83	95.0218	29	1713.44	94.26	30	1694.16	94.0552
31	1621.84	63.2072	32	1554.34	80.9834	33	1537.95	80.0316
34	1515.78	79.0392	35	1455.03	69.1604	36	1345.11	70.6891
37	1196.61	75.9866	38	1147.44	68.8515	39	1099.23	78.3846
40	1047.16	74.861	41	1027.87	74.6438	42	1000.87	86.1589
43	953.627	94.4508	44	824.42	88.1325	45	756.923	90.966
46	737.639	91.4568	47	668.214	88.8032	48	653.75	89.8471
49	583.361	90.7706	50	545.756	92.1339	51	524.543	92.466
52	477.296	94.477	53	444.512	96.0301	54	431.977	96.973
55	414.62	94.8566						

Figure S12. IR Spectrum of **2**

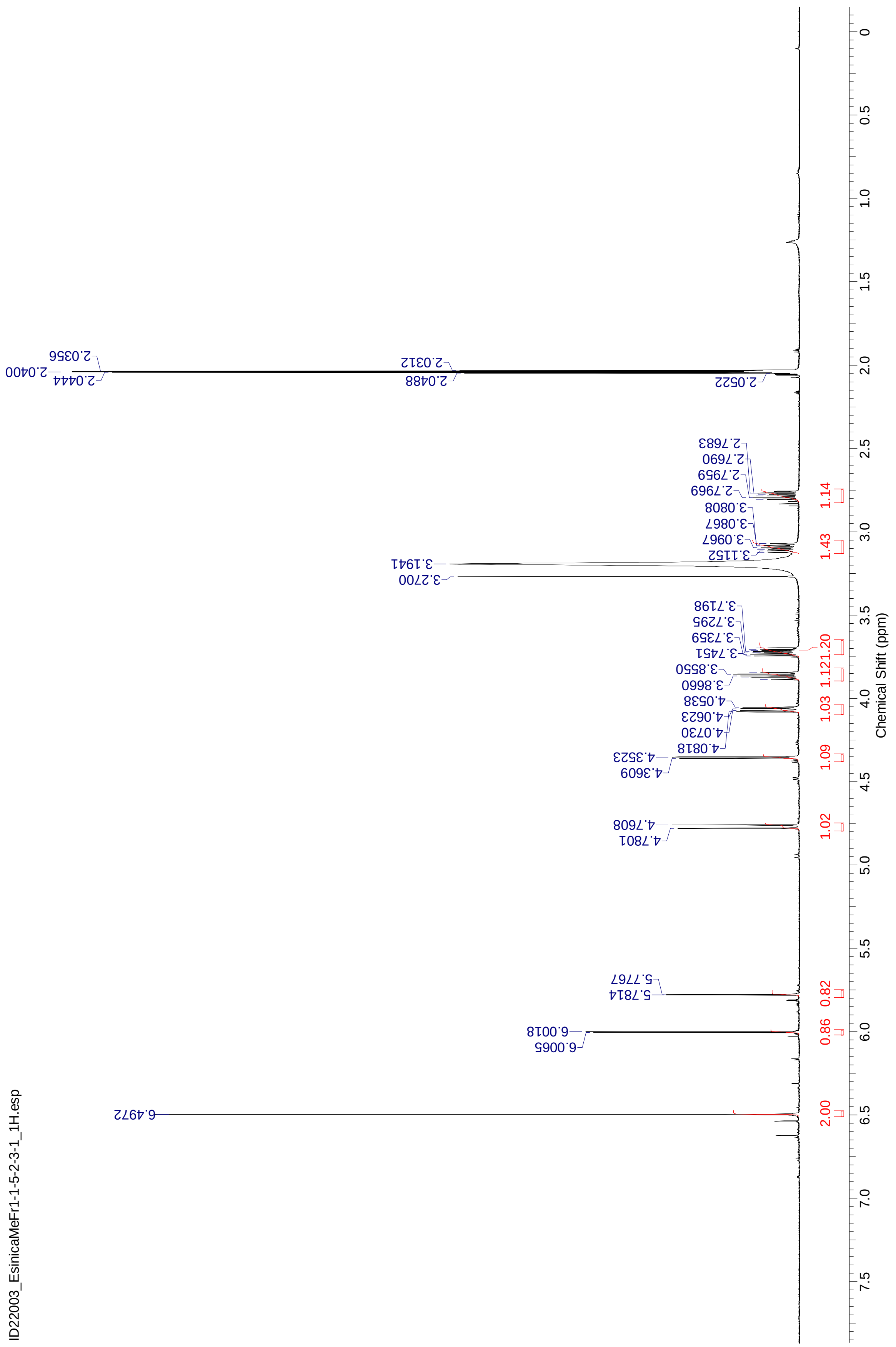

Figure S13. ^{1}H-NMR Spectrum of **2** in acetone-d_6-D_2O (500 MHz)

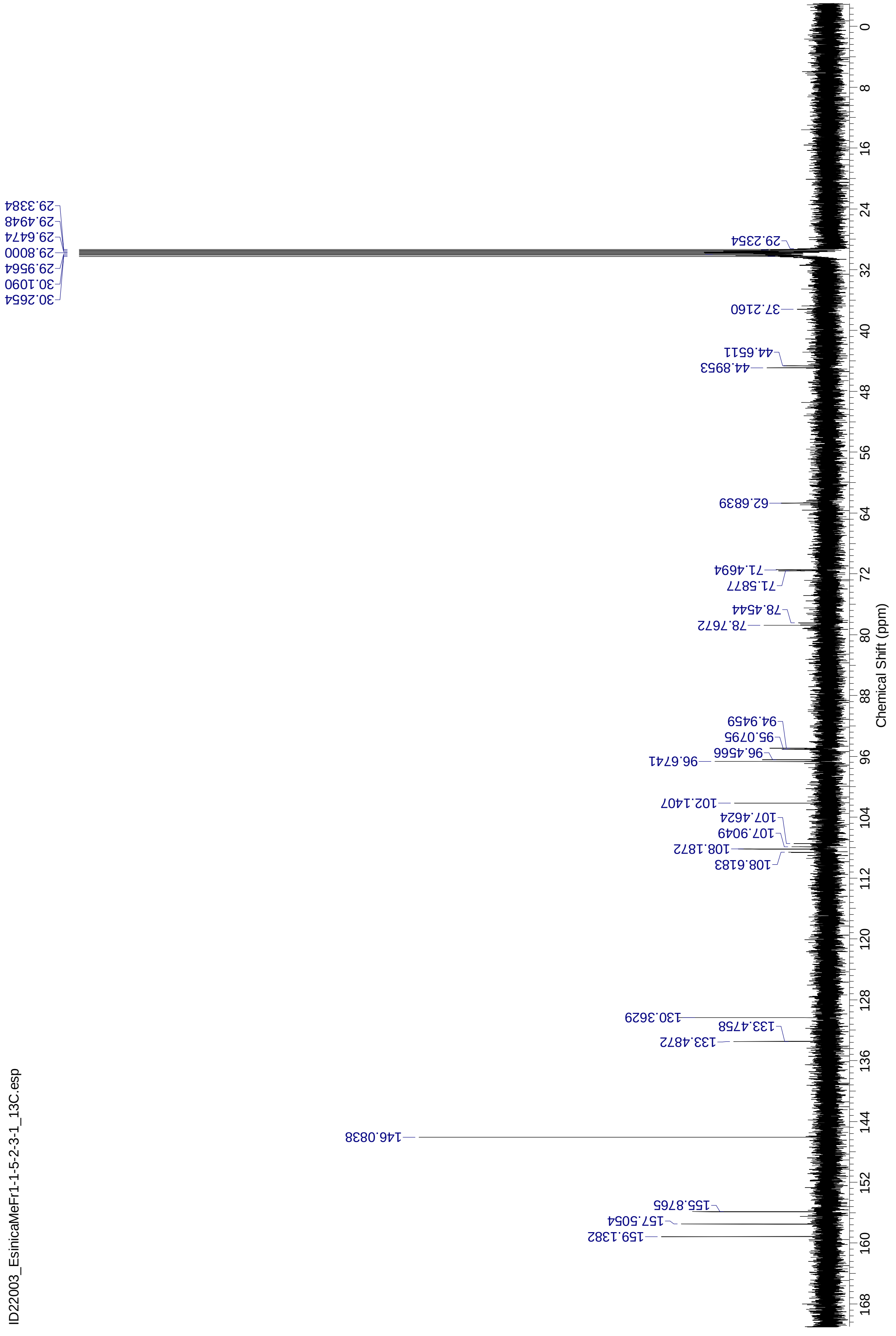

Figure S14. ^{13}C-NMR Spectrum of **2** in acetone-d_6-D_2O (125 MHz)

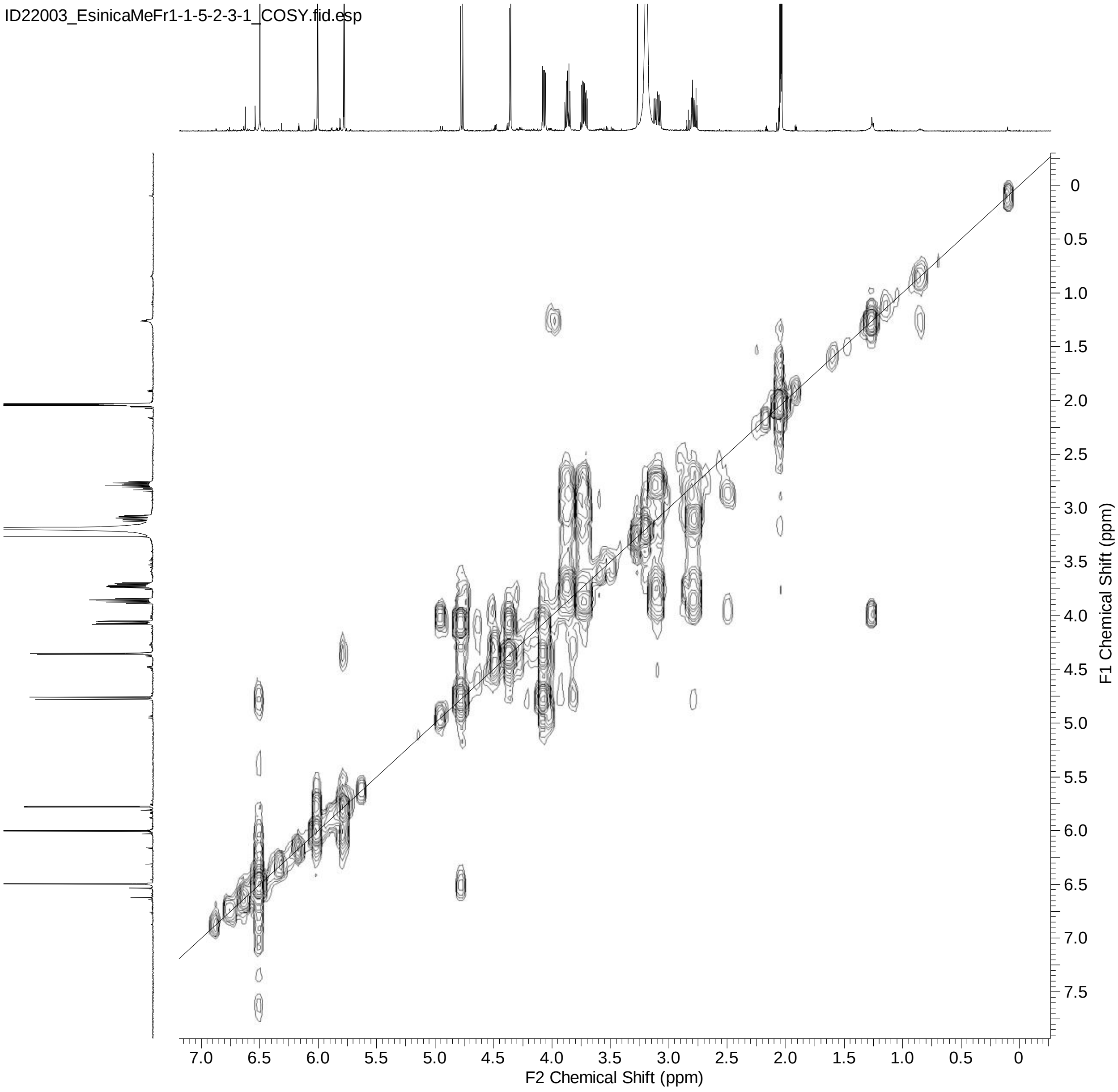

Figure S15. ^{1}H-^{1}H-COSY Spectrum of **2** in acetone-d_6-D_2O

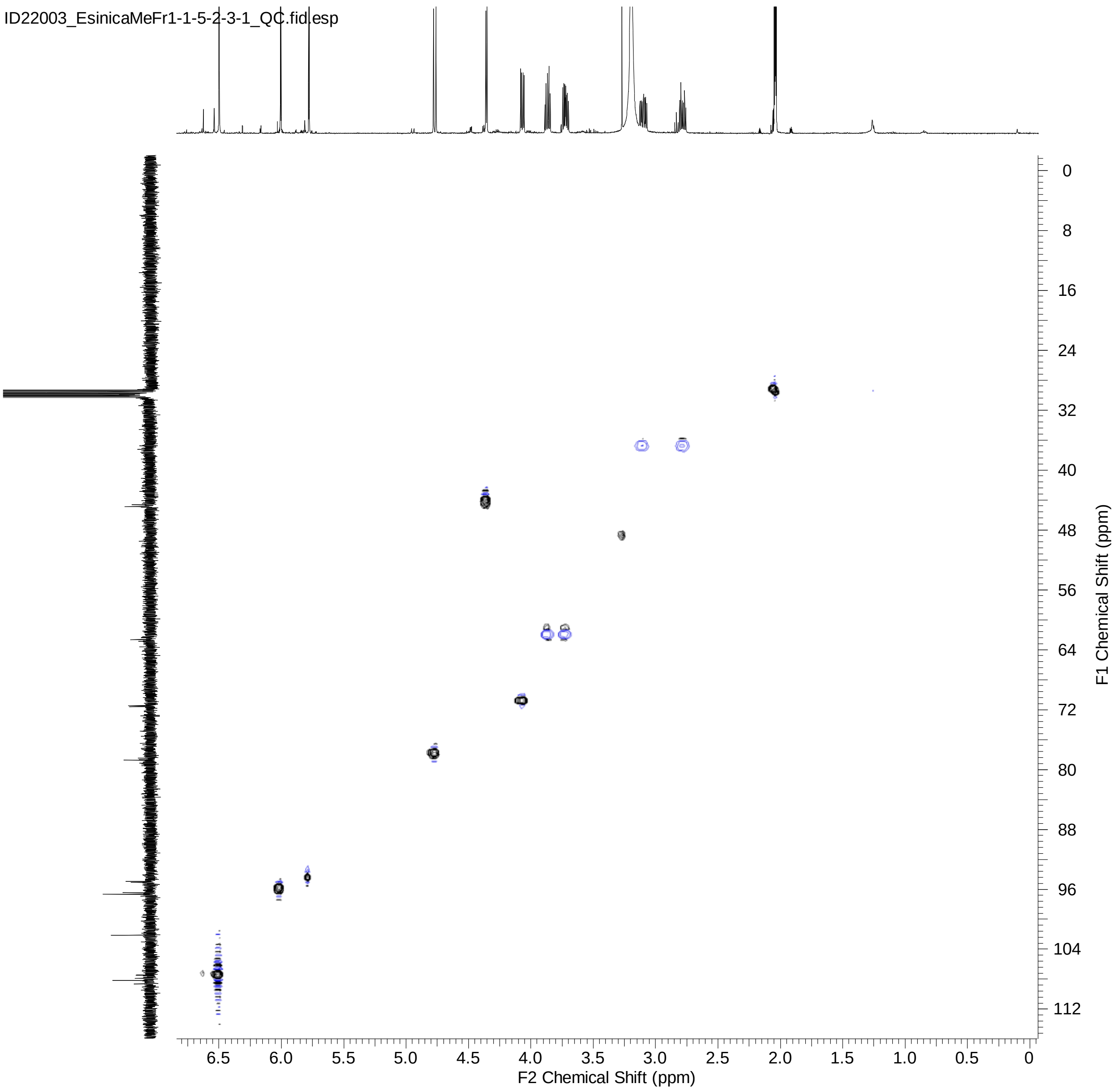

Figure S16. HSQC Spectrum of **2** in acetone-d_6-D_2O

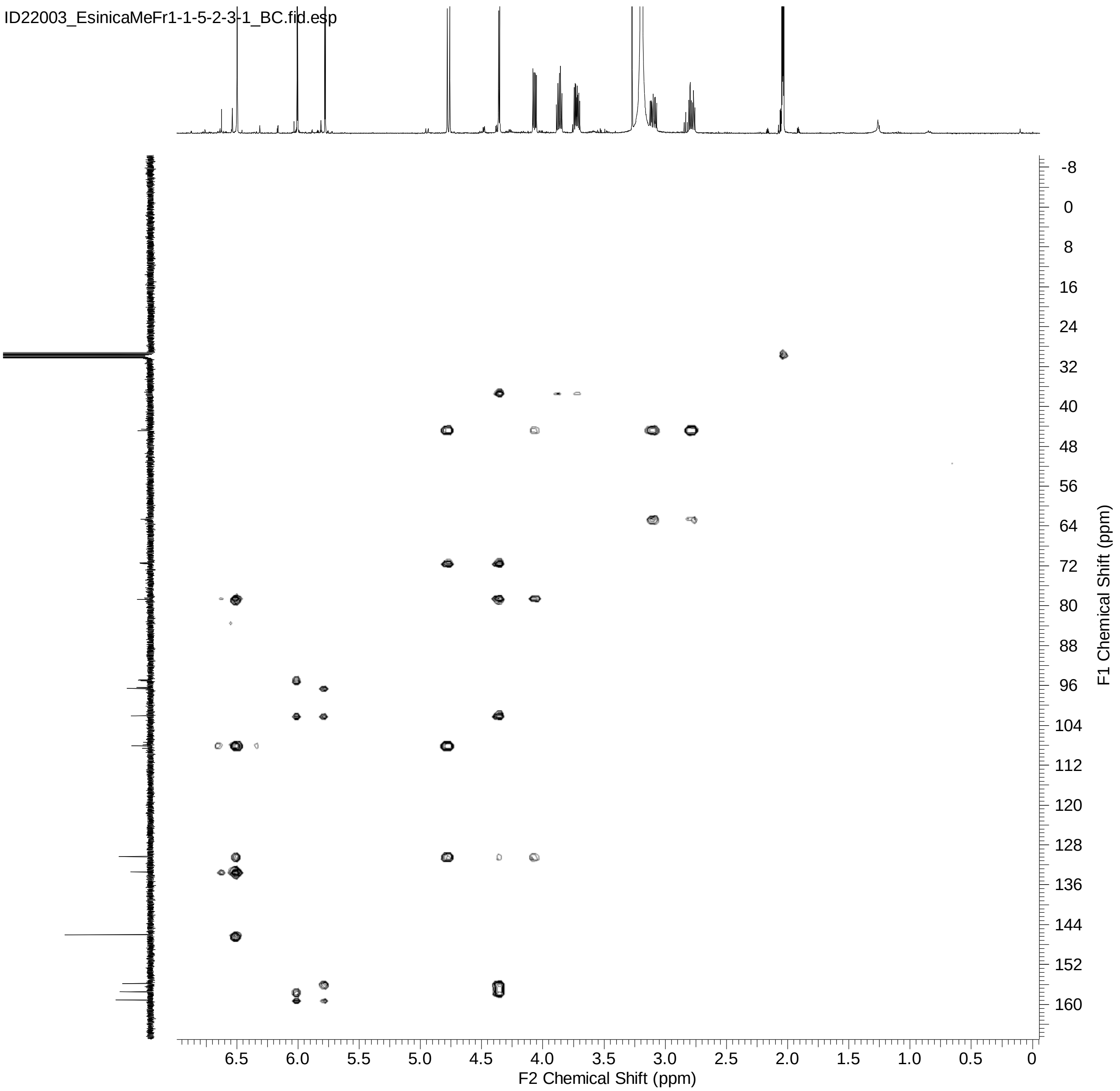

Figure S17. HMBC Spectrum of **2** in acetone-d_6-D_2O

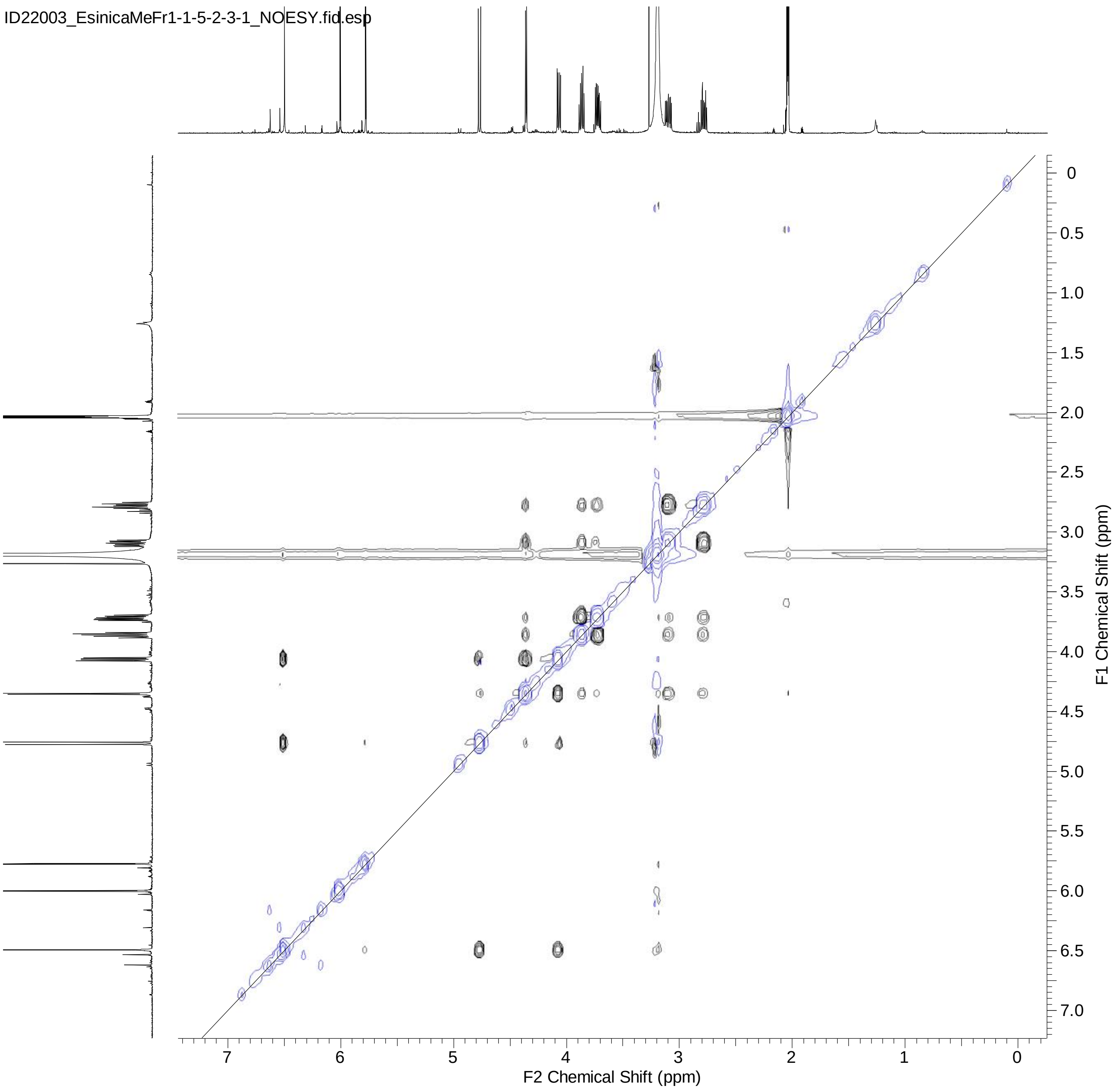

Figure S18. NOE Spectrum of **2** in acetone-d_6-D_2O

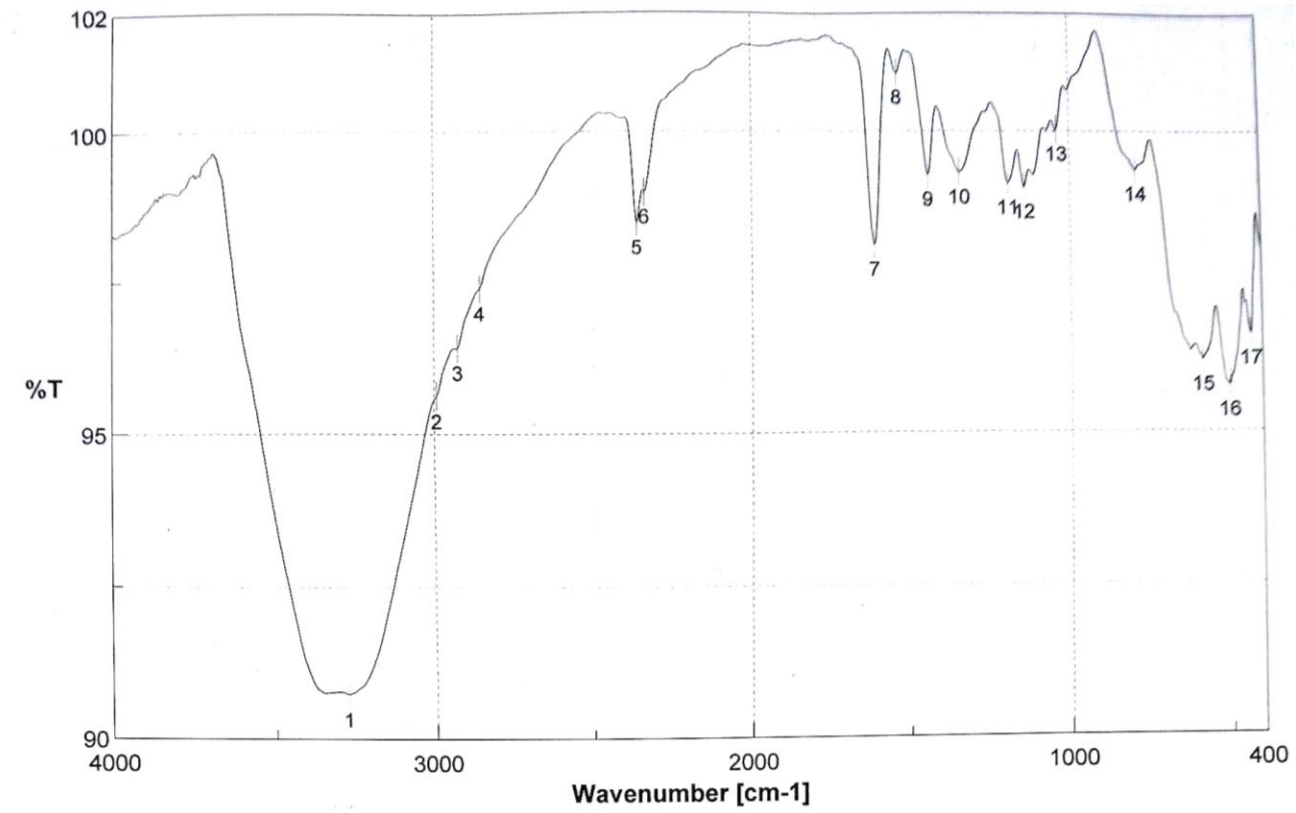

積算回数	Auto (34)
分解	4 cm-1
ゼロフィリング	ON
アポダイゼーション	Cosine
ゲイン	Auto (2)
スキャンスピード	Auto (2 mm/sec)
測定日時	2017/02/20 15:31
更新日時	2017/02/20 17:27
測定者	
ファイル名	E sinica Thiol Degradation Fr 1-1-7-3-2 edited
サンプル名	
コメント	

No.	cm-1	%T	No.	cm-1	%T	No.	cm-1	%T
1	3276.47	90.7472	2	2992.02	95.6274	3	2925.48	96.4176
4	2856.06	97.3959	5	2361.41	98.4879	6	2337.3	98.9935
7	1615.09	98.1047	8	1540.85	100.992	9	1445.39	99.2701
10	1348	99.31	11	1194.69	99.1351	12	1143.58	99.0633
13	1039.44	100.033	14	790.671	99.3621	15	586.254	96.1923
16	503.33	95.7705	17	435.834	96.6282			

Figure S19. IR Spectrum of **3**

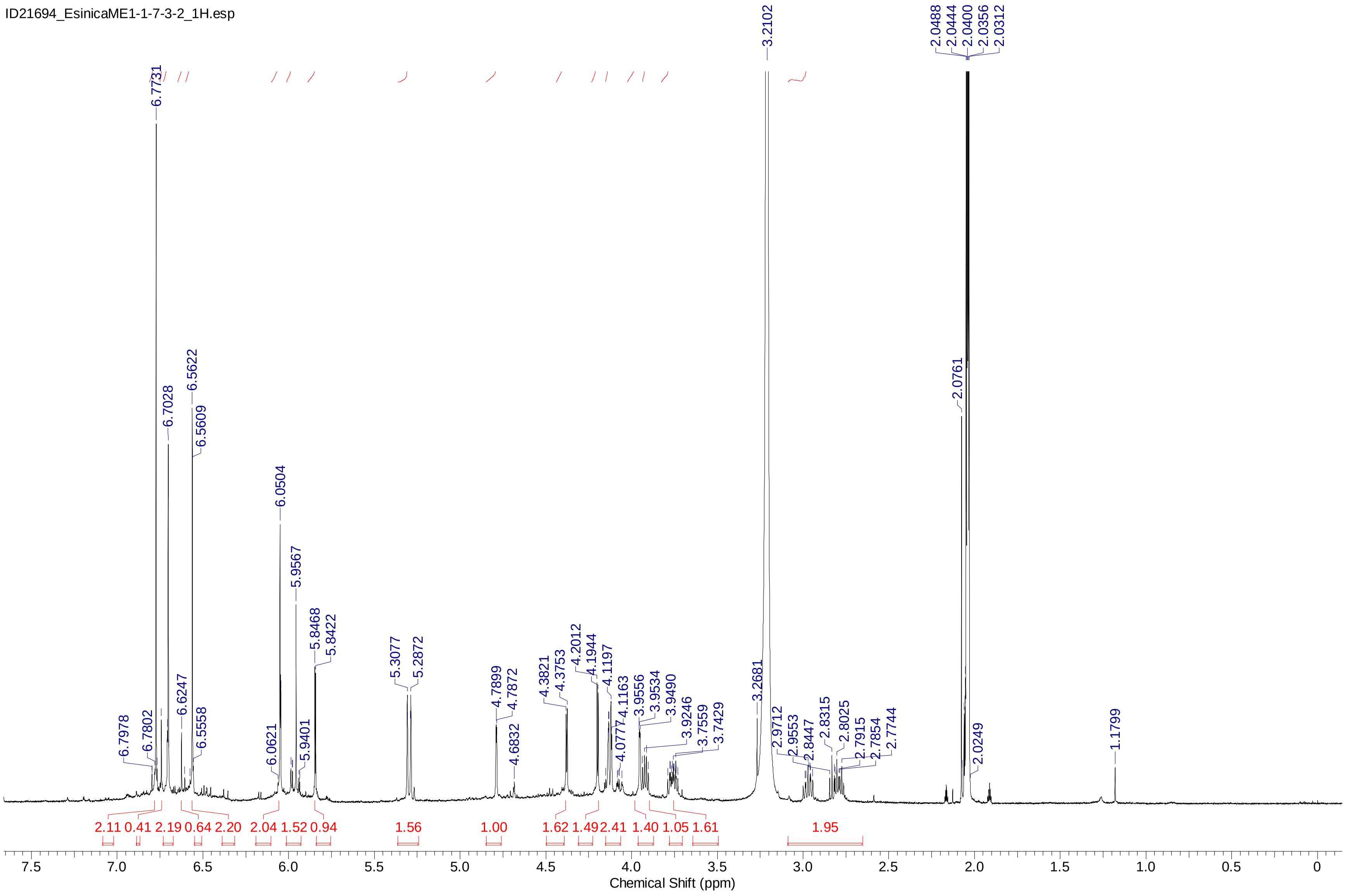

Figure S20. ^{1}H-NMR Spectrum of **3** in acetone-d_6-D_2O (500 MHz)

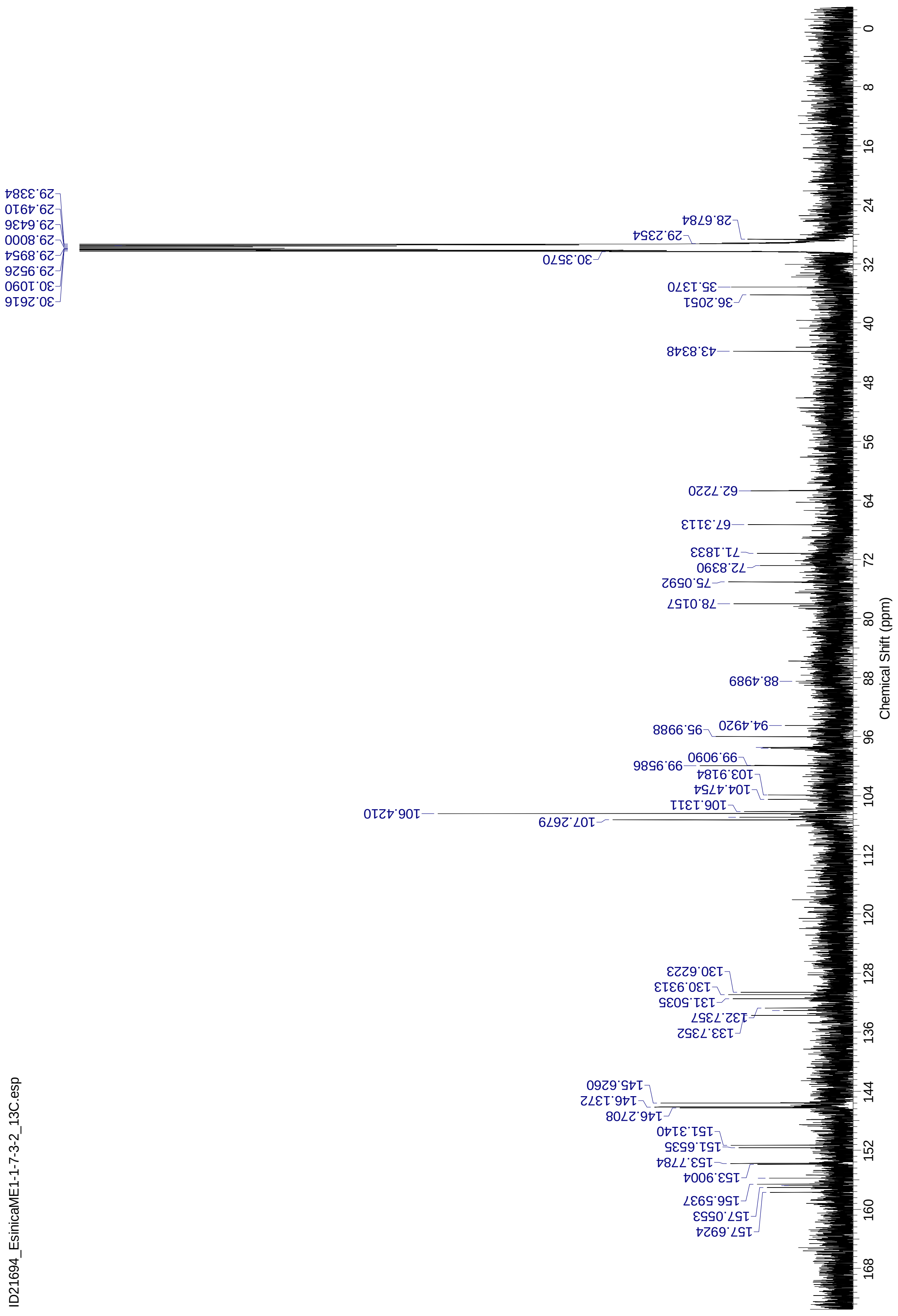

Figure S21. ^{13}C-NMR Spectrum of **3** in acetone-d_6-D_2O (125 MHz)

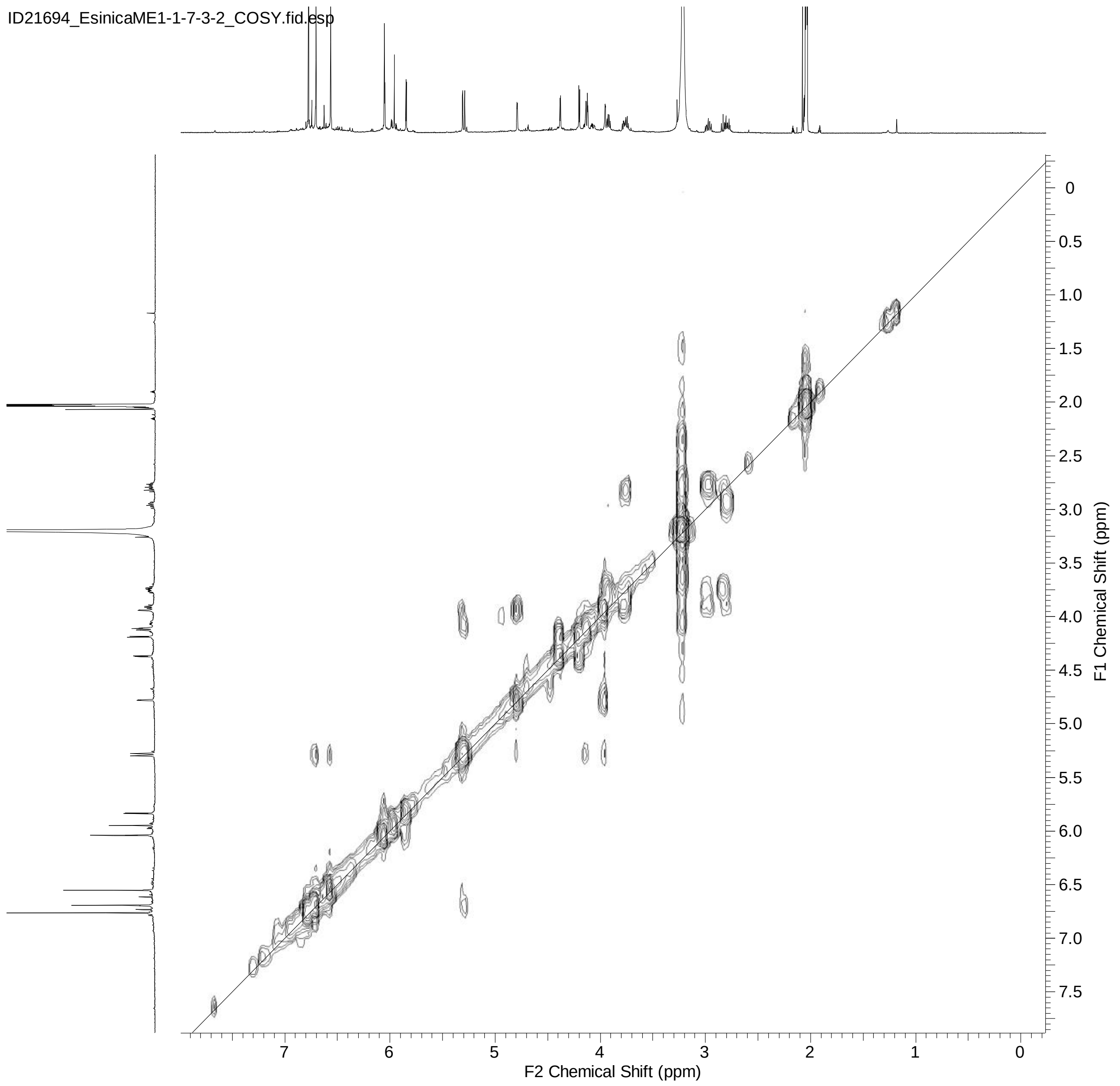

Figure S22. ^{1}H-^{1}H-COSY Spectrum of **3** in acetone-d_6-D_2O

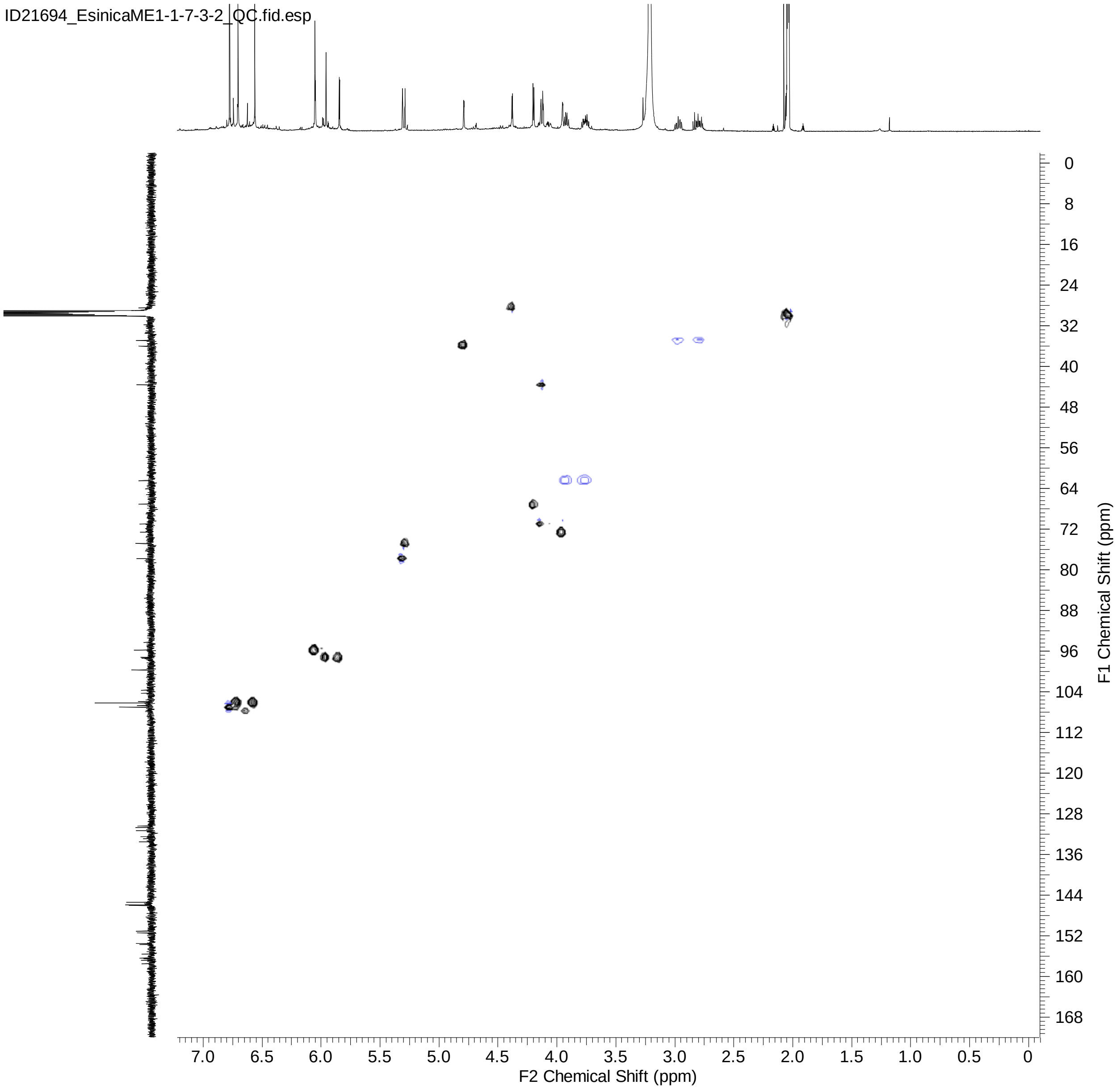

Figure S23. HSQC Spectrum of **3** in acetone-d_6-D_2O

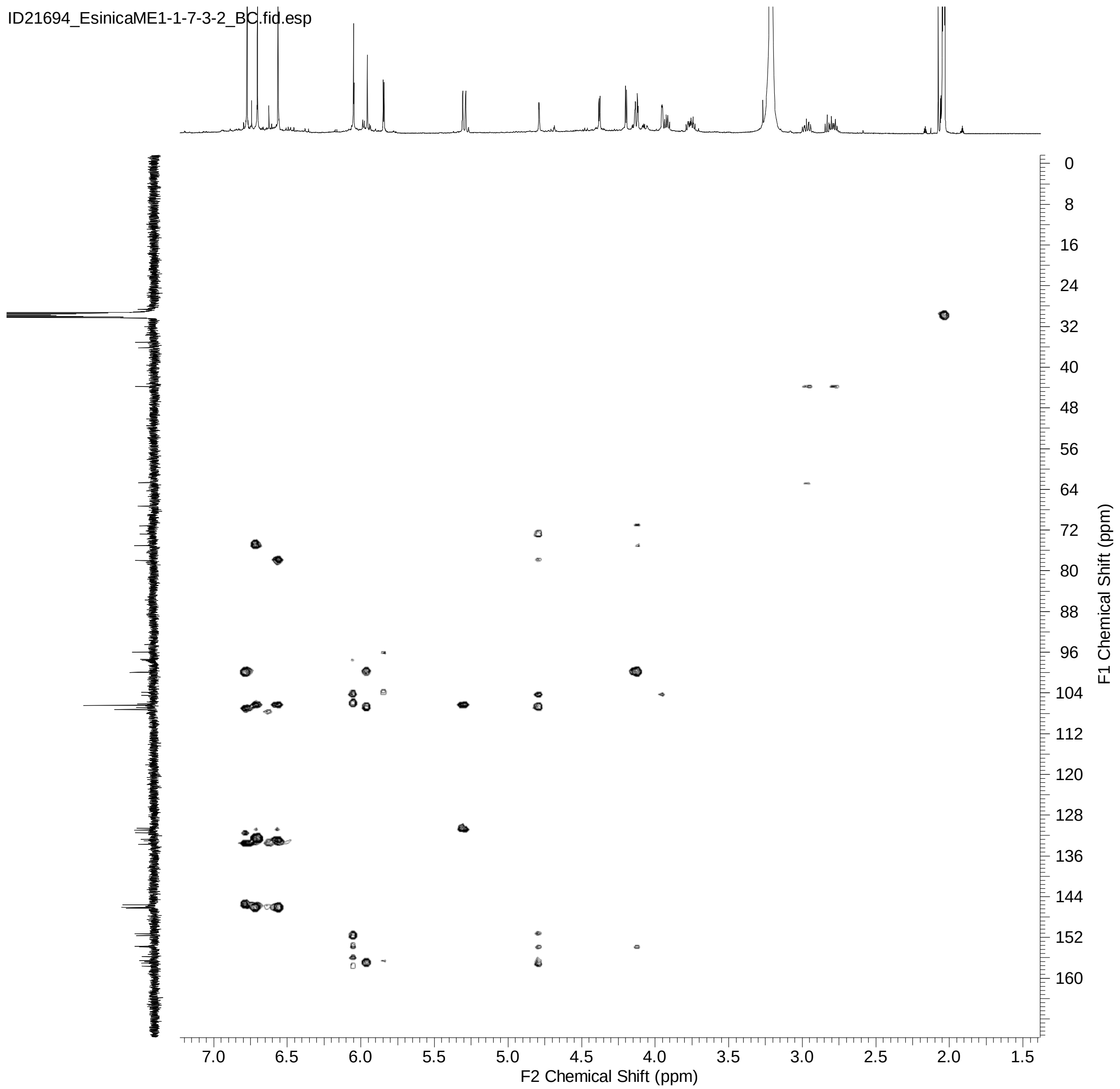

Figure S24. HMBC Spectrum of **3** in acetone-d_6-D_2O

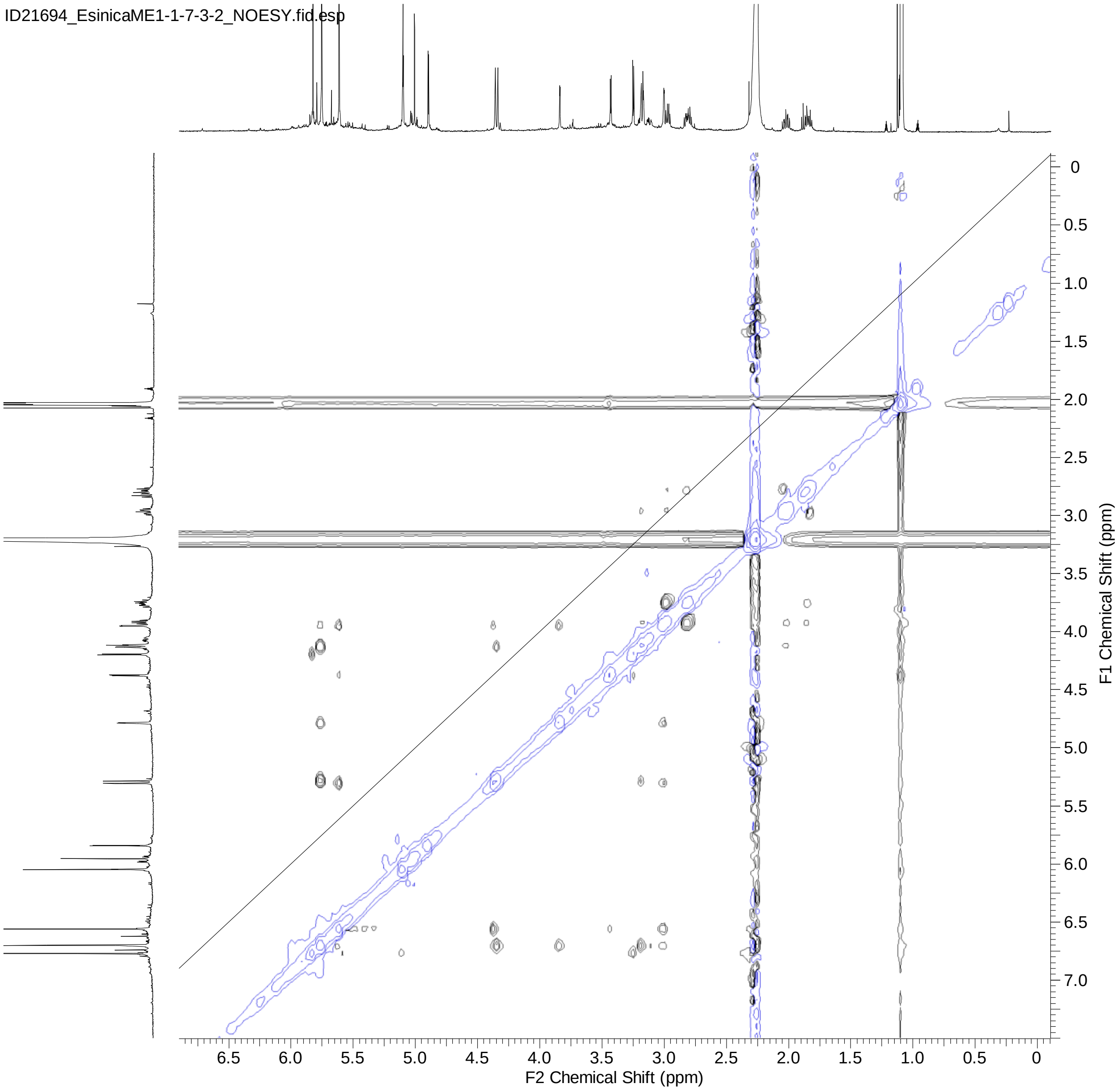

Figure S25. NOE Spectrum of **3** in acetone-d_6-D_2O

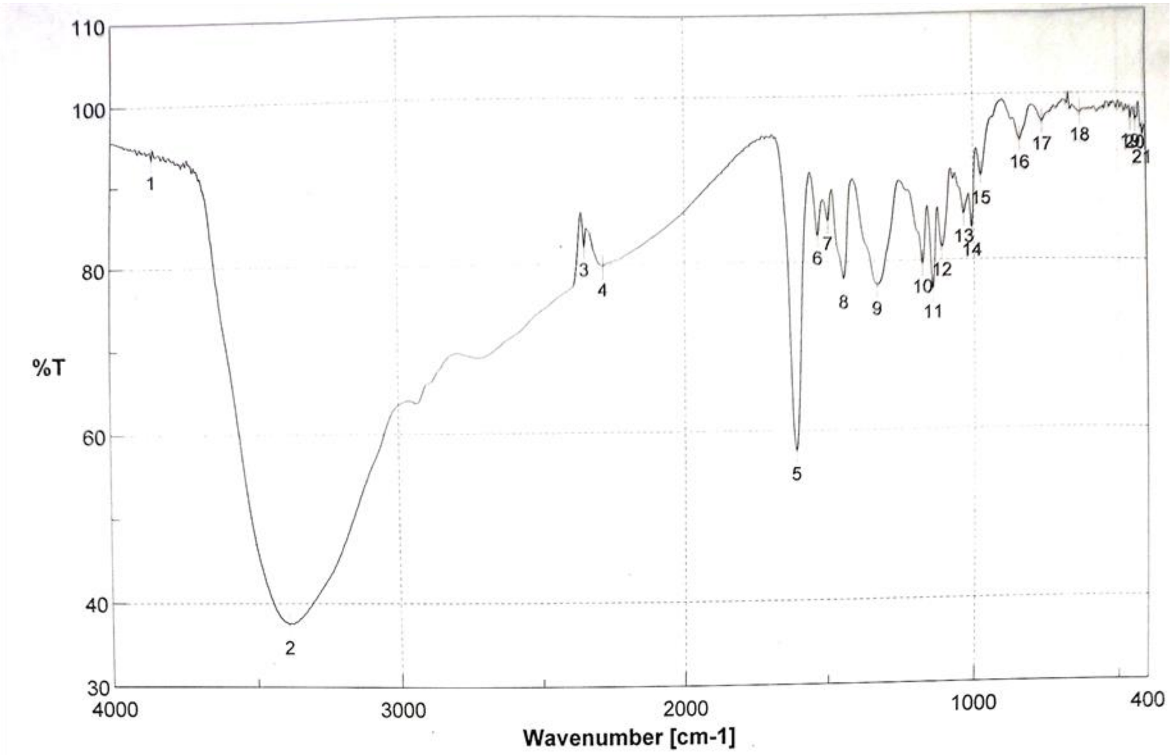

積算回数	Auto (43)
分解	4 cm-1
ゼロフィリング	ON
アポダイゼーション	Cosine
ゲイン	Auto (4)
スキャンスピード	Auto (2 mm/sec)
測定日時	2017/01/23 14:48
更新日時	2017/01/23 14:54
測定者	
ファイル名	Memory#5
サンプル名	E. sinica Thiol Degradation Fr 222634
コメント	

No.	cm-1	%T	No.	cm-1	%T	No.	cm-1	%T
1	3855.01	93.4735	2	3389.28	37.5189	3	2347.91	82.1385
4	2283.3	79.7479	5	1612.2	57.6667	6	1536.99	83.2304
7	1502.28	85.007	8	1447.31	77.9686	9	1332.57	77.1591
10	1176.36	79.6201	11	1140.69	76.6128	12	1109.83	81.5763
13	1033.66	85.5591	14	1006.66	83.8937	15	973.876	90.1702
16	838.883	94.396	17	760.78	96.6134	18	629.644	97.6737
19	454.154	96.9487	20	436.798	96.813	21	412.692	95.0446

Figure S26. IR Spectrum of **7**

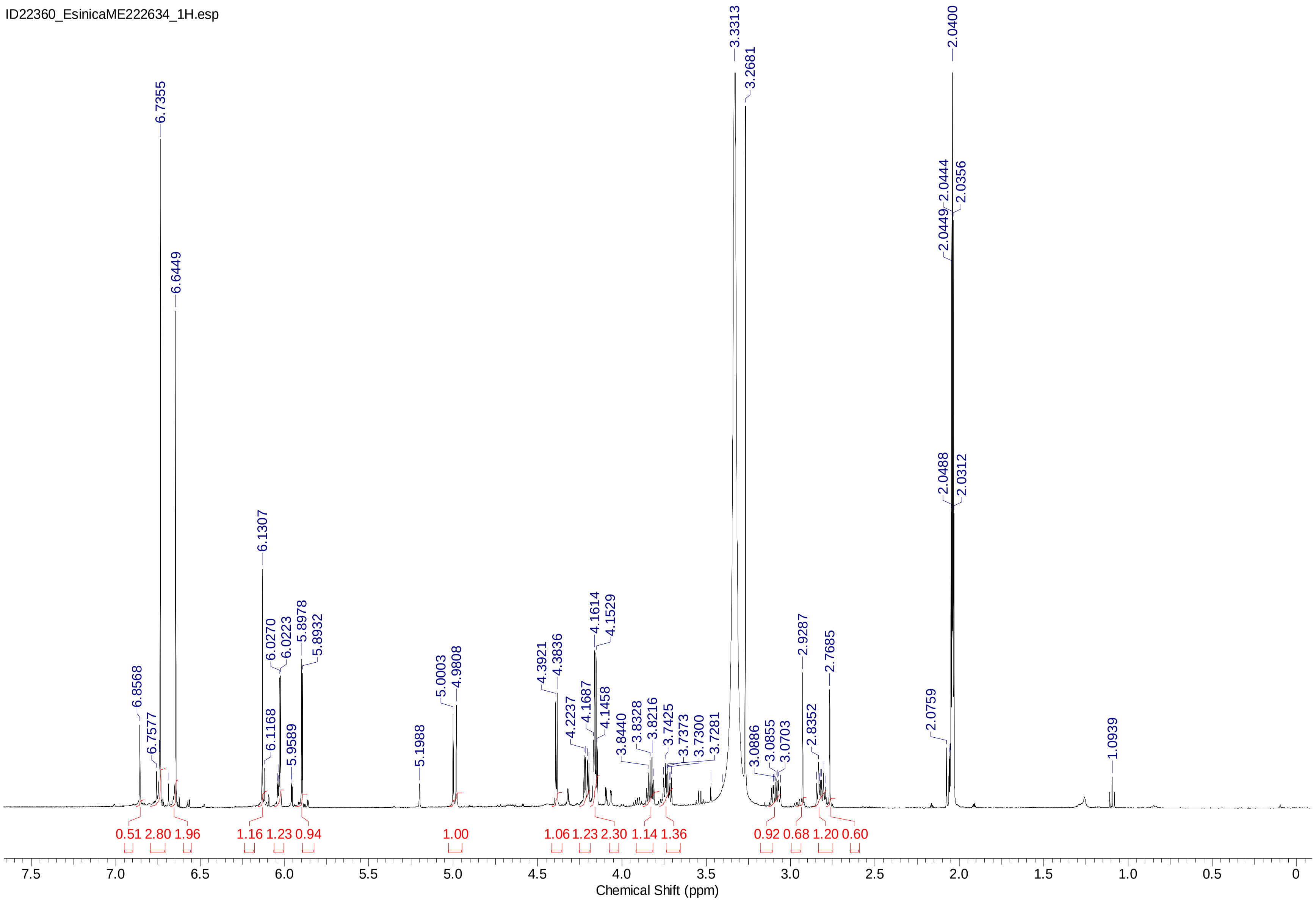

Figure S27. ^{1}H-NMR Spectrum of **7** in acetone-d_6-D_2O (500 MHz)

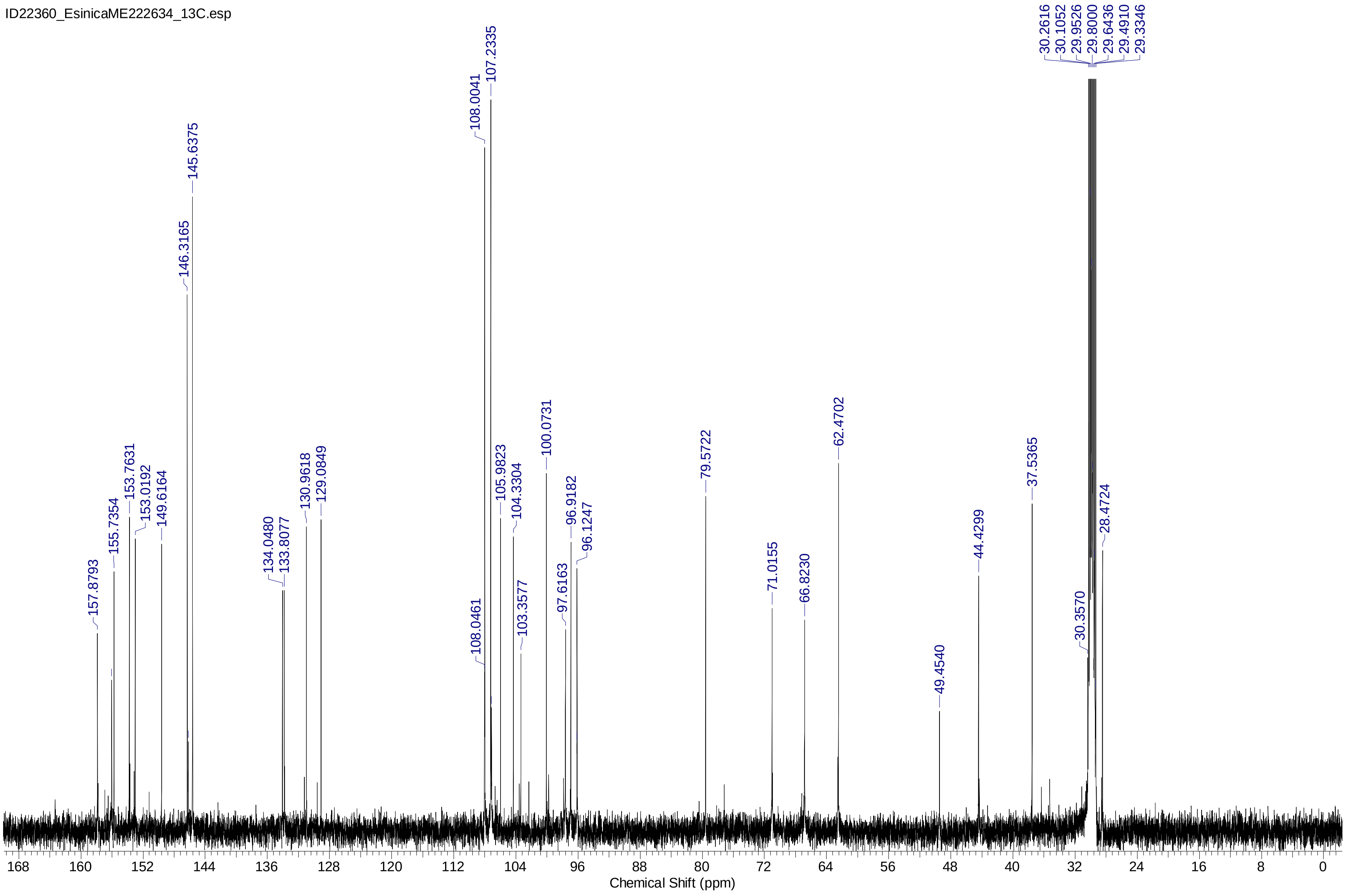

Figure S28. ^{13}C-NMR Spectrum of **7** in acetone-d_6-D_2O (125 MHz)

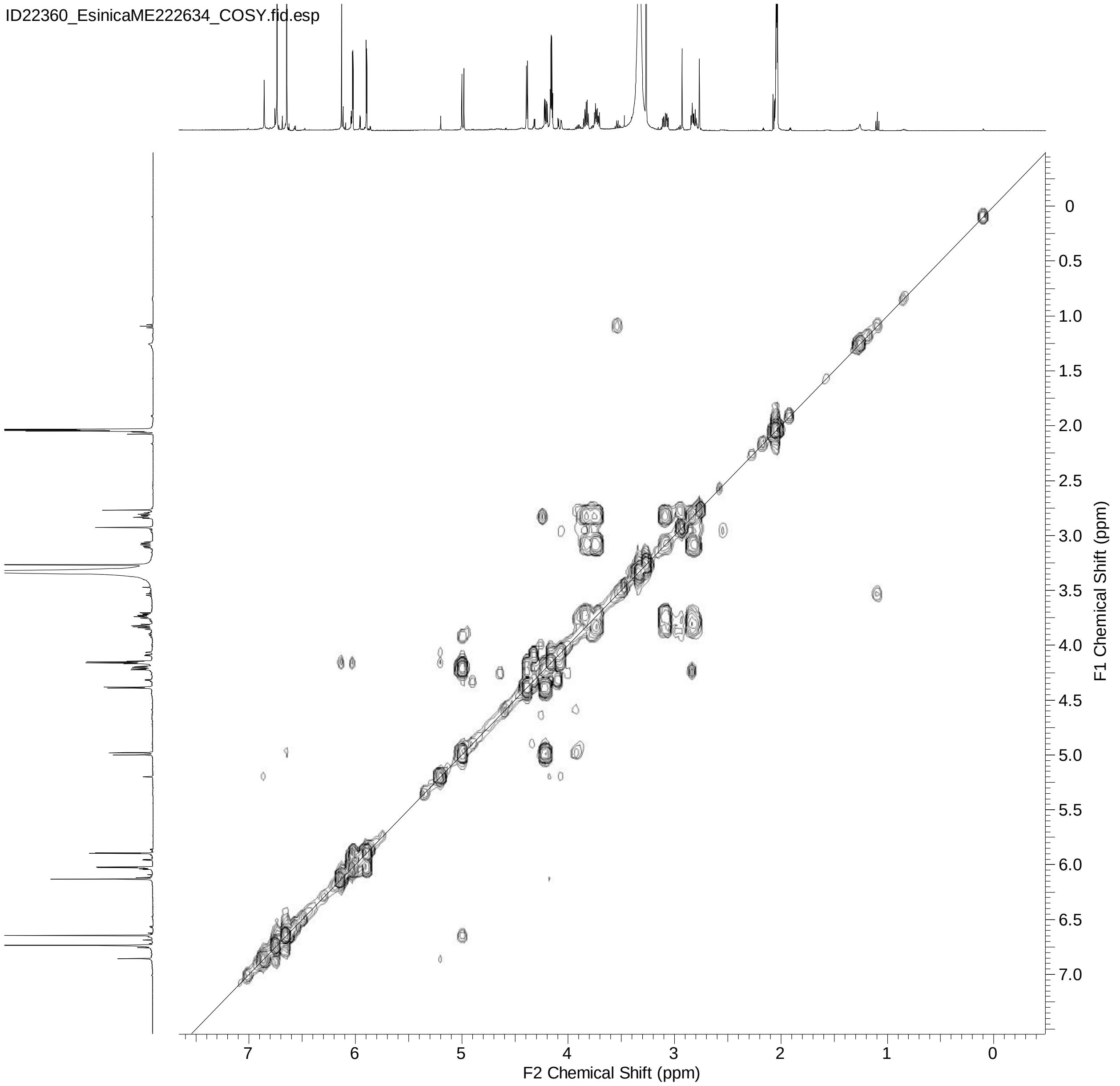

Figure S29. ^{1}H-^{1}H-COSY Spectrum of **7** in acetone-d_6-D_2O

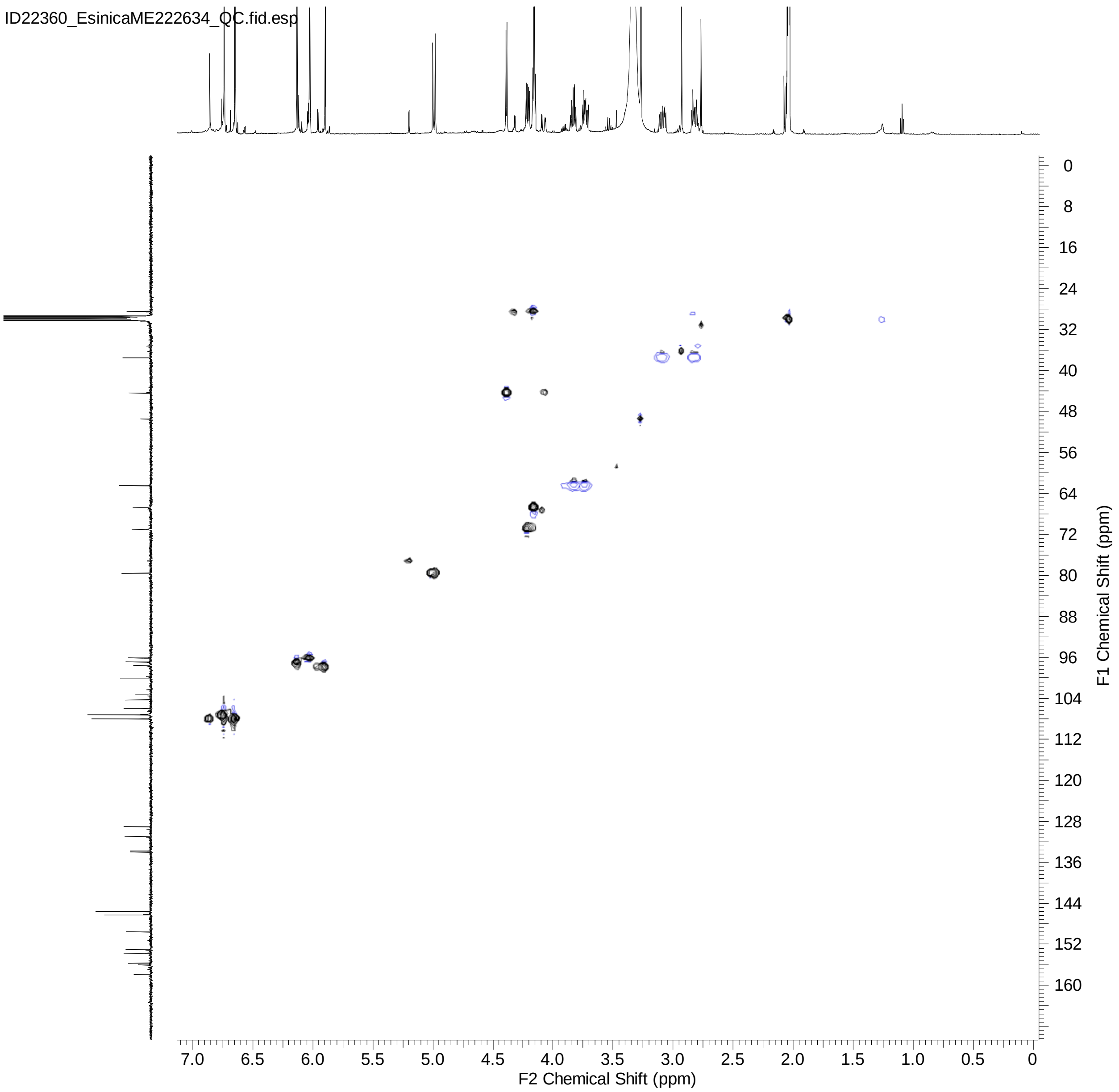

Figure S30. HSQC Spectrum of **7** in acetone-d_6-D_2O

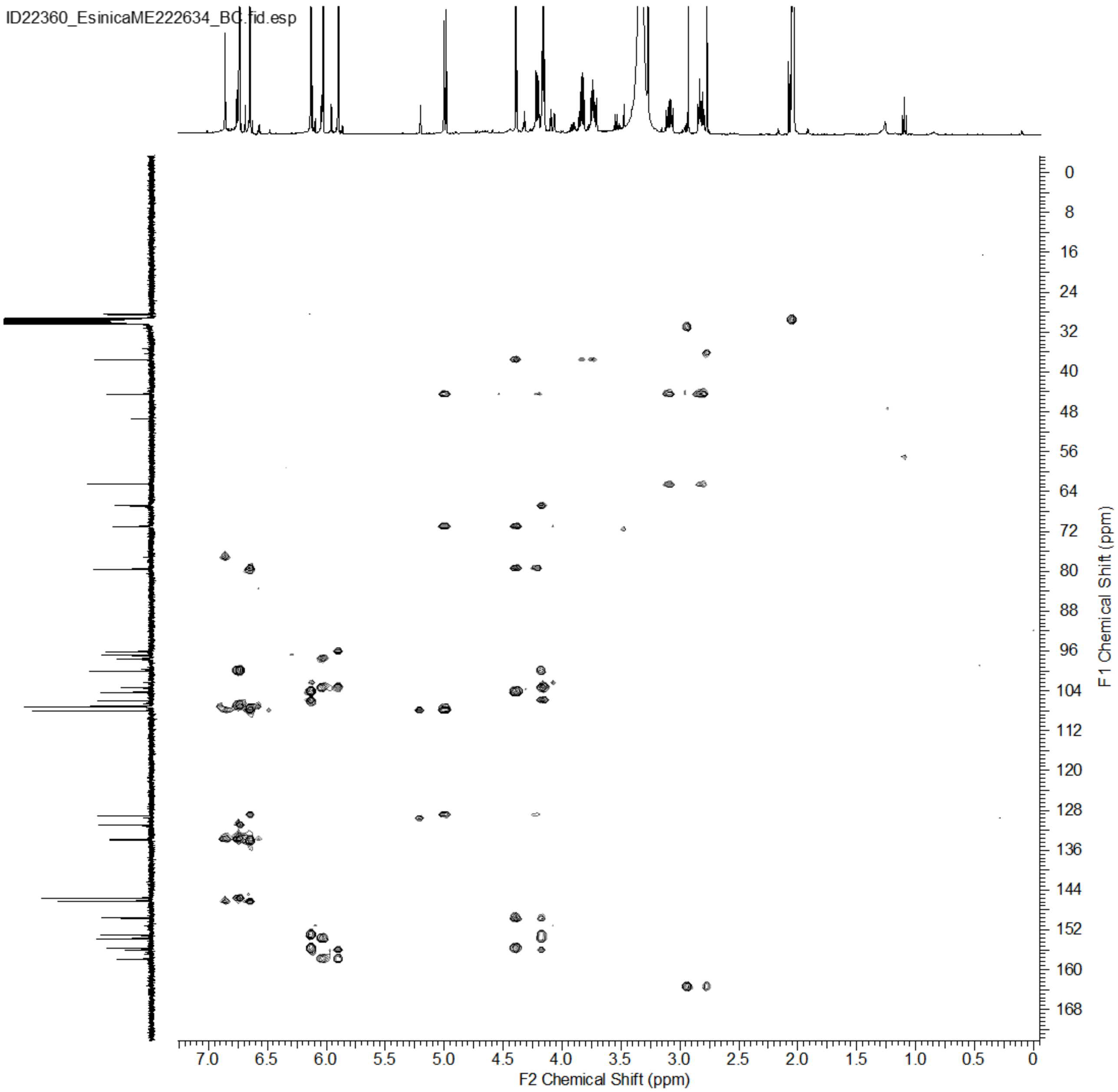

Figure S31. HMBC Spectrum of **7** in acetone-d_6-D_2O

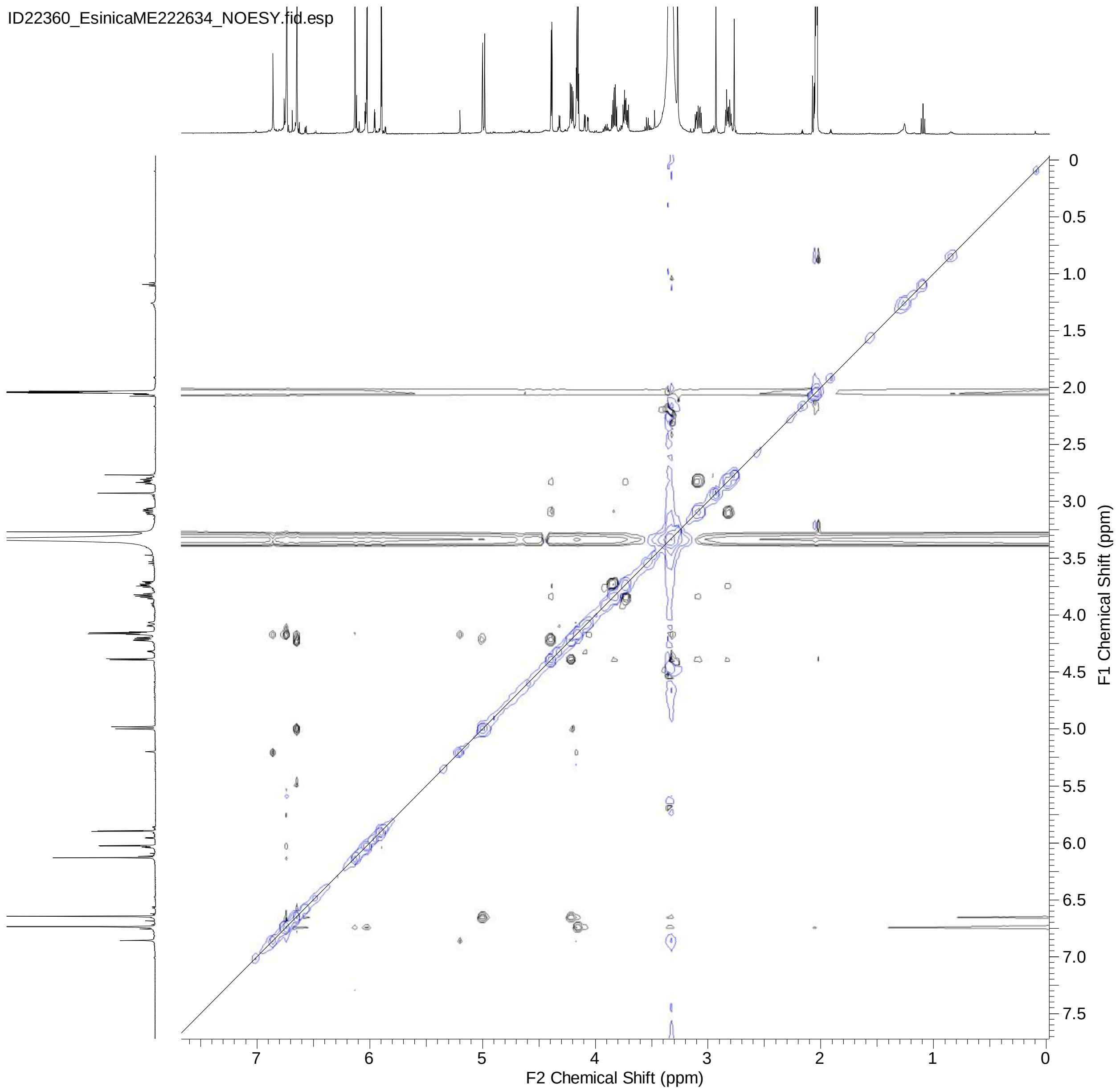

Figure S32. NOE Spectrum of **7** in acetone-d_6-D_2O

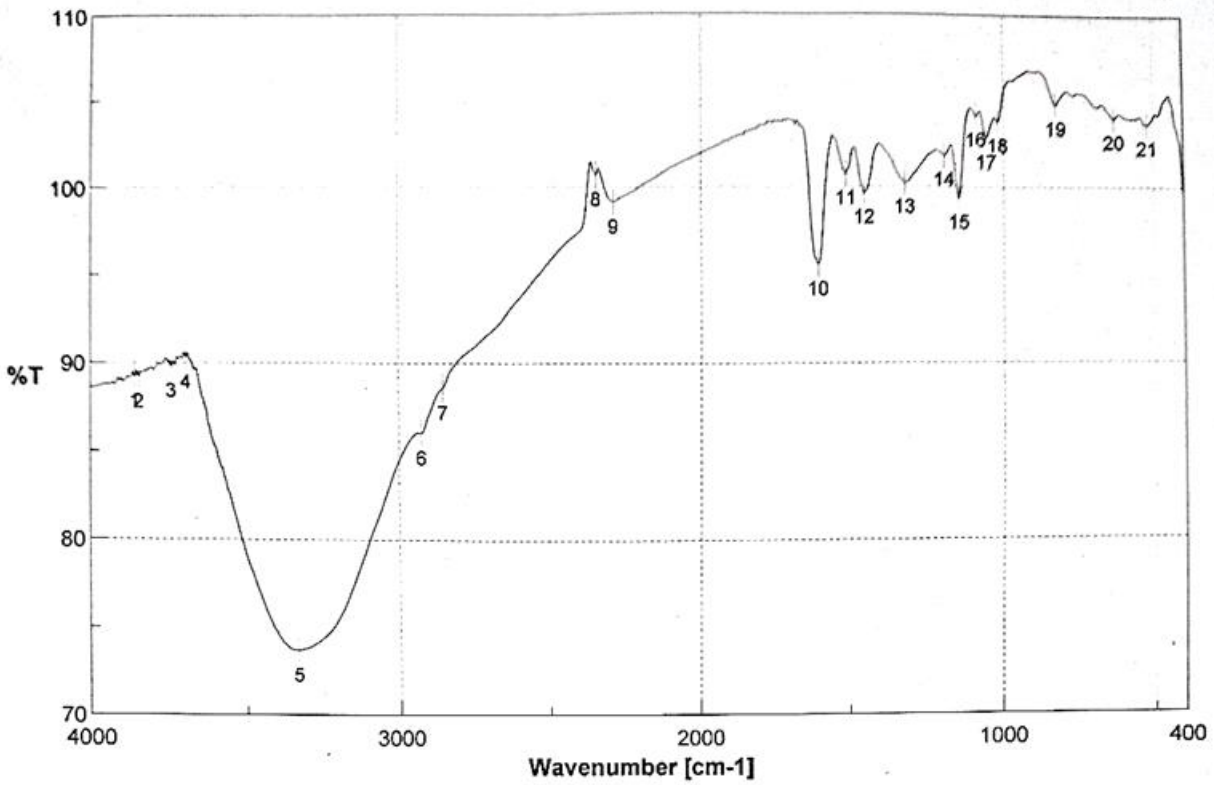

積算回数	Auto (34)
分解	4 cm-1
ゼロフィリング	ON
アポダイゼーション	Cosine
ゲイン	Auto (2)
スキャンスピード	Auto (2 mm/sec)
測定日時	2017/02/20 16:23
更新日時	2017/02/20 17:21
測定者	
ファイル名	E sinica Phl Degradation Fr 1-1-6-2 edited
サンプル名	
コメント	

No.	cm-1	%T	No.	cm-1	%T	No.	cm-1	%T
1	3855.97	89.2141	2	3843.43	89.2004	3	3738.33	89.8196
4	3691.09	90.3163	5	3333.36	73.7107	6	2925.48	86.0531
7	2854.13	88.6019	8	2345.98	100.711	9	2289.09	99.1621
10	1614.13	95.5842	11	1522.52	100.754	12	1462.74	99.6633
13	1328.71	100.289	14	1197.58	101.837	15	1149.37	99.3773
16	1091.51	104.137	17	1057.76	102.845	18	1018.23	103.803
19	824.42	104.794	20	630.609	104.029	21	519.722	103.672

Figure S33. IR Spectrum of **13**

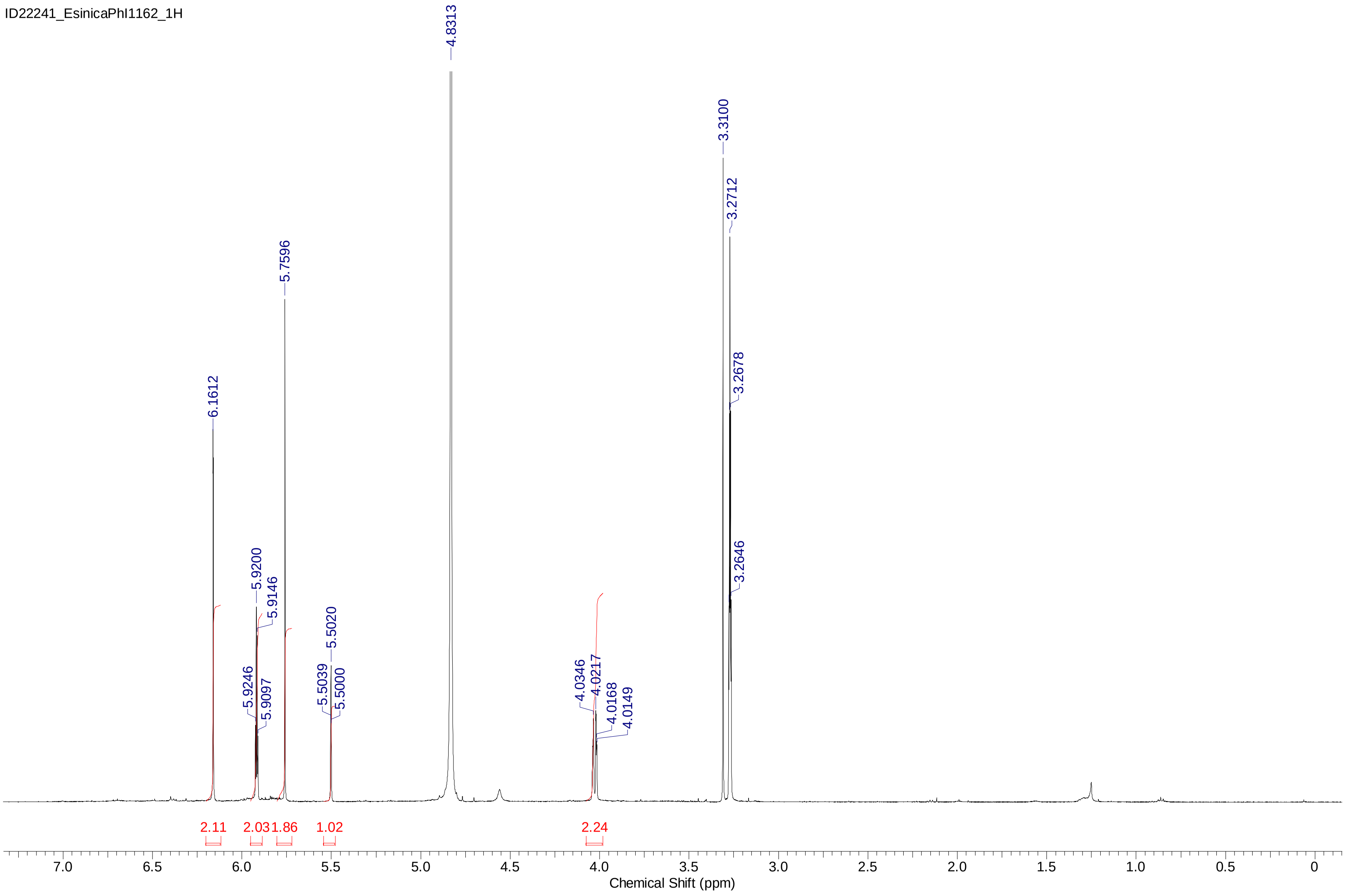

Figure S34. ^{1}H-NMR Spectrum of **13** in CD_3OD (500 MHz)

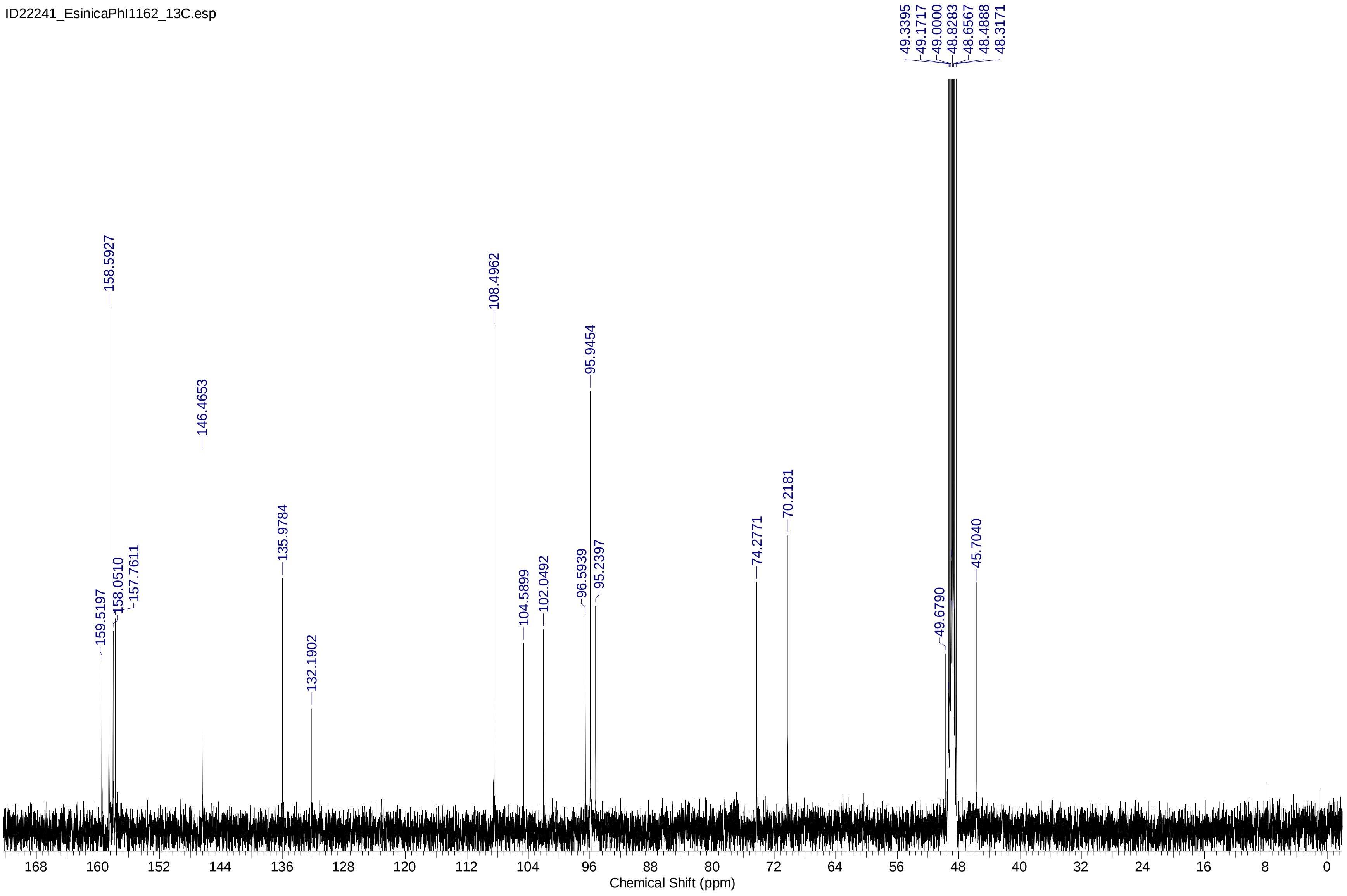

Figure S35. ^{13}C-NMR Spectrum of **13** in CD_3OD (125 MHz)

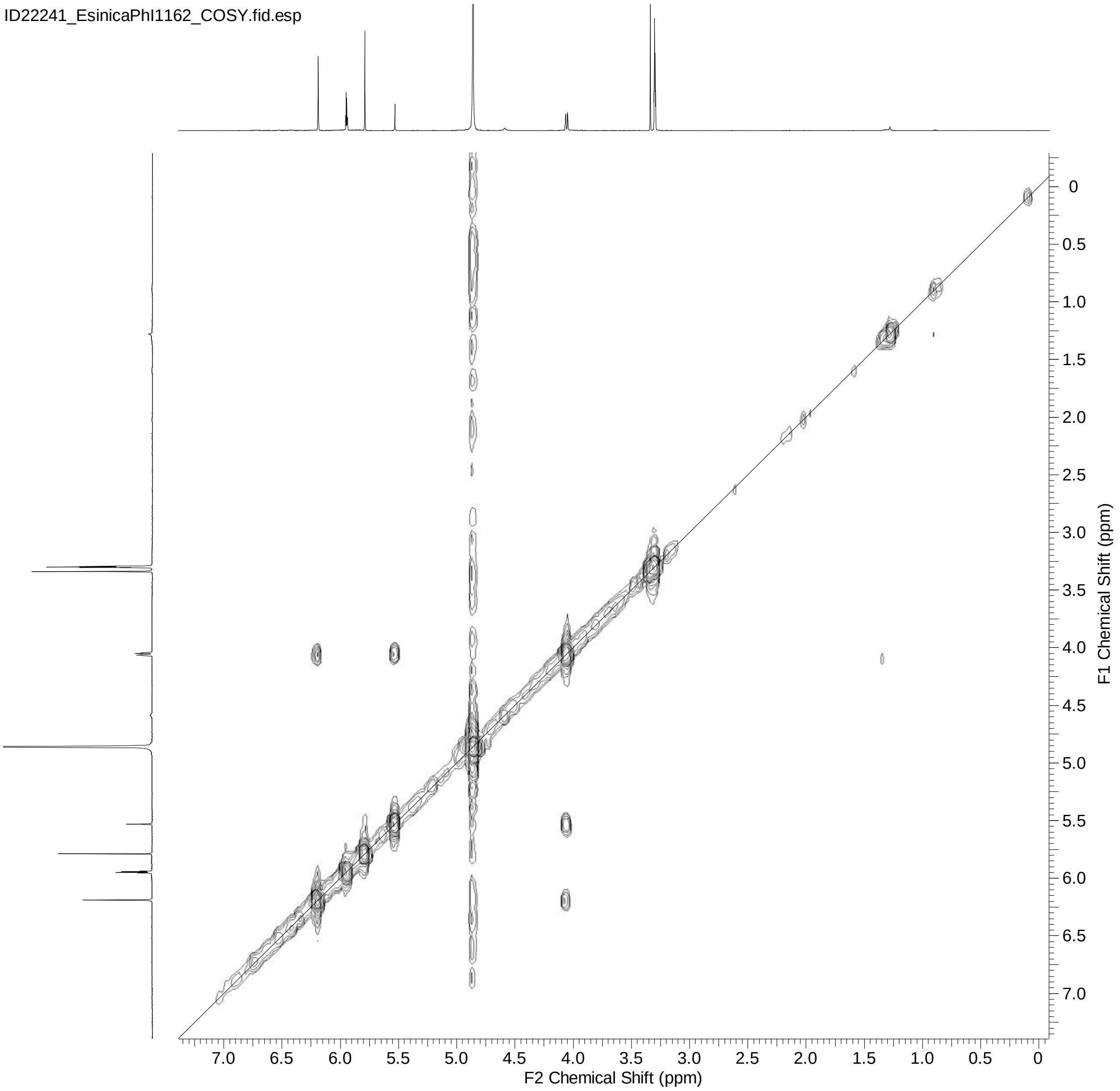

Figure S36. ^{1}H-^{1}H-COSY Spectrum of **13** in CD_3OD

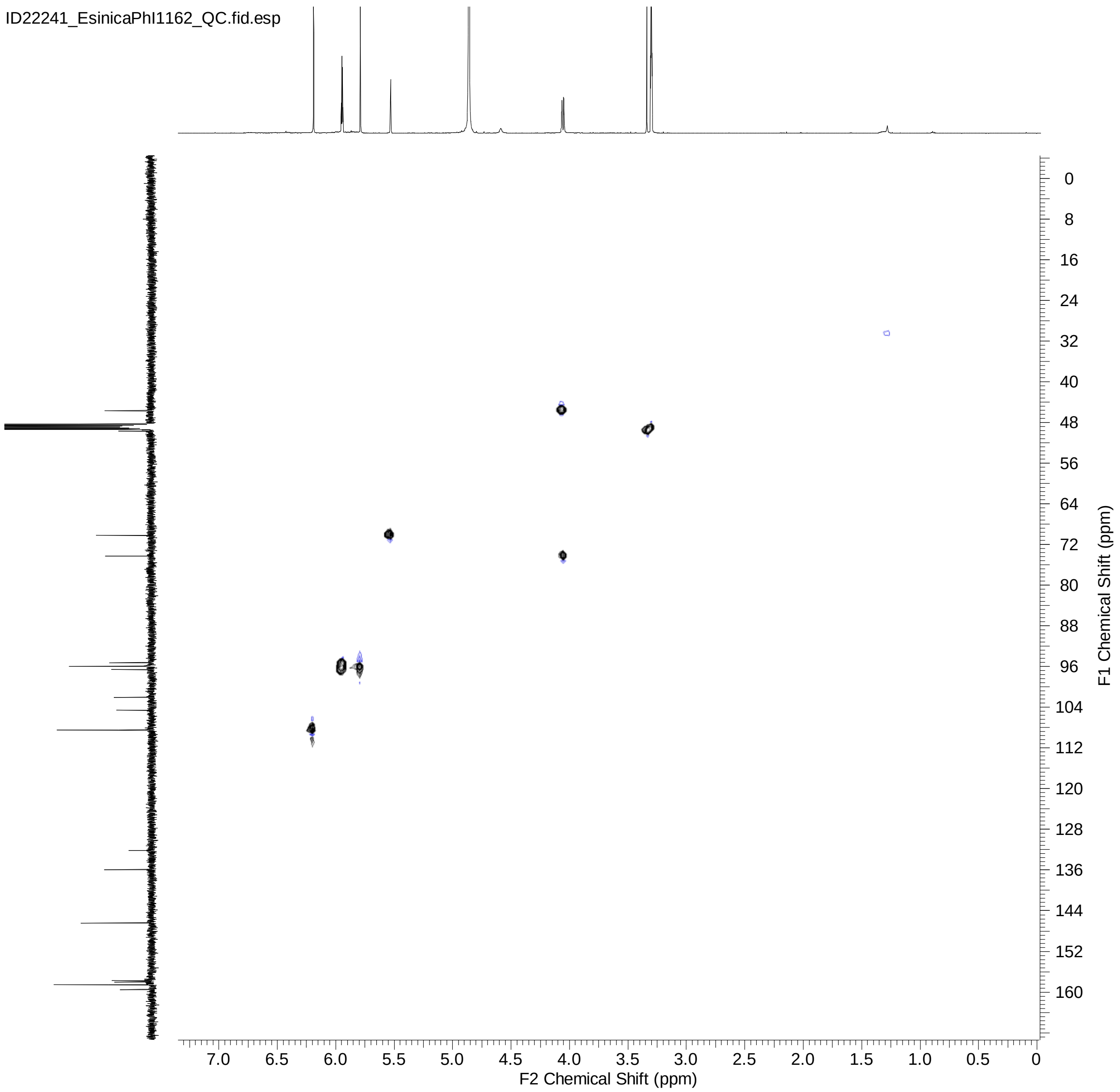

Figure S37. HSQC Spectrum of **13** in CD_3OD

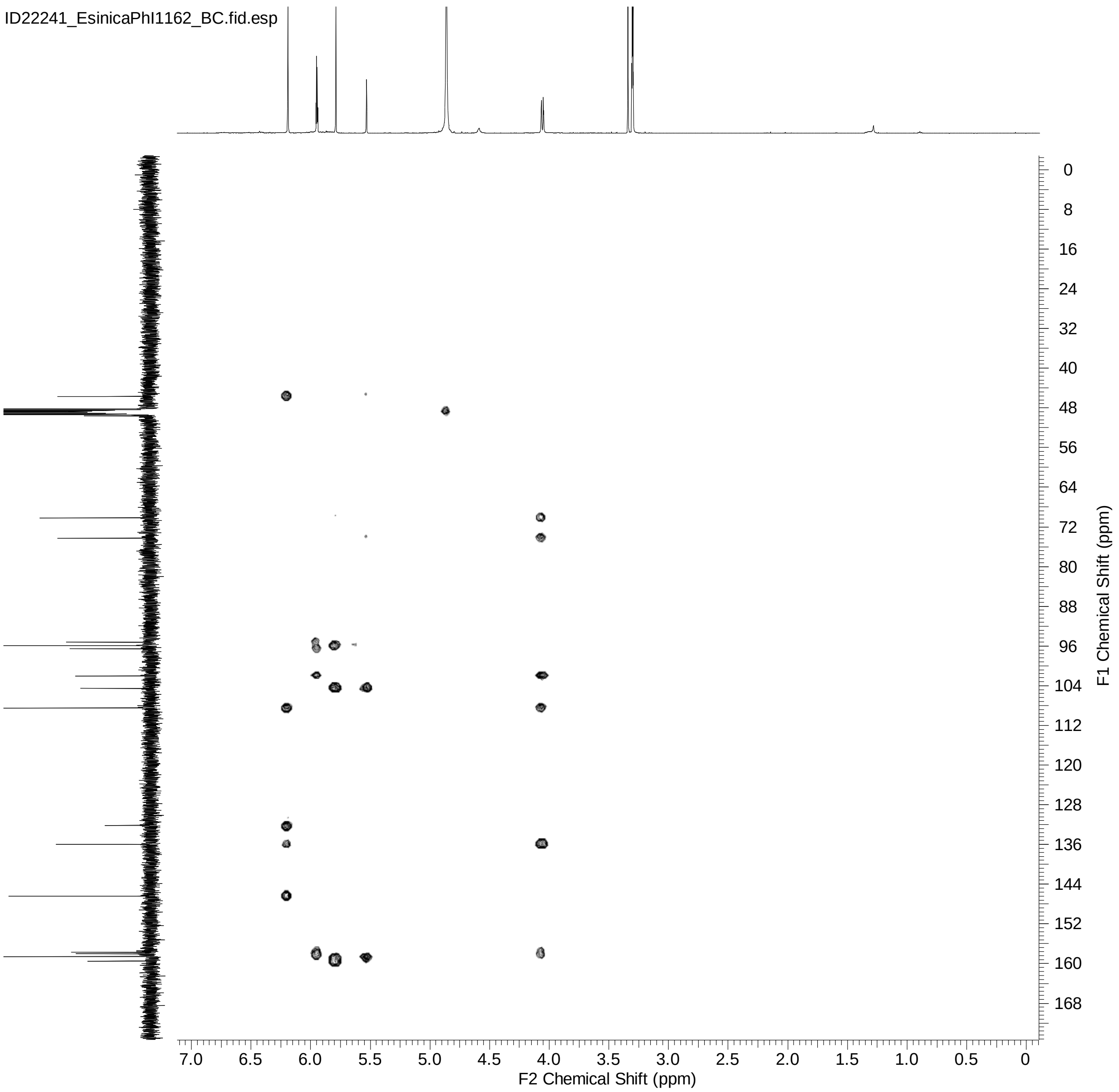

Figure S38. HMBC Spectrum of **13** in CD_3OD

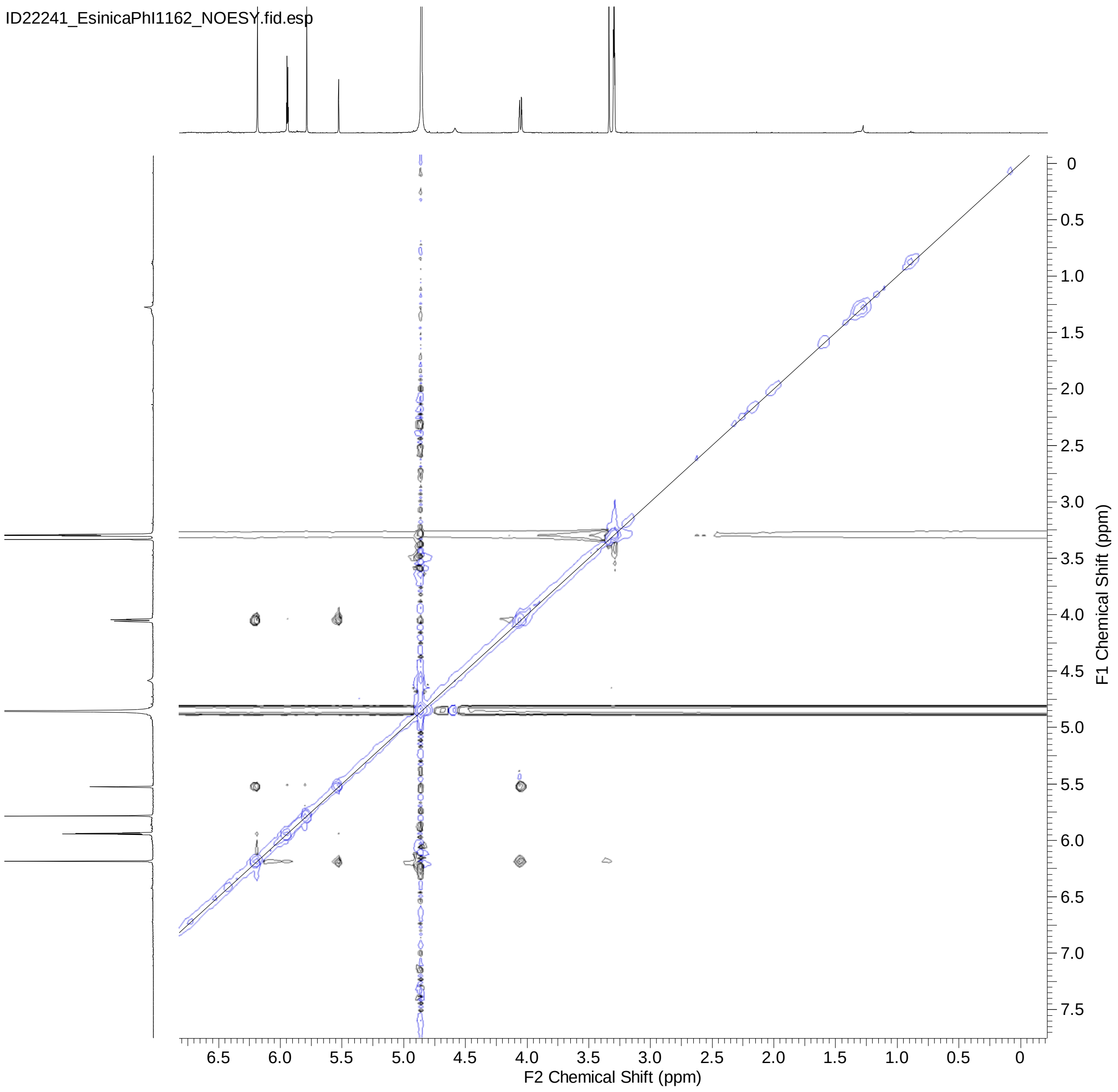

Figure S39. NOE Spectrum of **13** in CD_3OD

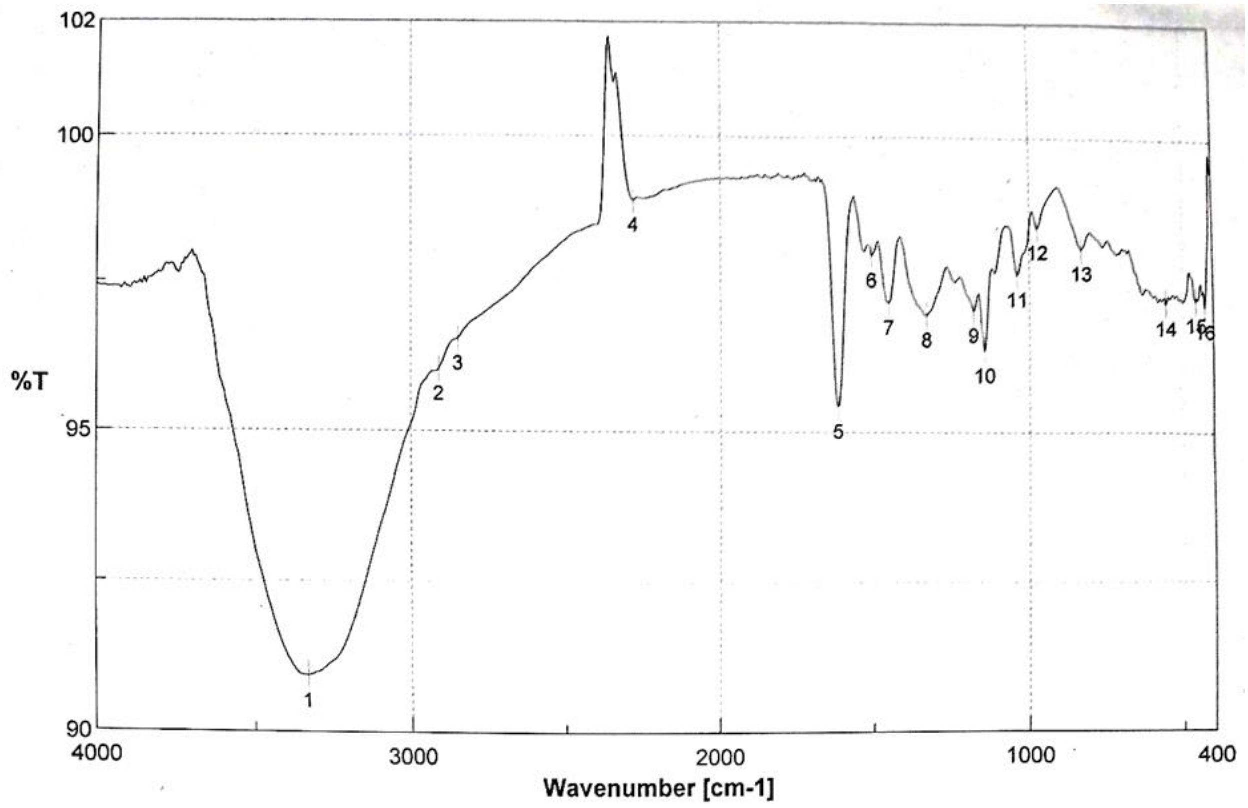

積算回数	Auto (30)
分解	4 cm-1
ゼロフィリング	ON
アポダイゼーション	Cosine
ゲイン	Auto (2)
スキャンスピード	Auto (2 mm/sec)
測定日時	2017/02/20 16:46
更新日時	2017/02/20 16:56
測定者	
ファイル名	E sinica Phl Degradation Fr 1-1-8-1-2-1 edited
サンプル名	
コメント	

No.	cm-1	%T	No.	cm-1	%T	No.	cm-1	%T
1	3333.36	90.944	2	2910.06	96.0441	3	2848.35	96.5504
4	2281.38	98.9038	5	1619.91	95.437	6	1510.95	97.976
7	1455.03	97.1859	8	1332.57	96.9762	9	1180.22	97.0574
10	1145.51	96.3834	11	1038.48	97.658	12	972.912	98.4589
13	830.205	98.103	14	556.363	97.1754	15	457.047	97.2362
16	428.12	97.1318						

Figure S40. IR Spectrum of **14**

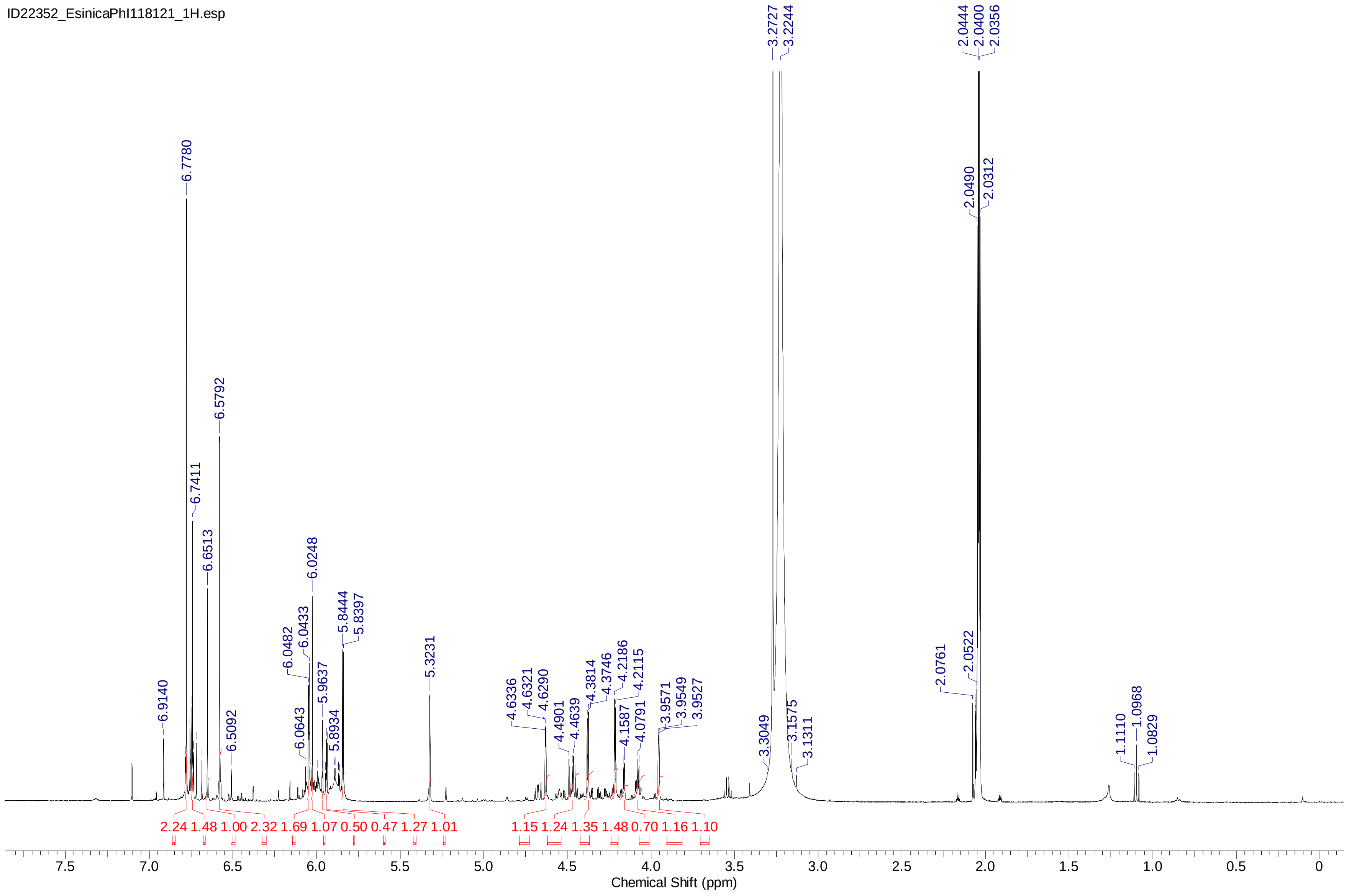

Figure S41. ^{1}H-NMR Spectrum of **14** in acetone-d_6-D_2O (500 MHz)

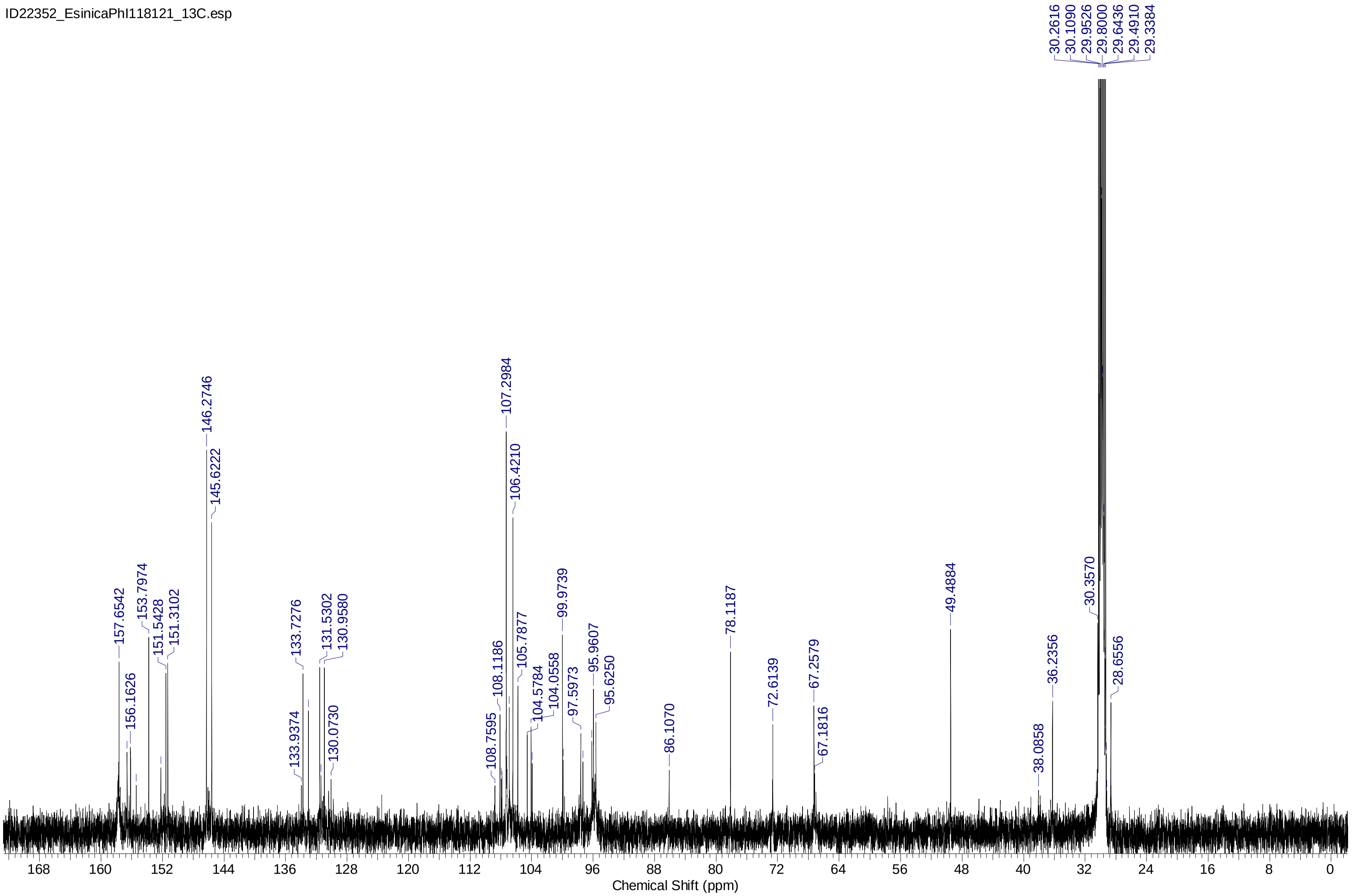

Figure S42. ^{13}C-NMR Spectrum of **14** in acetone-d_6-D_2O (125 MHz)

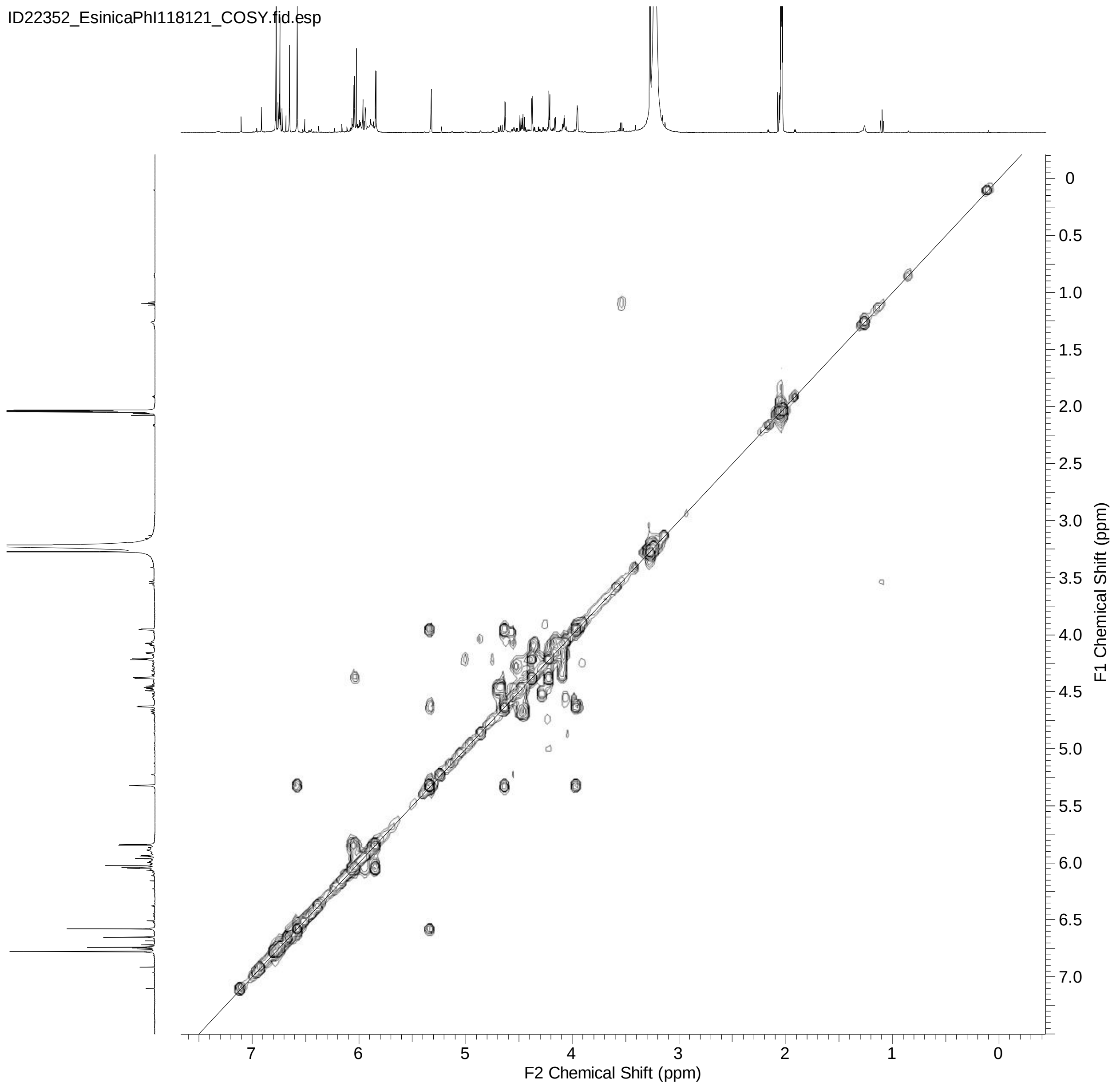

Figure S43. ^{1}H-^{1}H-COSY Spectrum of **14** in acetone-d_6-D_2O

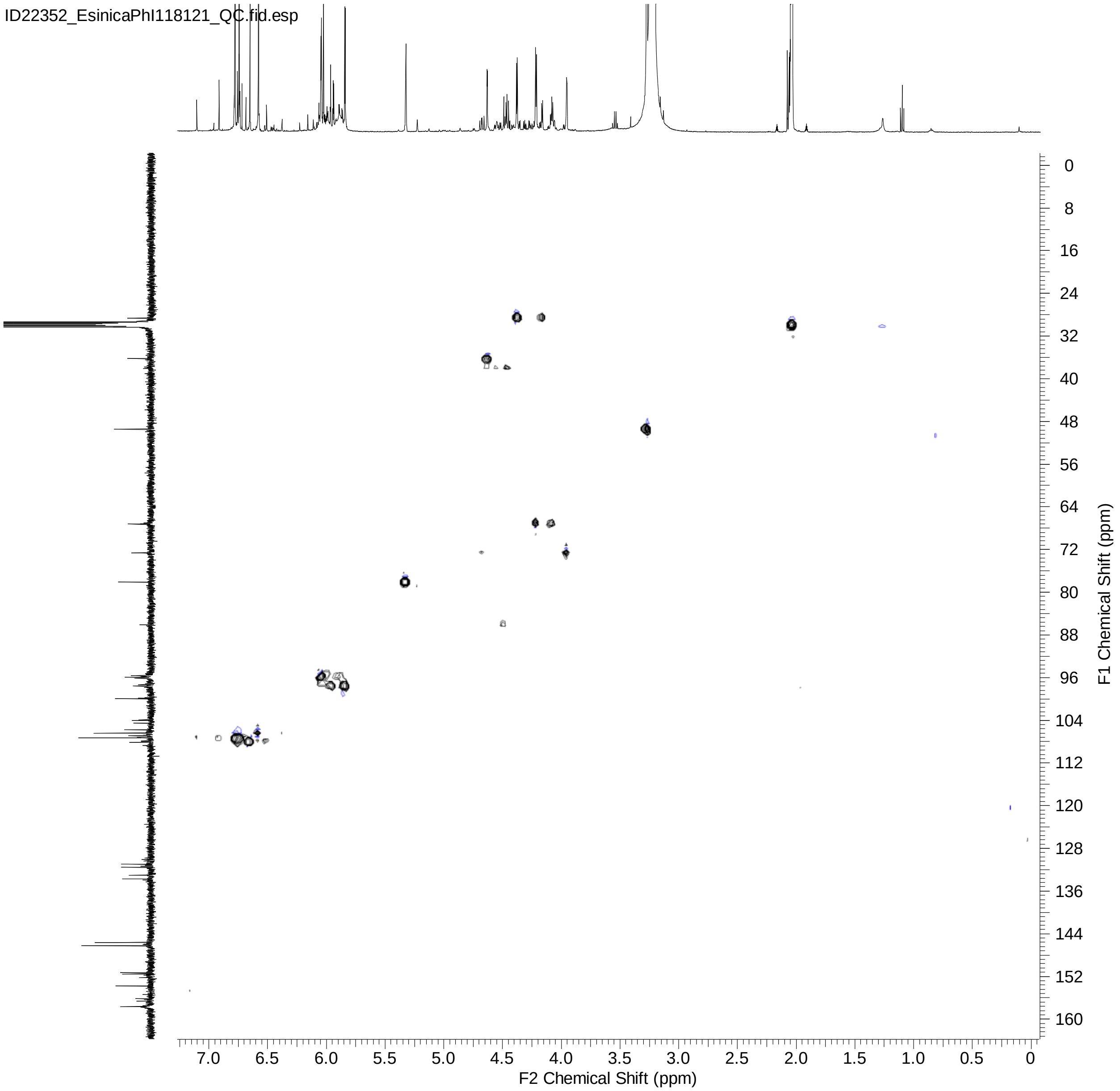

Figure S44. HSQC Spectrum of **14** in acetone-d_6-D_2O

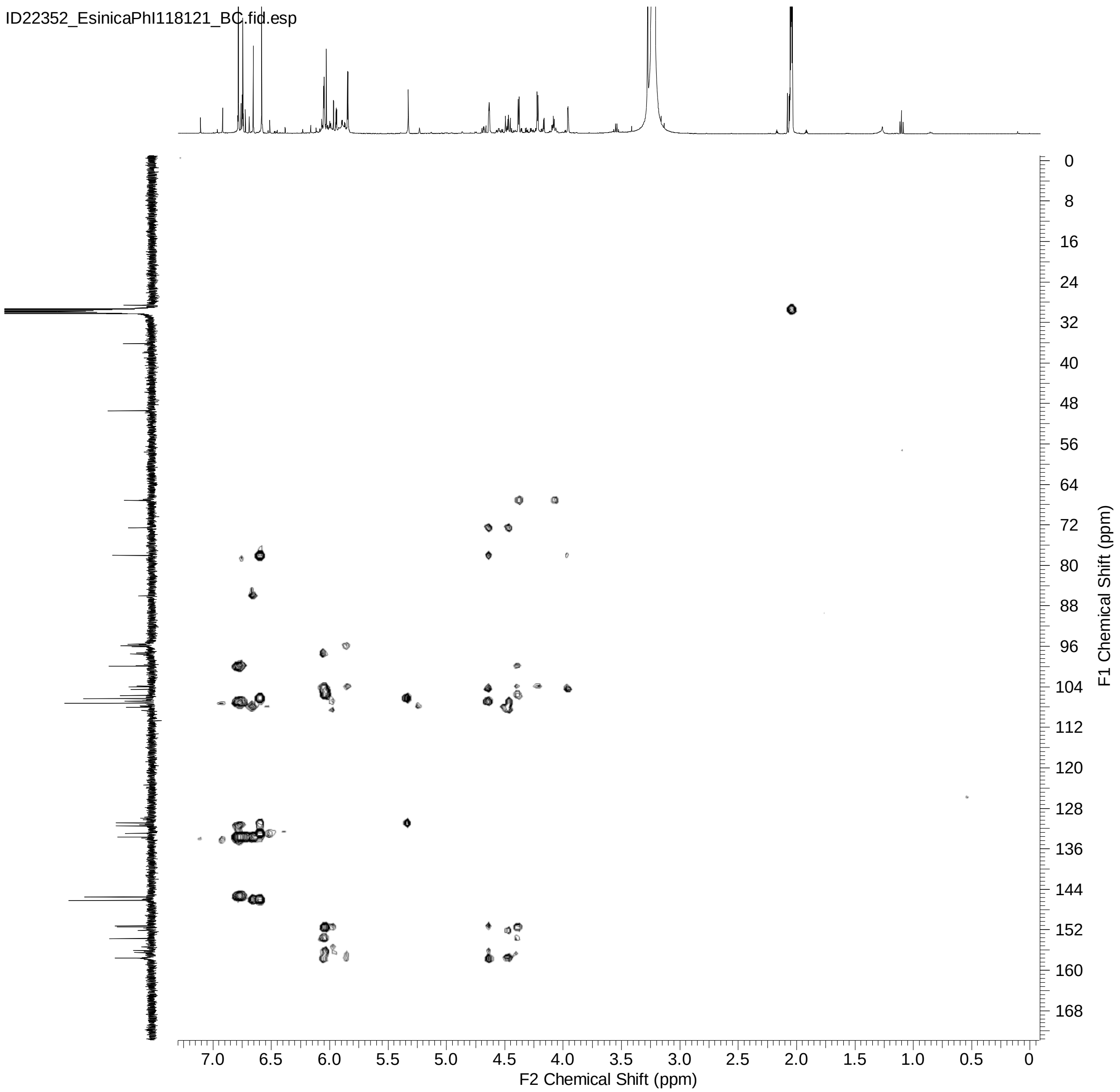

Figure S45. HMBC Spectrum of **14** in acetone-d_6-D_2O

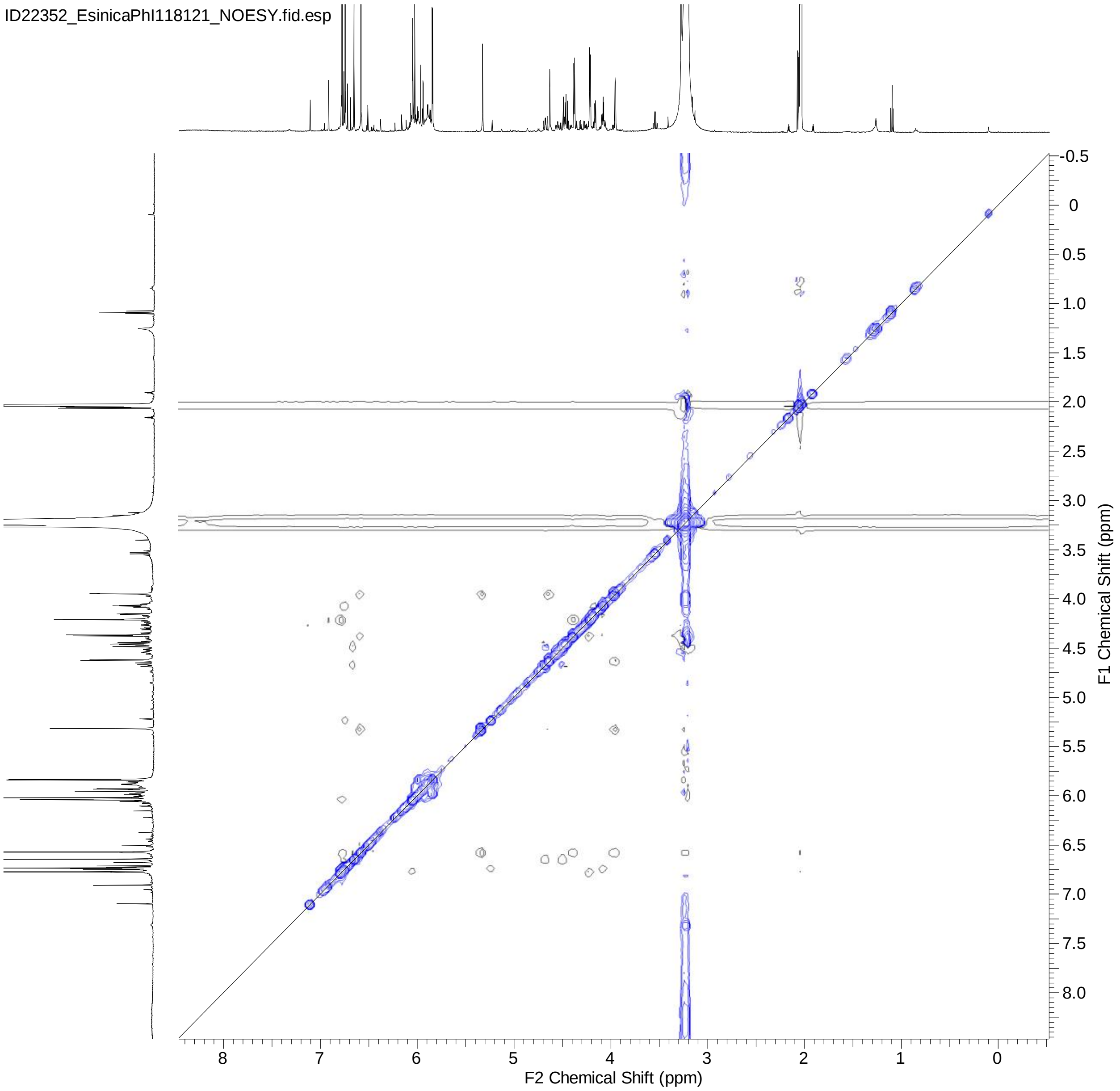

Figure S46. NOE Spectrum of **14** in acetone-d_6-D_2O

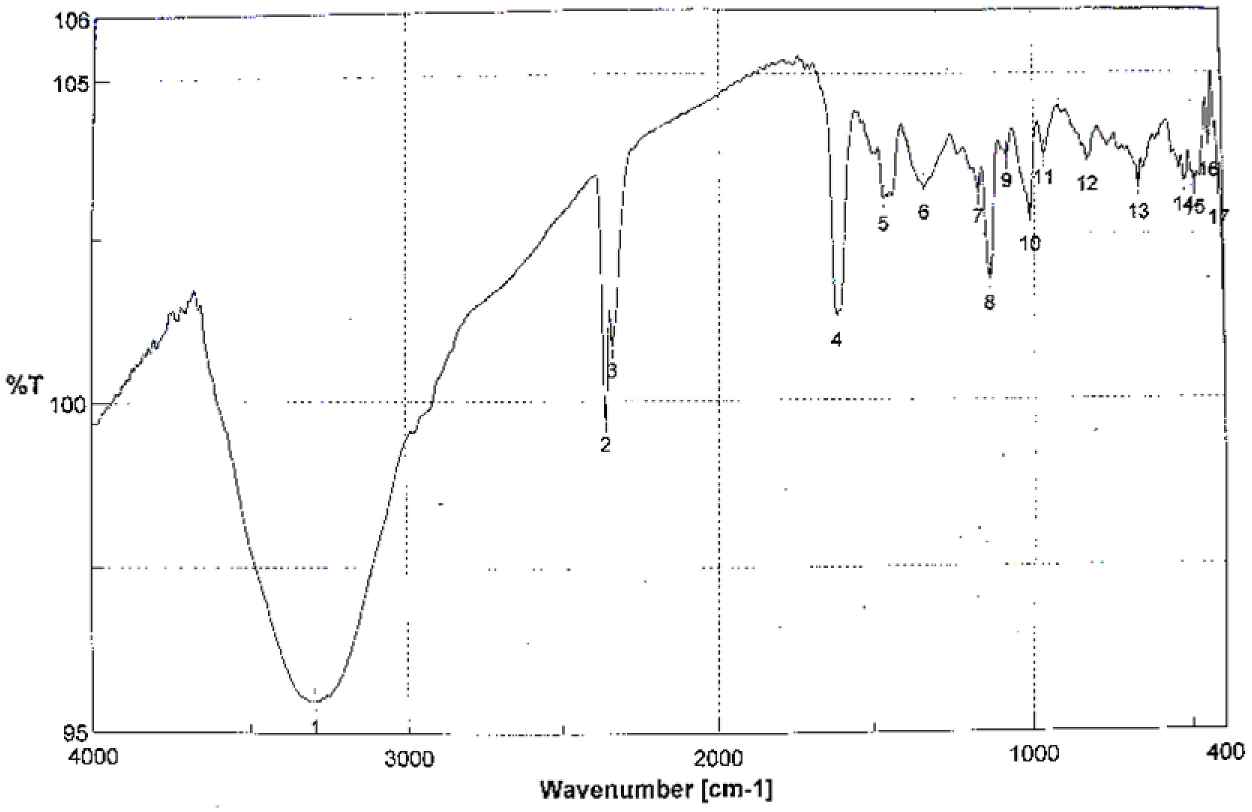

積算回数	Auto (32)
分解	4 cm-1
ゼロフィリング	ON
アポダイゼーション	Cosine
ゲイン	Auto (2)
スキャンスピード	Auto (2 mm/sec)
測定日時	2017/02/20 15:54
更新日時	2017/02/20 17:19
測定者	
ファイル名	E sinica PhI Degradation Fr 1-1-5-5-2 edited
サンプル名	
コメント	

No.	cm-1	%T	No.	cm-1	%T	No.	cm-1	%T
1	3296.71	95.4833	2	2360.44	99.7365	3	2340.19	100.841
4	1625.7	101.288	5	1476.24	103.065	6	1347.03	103.23
7	1176.36	103.16	8	1141.65	101.841	9	1084.76	103.703
10	1013.41	102.725	11	968.09	103.747	12	831.169	103.645
13	669.178	103.208	14	524.543	103.322	15	491.759	103.281
16	443.547	103.864	17	413.656	103.127			

Figure S47. IR Spectrum of **16**

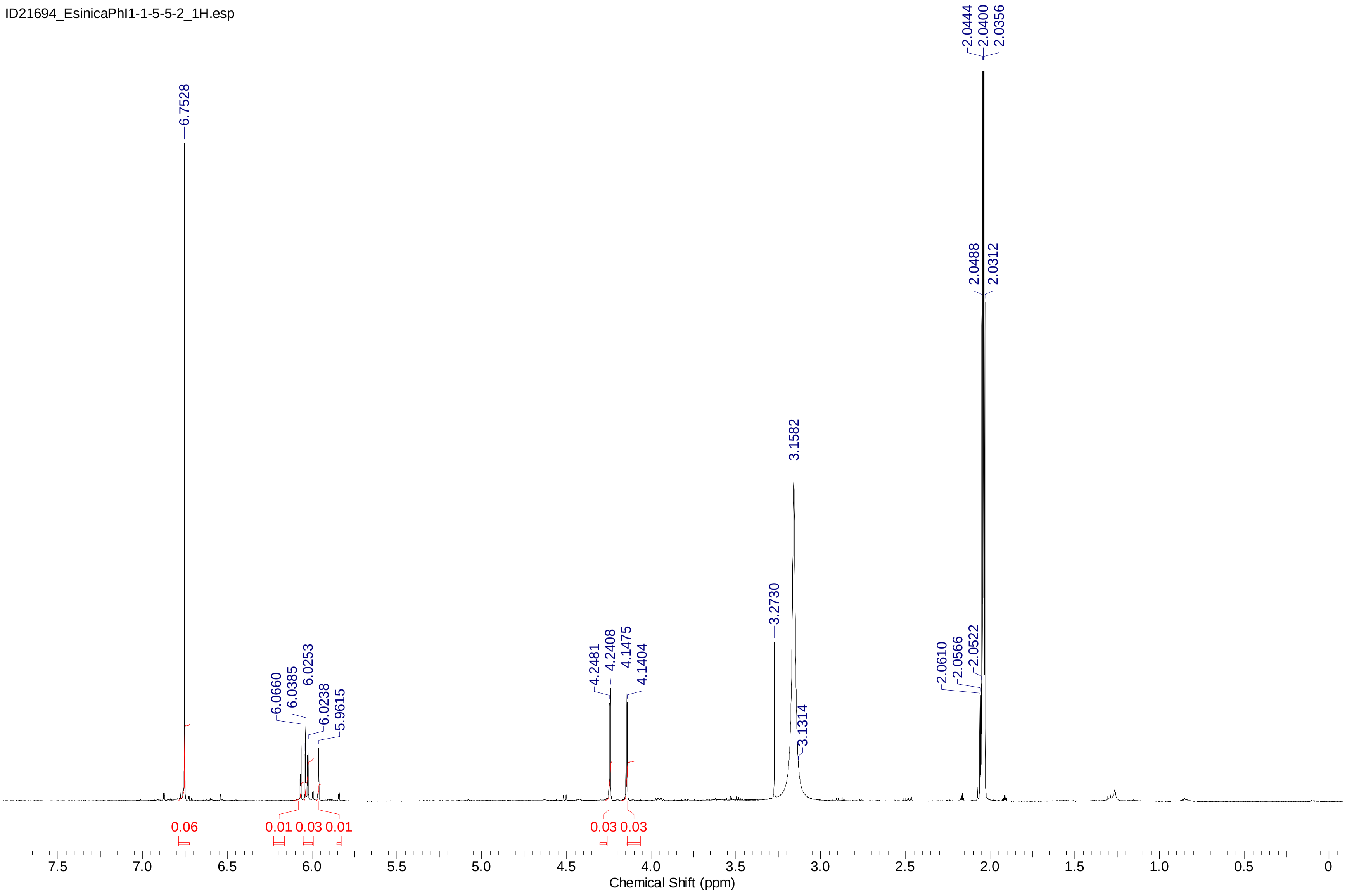

Figure S48. ^{1}H-NMR Spectrum of **16** in acetone-d_6-D_2O (500 MHz)

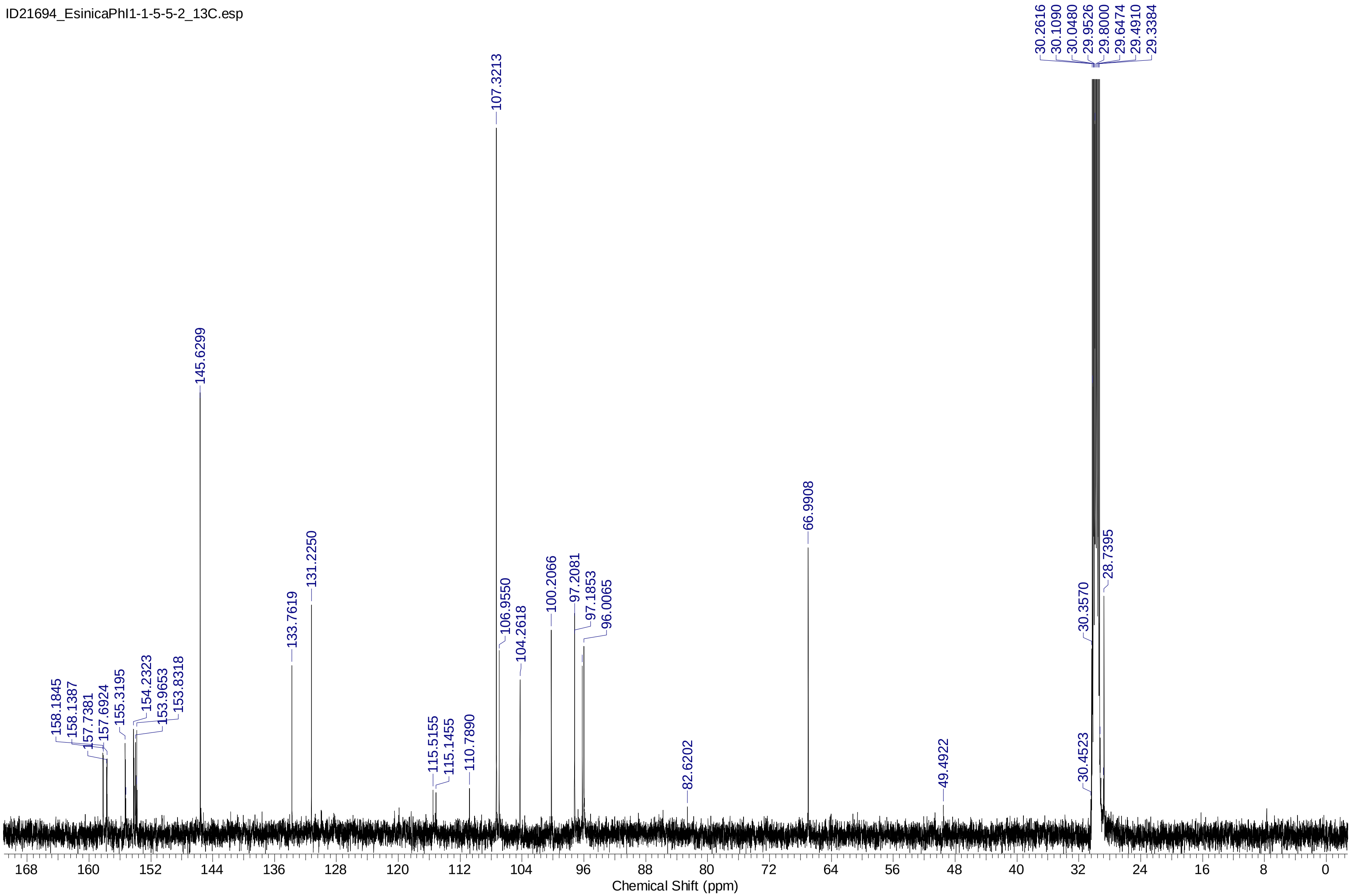

Figure S49. ^{13}C-NMR Spectrum of **16** in acetone-d_6-D_2O (125 MHz)

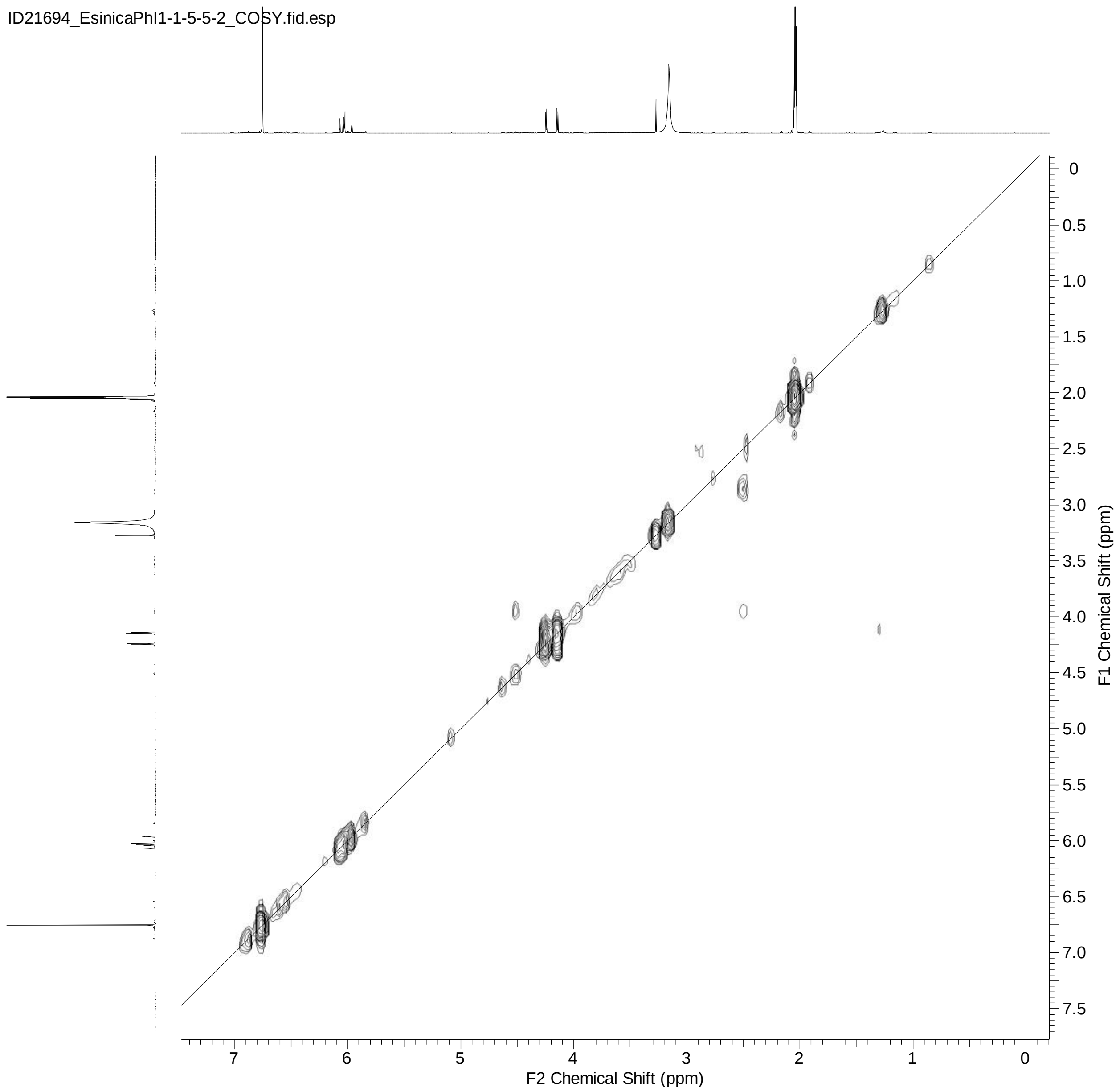

Figure S50. ^{1}H-^{1}H-COSY Spectrum of **16** in acetone-d_6-D_2O

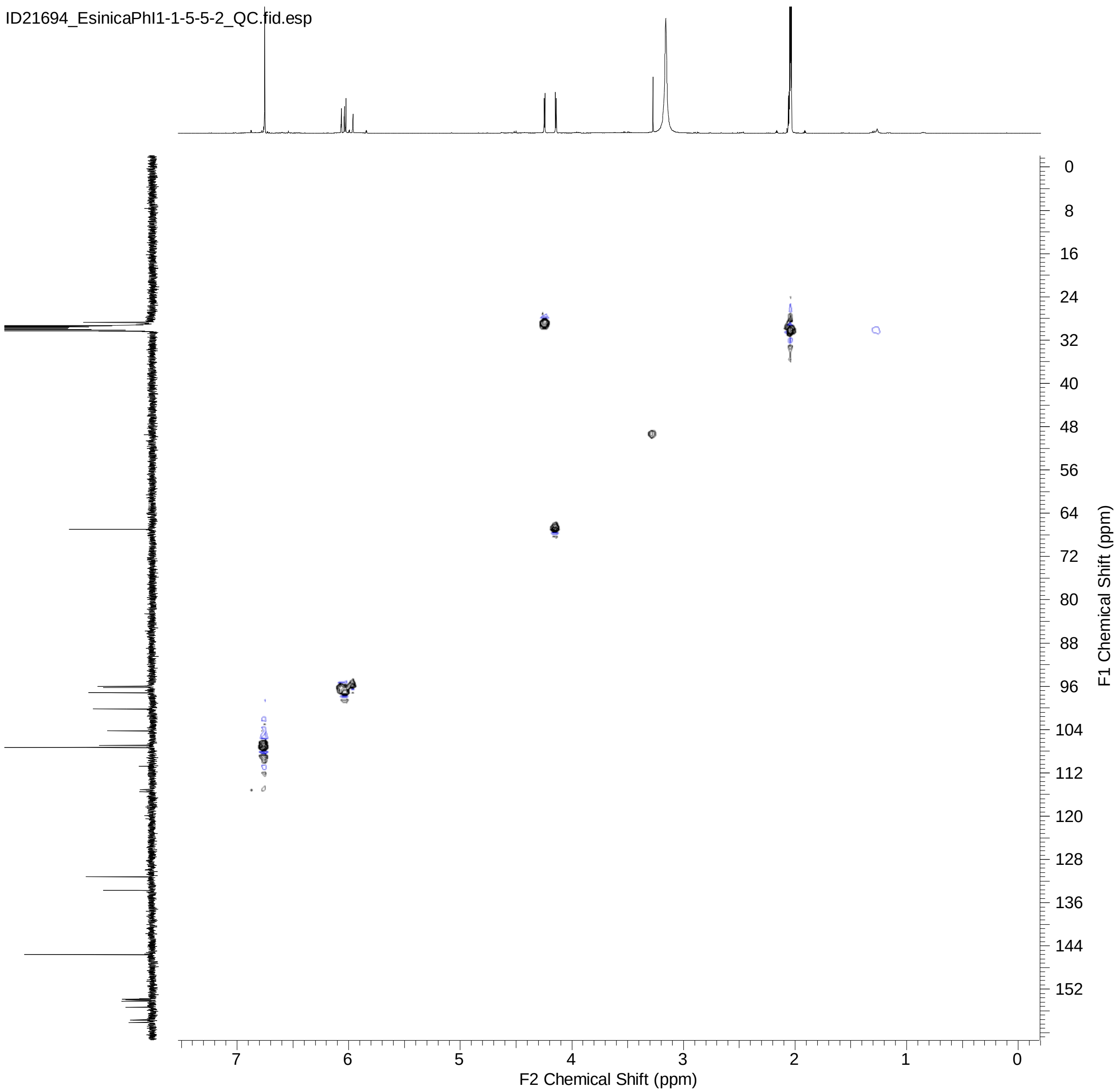

Figure S51. HSQC Spectrum of **16** in acetone-d_6-D_2O

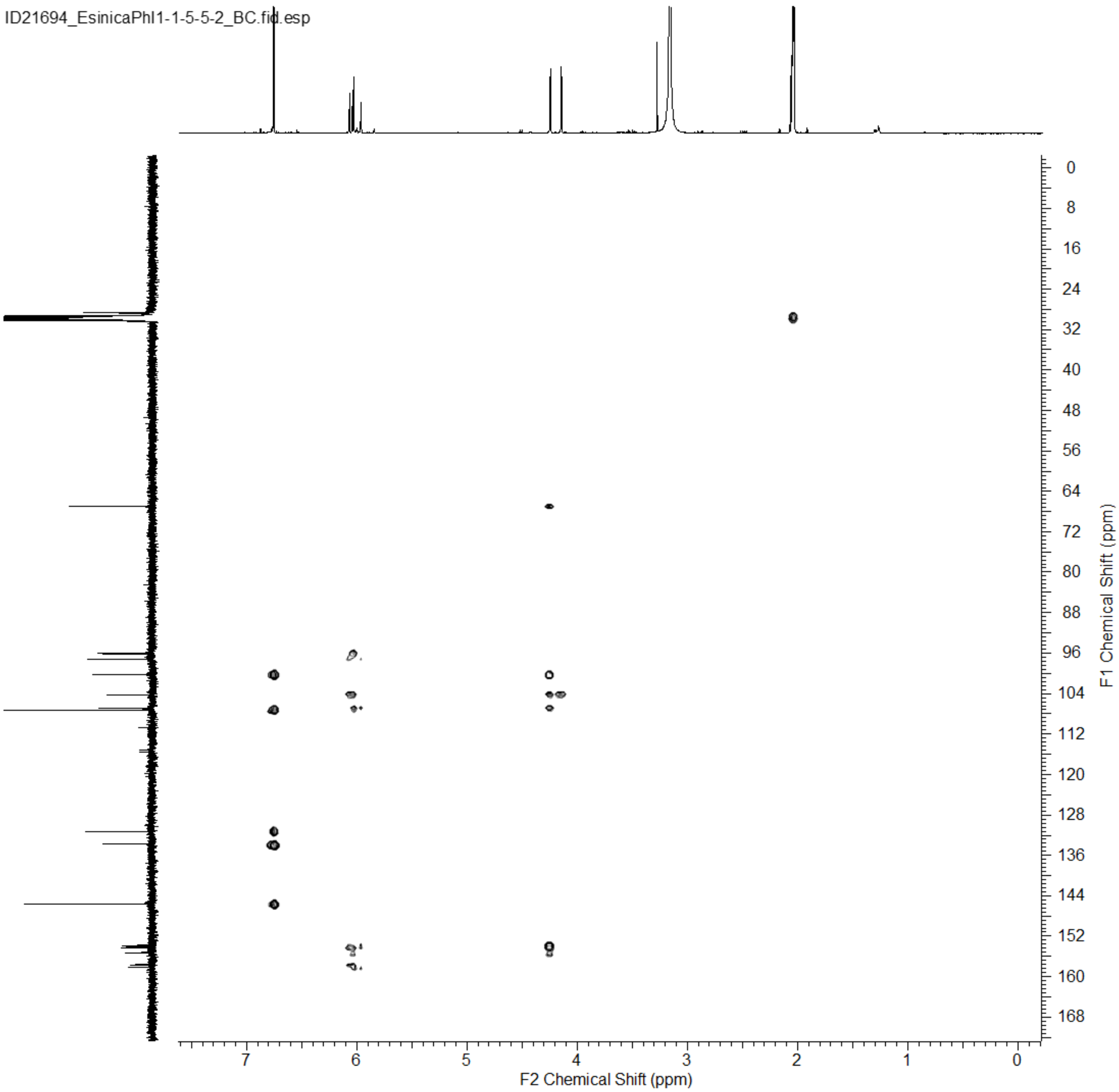

Figure S52. HMBC Spectrum of **16** in acetone-d_6-D_2O

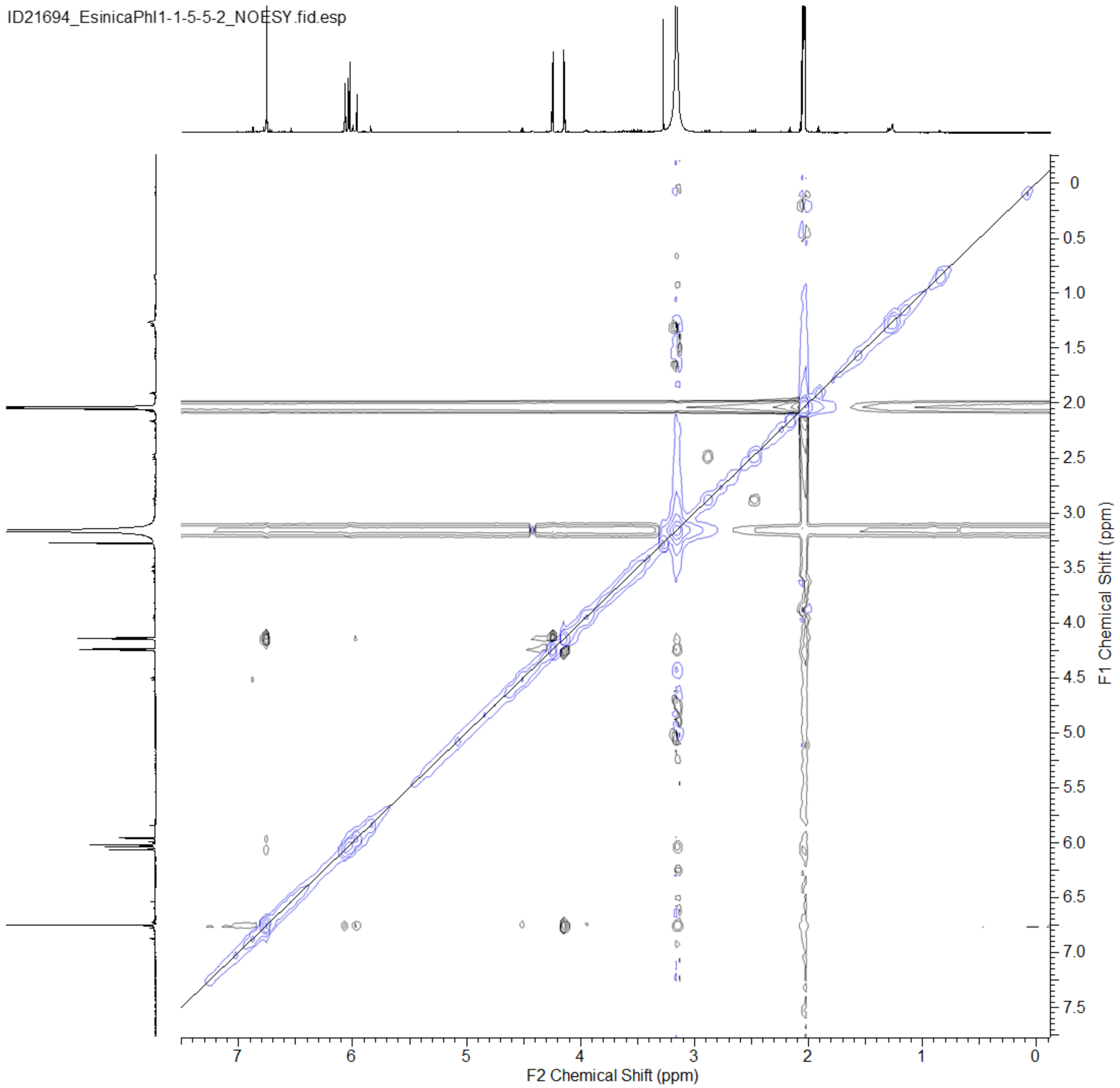

Figure S53. NOE Spectrum of **16** in acetone-d_6-D_2O
